# Trait convergence and trait divergence in lake phytoplankton reflect community assembly rules

**DOI:** 10.1038/s41598-020-76645-7

**Published:** 2020-11-11

**Authors:** Gábor Borics, Viktória B-Béres, István Bácsi, Balázs A. Lukács, E. T-Krasznai, Zoltán Botta-Dukát, Gábor Várbíró

**Affiliations:** 1 Department of Tisza Research, Danube Research Institute, Centre for Ecological Research, 18/c. Bem square, 4026 Debrecen, Hungary; 2 Wetland Ecology Research Group, Danube Research Institute, Centre for Ecological Research, 18/c. Bem square, 4026 Debrecen, Hungary; 3 Department of Hydrobiology, University of Debrecen, P.O. Box 57, 4010 Debrecen, Hungary; 4 Institute of Ecology and Botany, Centre for Ecological Research, 2-4. Alkotmány Str., 2163 Vácrátót, Hungary

**Keywords:** Ecological modelling, Community ecology

## Abstract

Environmental filtering and limiting similarity are those locally acting processes that influence community structure. These mechanisms acting on the traits of species result in trait convergence or divergence within the communities. The role of these processes might change along environmental gradients, and it has been conceptualised in the stress-dominance hypothesis, which predicts that the relative importance of environmental filtering increases and competition decreases with increasing environmental stress. Analysing trait convergence and divergence in lake phytoplankton assemblages, we studied how the concepts of ‘limiting similarity’ versus ‘environmental filtering’ can be applied to these microscopic aquatic communities, and how they support or contradict the stress-dominance hypothesis. Using a null model approach, we investigated the divergence and convergence of phytoplankton traits along environmental gradients represented by canonical axes of an RDA. We used Rao’s quadratic entropy as a measure of functional diversity and calculated effect size (ES) values for each sample. Negative ES values refer to trait convergence, i.e., to the higher probability of the environmental filtering in community assembly, while positive values indicate trait divergence, stressing the importance of limiting similarity (niche partitioning), that is, the competition between the phytoplankters. Our results revealed that limiting similarity and environmental filtering may operate simultaneously in phytoplankton communities, but these assembly mechanisms influenced the distribution of phytoplankton traits differently, and the effects show considerable changes along with the studied scales. Studying the changes of ES values along with the various scales, our results partly supported the stress-dominance hypothesis, which predicts that the relative importance of environmental filtering increases and competition decreases with increasing environmental stress.

## Introduction

One of the main aims of community ecology is to find general rules of species coexistence. These rules help to understand how communities can behave under various environmental scenarios^1^. To accomplish this aim we must study the processes that shape species composition and abundances. The coexistence of species in a given site is usually controlled by abiotic and biotic filters, which admit or exclude species from an available pool. Recently, there is a growing consensus that these filters operate on the traits of species, rather than on species themselves^2,3^. Generally, two distinct non-random processes of species sorting exist: habitat filtering and limiting similarity. These non-random processes are thought to shape the mean, spread, and spacing of functional trait values differently within communities. On one hand, a specific trait combination will promote species (or specimen) success in a given environment. In this way, the environment filters out species (or individuals) that do not have the proper traits or trait combinations^4^. Such filtering leads to a converged trait distribution among the coexisting species within the communities (i.e. species become more similar than expected under random assembly^5,6^, albeit detection of this process depends largely on the studied traits and trait metrics. On the other hand, the ‘limiting similarity’ (or niche differentiation) concept^7,8^ states that competition for resources results in trait divergence, which encourages a stable coexistence between community members^9^. In other words, when two species show differences in their niches, they tend to compete less strongly. Such contests between species lead to divergent trait distribution (i.e., co-occurring species become more different in traits than expected by random selection from the species pool) within the communities. Several tools were developed and applied in the ecology to study the importance of assembly rules in recent years. These approaches, such as guild proportionality and limiting similarity approaches, have been reviewed by Götzenberger et al.^1^.

The recognition that selection acts on traits rather than species triggered various functional approaches in phytoplankton ecology^10–13^. However, the regulative power of competitive processes among the resident taxa and the role of environmental filtering on phytoplankton assemblages have not yet been studied by trait-based approaches.

Lakes’ phytoplankton is a highly diverse assemblage both in terms of species and in terms of its trait compositions^14^. The trait-based approach offers a tool for exploring the mechanisms that generate this high diversity and compositional variation. On the other hand, phytoplankton communities have properties that make them ideal for testing general hypotheses using trait-based approaches. In his conceptual synthesis, Vellend^15^ outlined four basic processes that determine the pattern of local communities: dispersal, drift (i.e. demographic stochasticity), selection and speciation. Although dispersal ability of algae is high^16^, recruitment of new species in local mature systems is exceptional rather than a common phenomenon^17^. Growth and extinction in small populations are subject to the effect of demographic stochasticity^18,19^. However, algal individuals in aquatic systems are present in astronomical numbers, therefore demographic stochasticity plays a negligible role in the community assembly^20^. Since speciation acts on a longer time-scale, we focus on the mechanisms related to the fourth process: selection, which includes habitat filtering and competition. Since the competition and the environmental filters result in different trends in the trait distributions, their potential role can be estimated by the trait composition of the local assemblages. Our aim was to investigate the impact of relevant physical and chemical properties of water on trait convergence or divergence in lakes’ phytoplankton and to study how the assembly rules vary in the growing season. Lakes’ phytoplankton assemblages undergo abrupt changes in the growing season, and occasionally their succession terminates in steady-state assemblages, which are dominated only by 1–3 species^21^. These characteristics make shallow lakes convenient objects to study how these transitions are followed by trait convergence or divergence.

The theory that relates trait convergence and divergence explicitly to environmental gradients is the stress-dominance hypothesis^22,23^, which predicts that in the harsher environment environmental filtering plays a major role and leads to trait convergence. In contrast, in benign ecological conditions, limiting similarity is the most decisive assembly rule resulting in trait divergence within the assemblages. Several studies on plant communities have been published to date in favour of this hypothesis, showing that nutrients and water are key determinants for plants, and their availability determines which assembly rule controls community composition^24–29^. Analyses on the role of environmental filtering or limiting similarity on animal communities also supported the stress dominance hypothesis demonstrating the importance of temperature on the trait composition of fish assemblages^30^, or aridity and other climatic factors on desert bat communities^31^. It has also been demonstrated that the traits reflect differently to the constraints of the habitats, and thus, there are competition-related and environmental filtering-related traits^32,33^.

In the context of the above-mentioned issues, we addressed the following research questions:

Can trait convergence or divergence be observed in lakes phytoplankton, and do they display changes along the environmental gradients?

Do the results support the stress-dominance hypothesis^23^, that is, trait convergence, and thus, the role of environmental filtering increases in harsher (less productive or light limited) environments, while limiting similarity and trait divergence are more important in benign (more productive or well-illuminated) ecological conditions?

## Results

Altogether, we recorded 877 algal taxa in the 283 samples. The main taxonomic groups with the number of occurring species are shown in Supplementary Table S1, (the whole taxa list is in Supplementary Table S2). Chlorococcalean green algae, diatoms and cyanobacteria were the most species rich groups, which are characteristic in lakes phytoplankton. The values of nutrients and proxies of phytoplankton biomass varied in the ultra oligotrophic – hypertrophic range, which enables us to study the trait distributions in a sufficiently large scale.

### Trait community weighted means—background variable relationships

**Figure 1:**
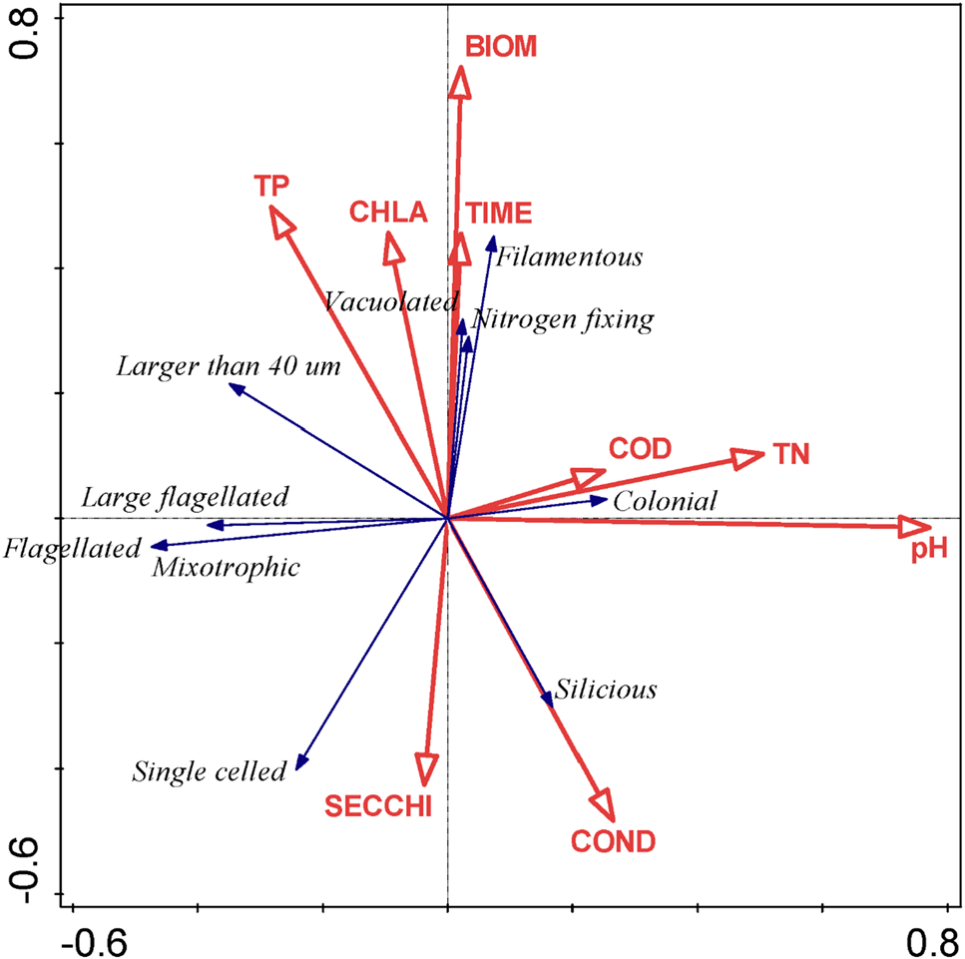
RDA biplots displaying the importance of physical and chemical characteristics of lakes on the relative abundance of the various algal traits. Red arrows are environmental variables, black arrows are traits. *Depth* mean depth of the water bodies (m); *area* area of the water bodies (m^2^), *Cond* electrical conductivity (μS cm^−1^); *TP* total phosphorus (μg L^−1^), *TN* total nitrogen (μg L^−1^), *CHLA* chlorophyll-*a* (μg L^−1^), *Secchi* Secchi transparency (m), *COD* chemical oxygen demand (mg L^−1^), *Time* number of weeks in the date of sampling Functional groups of algae are represented as circles and codes.

The results of RDA (shown in Fig. 1) indicate that nutrients and the phytoplankton biomass were the most important factors determining trait distributions in phytoplankton assemblages. The first canonical axis (eigenvalue: 0.1019, Table 1) associated well with pH and Total Nitrogen. The second canonical axis (eigenvalue: 0.0536) correlates with TP and proxies of phytoplankton biomass (Chl-*a* and Biomass) and Secchi transparency; therefore this axis was considered later as a stress gradient to evaluate the usefulness of the stress-dominance hypothesis as a possible rule in phytoplankton assembly.

The trait “colonial” positively, while mixotrophic, flagellated negatively correlated with the first canonical axis. The traits that are characteristic for bloom-forming cyanobacteria (elongated, vacuolated, nitrogen-fixing) positively associated with the second canonical axis, (details of the relationships are shown in Table 2, Supplementary Figure S1). In contrast, the siliceous and single celled traits negatively correlated with this axis.

**Table 1:** Summary table of RDA results total variation is 594.62792, explanatory variables account for 18.0% (adjusted explained variation is 12.4%).

Statistic	Axis 1	Axis 2	Axis 3	Axis 4
Eigenvalues	0.1019	0.0536	0.0113	0.0061
Explained variation (cumulative)	10.19	15.55	16.68	17.3
Pseudo-canonical correlation	0.4806	0.5634	0.3272	0.2433
Explained fitted variation (cumulative)	56.71	86.55	92.84	96.25
**Permutation test results**				
On first axis	Pseudo-F = 15.0, P = 0.002			
On all axes	Pseudo-F = 3.2, P = 0.002

**Table 2:** Summary table of the ES value distributions. Significant results are written in bold.

		Traits									
		Flagellated	Size (larger > 40 µm)	Colonial	Single celled	Filamentous	Mixotrophic	Siliceous	Nitrogen fixing	Vacuolated	Large flagellated
Distribution of the trait in the whole dataset (%)		26%	39%	28%	61%	11%	26%	20%	3%	5%	13%
Distribution of the ES values compared to the random distributions	Mean (ES)	**0.194**	**− 0.268**	**− 0.191**	**− 0.266**	**− **0.121	**0.194**	**− **0.101	**0.513**	**0.257**	**0.368**
	Level of significance	**0.003**	**0.000**	**0.010**	**0.000**	0.145	**0.004**	0.124	**0.000**	**0.001**	**0.000**
Slope of the linear regression model	Time	0.002	0.002	**− 0.022**	0.009	**0.061**	0.001	**− **0.018	**0.028**	**0.036**	**− **0.020
	TP	**− **0.085	0.029	**− **0.111	0.014	**0.769**	− 0.099	− 0.180	**0.381**	**0.441**	0.049
	TN	− 0.343	0.330	**0.705**	0.669	**0.782**	− 0.334	0.433	0.192	0.155	**−1.022**
	Biomass	− 0.110	− 0.025	0.030	0.210	**0.634**	− 0.106	**− 0.245**	**0.497**	**0.555**	−0.161
	Chlorophyll-*a*	− 0.125	− 0.132	− 0.025	0.220	**1.023**	− 0.131	− 0.248	**0.831**	**0.934**	−0.167
	COD	− 0.072	− 0.075	0.619	0.533	**1.290**	− 0.087	− 0.187	**1.146**	**1.190**	−0.058
	Secchi	0.199	0.209	0.039	− 0.350	**− 1.624**	0.208	0.394	**− 1.318**	**− 1.483**	0.266
	pH	0.233	0.091	**0.548**	**0.485**	**0.636**	0.245	0.228	**0.711**	**0.730**	−0.238
	Conductivity	−0.469	−0.183	**0.898**	0.256	−0.000	−0.497	0.183	−0.150	−0.222	−0.301
	RDA Axis 1	− 0.053	**− 0.140**	**0.109**	**0.107**	− 0.042	− 0.051	0.035	− 0.016	− 0.012	**−0.196**
	RDA Axis 2	− 0.014	0.015	0.013	0.035	**0.136**	− 0.020	**− 0.101**	**0.107**	**0.112**	−0.020
Significance of the linear regression modell	Time	0.780	0.804	**0.018**	0.352	**0.000**	0.916	0.031	**0.002**	**0.000**	0.044
	TP	0.442	0.813	0.379	0.917	**0.000**	0.372	0.115	**0.002**	**0.001**	0.716
	TN	0.130	0.219	**0.003**	0.011	**0.005**	0.142	0.065	0.350	0.490	**0.000**
	Biomass	0.206	0.796	0.766	0.040	**0.000**	0.223	**0.007**	**0.000**	**0.000**	0.129
	Chlorophyll-*a*	0.236	0.218	0.833	0.062	**0.000**	0.218	0.018	**0.000**	**0.000**	0.191
	COD	0.748	0.762	0.017	0.044	**0.000**	0.700	0.429	**0.000**	**0.000**	0.830
	Secchi	0.236	0.218	0.833	0.062	**0.000**	0.218	0.018	**0.000**	**0.000**	0.191
	pH	0.162	0.622	**0.003**	**0.014**	**0.001**	0.143	0.180	**0.000**	**0.000**	0.242
	Conductivity	0.093	0.551	**0.004**	0.438	0.079	0.076	0.524	0.616	0.496	0.377
	RDA Axis 1	0.105	**0.000**	**0.003**	**0.003**	0.313	0.117	0.286	0.666	0.757	**0.000**
	RDA Axis 2	0.624	0.617	0.682	0.278	**0.000**	0.500	**0.000**	**0.001**	**0.001**	0.575

The relationship between the environment and trait CWMs was also investigated at the level of variables. Altogether ninety environmental variable/Trait CWM models were evaluated (Table 2) and displayed (Supplementary Fig. 2–10). Significant departures from the NULL model (P < 0.001) were found in 36 occasions. Since both canonical axes correlated well with the properties of water considered relevant for the assembly of phytoplankton, we assessed CWM and ES values along these axes.

**Figure 2:**
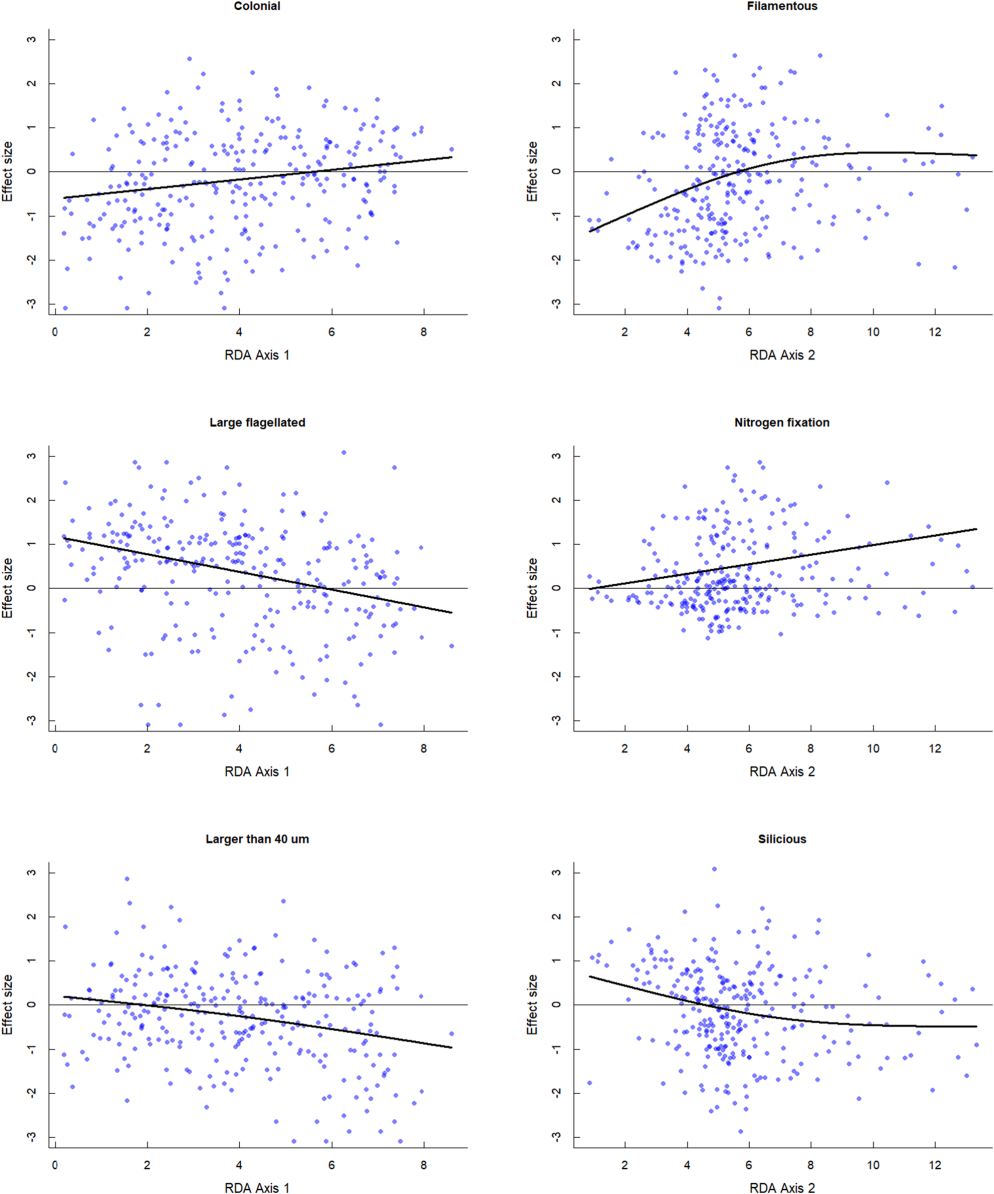
Effect sizes (ES) of selected functional traits along the first two canonical axes of the RDA. Dotted lines indicate the position of ES = 0. Positive values indicate divergence, negative ones convergence of traits. Each dot represents a sample on the gradient. Black lines indicate trends based on GAM.

### Distribution of the ES values in comparison with the null model

Results of the t-tests indicated that except for the filamentous and siliceous traits ES values of each trait showed non-random distribution (Table 2). In the case of size, colonial, single-celled, and nitrogen-fixing traits, the means of ES values appeared to be significantly lower than zero referring to higher probability of trait convergence. ES values significantly higher than zero characterised the distribution of flagellated, mixotrophic, vacuolated traits and in the case of large flagellated trait complex, indicating a higher probability of a trait divergence and thus larger role of competitive processes in the community assembly.

### Distribution of the ES values along the canonical axes

The effect size (ES) values represented various distribution patterns along the first canonical axis (Supplementary Figure S1). Values calculated for the traits coloniality were those that showed slightly increasing trends along the first axis. An opposing pattern characterised the changes of the ES values in the case of the size trait (algal units larger than 40 µm) and the “large flagellated” trait complex (Fig. 2). Effect size (ES) values of the other traits have not shown any trends (SupplementaryFigure S1).

Along the second canonical axis, we observed increasing trends in ES values of vacuolated filamentous and nitrogen fixing traits. An opposing, slightly decreasing trend was displayed by the siliceous trait (Fig. 2, Table 2). Characteristic trends in the ES values of other traits could not be observed (Supplementary Figure S1). Since the GAM did not differ from a simple linear regression, for simplicity, we reported the latter.

Changes of ES values with the measured variables were also studied. Since the canonical axes of the RDA correlated well with the measured properties of waters, the results showed remarkable similarities to those we experienced during the study of ES values/canonical axes relationships. Results are shown in Supplementary Figure S2–10.

## Discussions

### Trait distributions along the environmental gradients

The results of this study demonstrated that the environmental properties of water determine the distribution of several algal traits, resulting in trait convergence or divergence in the phytoplankton assemblages. Nutrient content of the studied lakes covered the whole trophic spectrum (from oligotrophy to hypertrophy) both in the case of phosphorus and nitrogen forms. The different roles of these two nutrients in phytoplankton assembly were well represented by the results of the RDA. The first canonical axis correlated with the TN, while the second one with the TP and other biomass-related variables (i.e. chlorophyll-a and phytoplankton biomass). Although the growth of phytoplankton in most freshwaters is limited by phosphorus, nitrogen limitation frequently occurs in lakes with TP > 30 µg l^−1^ ^34^. In our database, TN/TP ratios varied between 1 and 132 (not shown here) referring to the possible role of N limitation even in eutrophic environments. However, the fact that TP, the biomass related variables and Secchi transparency associated with the second axis of the RDA refers to the importance of phosphorus in controlling phytoplankton biomass. Flagellated, mixotrophic and single celled traits associated with the lower values of RDA axes. In our database taxa sharing these traits were unicellular flagellated organisms such as chrysophytes (*Chrysochromulina* spp.) or cryptophytes (*Rhodomonas* cf. *nannoplanctonica*). These taxa have high metabolic activity and prefer oligotrophic environments^35^. The traits characteristic for bloom forming, heterocytic cyanobacteria (elongated, vacuolated, nitrogen fixing) associated with the large values of the second RDA axis, referring to eutrophic environments, with occasional nitrogen limitation.

The time (number of weeks when samples were collected) also correlated with this axis, which is in accordance with our present knowledge on phytoplankton succession, that is, biomass maxima are expected to occur in late summer periods^36^.

### Trait convergence or divergence in lakes phytoplankton

The theory of environmental filtering versus limiting similarity is well established in the literature and provides a framework of how communities are assembled^25,37^. In our study, ES values of most traits significantly differed from 0, which clearly indicated both convergence and divergence of traits and the importance of the related assembly mechanisms. In line with the reasoning above, in the case of traits where ES values were significantly lower than zero the results strongly supported the higher probability of environmental filtering.

The ES values showed different patterns along the two canonical axes. Along the first axis, which was primarily determined by the TN, ES values of only the size trait and the large flagellated trait complex showed remarkable trends. That the ES values of the nitrogen-fixing trait did not show any changes along this axis, could be accounted for by that the nitrogen limitation depends on the TP/TN ratio, not on the absolute quantity of nitrogen.

In this study, the most remarkable trends characterised those traits (nitrogen fixing and vacuolated; Table 2), that are associated with elongated bloom-forming cyanobacteria and considered to give a real competitive advantage to phytoplankters that live in nitrogen and light deficient environments^38^. Since from the midsummer period (~ week 30) ES values of these traits showed remarkable, significant increase with the trophic state-related variables (TP, Biomass and Chl-*a*, 2nd RDA axis), it is reasonable to suppose that resource competitions are responsible for this trend. Thus these traits can be considered as competition-related traits.

In several cases, ES values of functional traits showed random distribution along the gradients (Table 2). However, this does not necessarily mean that these traits are neutral. Environmental filtering and limiting similarity might act simultaneously and can compensate each other; therefore, the values of test statistics support the null hypothesis.

Since we applied exclusively binary traits, distance calculations between species can result in only two distance values: 0 and 1. Our results clearly highlighted that even despite this simplification, in the cases of very competitive traits (nitrogen fixation or vacuolated) the applied method could successfully indicate trait divergence, that is, the potential role of limiting similarity as a leading assembly rule in phytoplankton assemblages. However, there were several traits, e.g., mixotrophy or flagellated, where this simplification did not seem to be successful. Several algal groups have the capability of consuming organic particles, but the rate of the consumption and its importance on the overall nutrition of cells can vary considerably among the major groups^39,40^. The fact that mixotrophic traits did not display any relationship with the canonical axes indicates that merging all algae that are capable of mixotrophy into one “mixotrophic group” is an oversimplification of the phenomenon. The same statements can be made for the flagellated trait. Within this trait, the algae considerably differ from each other in terms of their size, nutrient preferences and tolerances. Therefore, the assignment of each flagellated taxa into a single group might occasionally lead to improper conclusions. However, during the application of the trait complex “large flagellated” we experienced a decreasing trend in the ES values along the first canonical axis, which suggests that finer resolution of the groups created by their single trait characteristics may contribute to the understanding the underlying processes.

It is important to note that the applied method has some shortcomings when binary traits are studied. If distribution of a binary trait in the dataset approximates the 50%, trait divergence cannot be grasped. The same can be said for the trait convergence, if the distribution of a trait is highly unequal in the dataset (trait distributions in the dataset are shown in Table 2).

### Results in the context of ecological theories

Convergence and divergence of traits can be interpreted in the context of the stress-dominance hypothesis^23^. The stress-dominance hypothesis predicts that along an increasing stress gradient, the importance of environmental filtering increases while competition decreases. Following Grime’s definition^41^, the stress can be defined as external constraints limiting the rate of biomass production. Since the availability of nutrients and light controls primarily the production of algae, at the lower end of these gradients nutrient limitation might occasionally occur, while in the upper end them, due to the large phytoplankton biomass, reduced availability of light may potentially limit algal production. This logic was supported by the results of RDA, where the biomass-related variables (biomass and Chl-*a*) associated with TP, but they showed an opposite direction to the Secchi transparency (2nd RDA axis).

Supposing that traits respond to nutrient and light limitation similarly, a humpback relationship could be expected^42^, but none of the traits’ ES values displayed this kind of pattern. The decreasing or increasing trends we experienced might occasionally occur when narrow ranges of independent variables are studied. However, in our case, the lakes involved in this study covered the whole trophic spectrum, from ultra oligotrophic to hypertrophic. The possible explanation is that the traits applied in this study respond differently to the nutrient and light limitations, which phenomenon differentiates algae from the higher plants.

The increasing trends observed in the ES values of filamentous, vacuolated and nitrogen-fixing traits are in line with the stress-dominance hypothesis. These traits are characteristic to bloom-forming cyanobacteria, which prevail in nutrient-rich environment. Their elongated form makes them very good light harvesters^43^, vacuoles helps them in buoyancy regulation finding the optimal position in the water column, while nitrogen fixation enables the taxa to flourish in nitrogen-deficient environments. Since higher ES values appear when CWM values are approaching the 0.5 value, the results imply that besides these traits there can be several other traits (small cell size, mixotrophy) by which planktic organisms can cope with the unfavourable light conditions, or occasional nitrogen limitation, which might develop even in eutrophic environment^34^.

We observed an opposite trend along the 1st canonical axis (correlated with TN) in the case of the large flagellated trait complex. The higher probability in trait divergence occurred in lower TN concentrations, while in higher concentration range trait convergence is more probable. This pattern contradicts to the stress dominance hypothesis. The CWM values of this trait showed a decreasing tendency, indicating that taxa having these traits (mostly dinoflagellates) are important elements of the phytoplankton in oligotrophic lakes, play only a minor role in highly eutrophic environments.

We must note that in late summer after long-lasting calm periods bloom-forming cyanobacteria outcompete other elements of the phytoplankton, which might lead to monodominance of the filamentous, vacuolated and nitrogen fixing traits in the phytoplankton^44^, and thus, competition ends in a trait convergence. Thus, we note that the length of periods favourable for competitive processes in the phytoplankton cannot be neglected when the role of habitat filtering or niche differentiation is studied.

## Conclusions

The null model approach demonstrated that random processes could not be neglected as assembly mechanisms driving compositional changes in shallow lakes’ phytoplankton. However, we can conclude that depending on the selected traits, environmental filtering and limiting similarity can also play a crucial role. Our results proved that filamentous, vacuolated and nitrogen fixing are those so-called competition-related traits, in the case of which niche partitioning can be safely demonstrated. The observed trait divergence in the high trophic range indicates, that extreme eutrophication does not necessarily coincide with monodominance of bloom forming cyanobacteria because the species pool of eutrophic lakes’ phytoplankton contains elements with other types of competitive functional traits that enable them to flourish in this kind of environments. Contrary to studies on terrestrial primary producer communities (e.g.^25,45^), our results only partially supported the stress-dominance hypothesis.

## Materials

### Studied lakes

We used long-term phytoplankton monitoring data for Hungarian (Pannonian ecoregion), Romanian (Pannonian ecoregion) and Croatian (Dinaric region) standing waters (Supplementary Table S3). The dataset contains data for 283 phytoplankton samples collected from 37 water bodies in May–October period between 1992 and 2018. The climate in the Pannonian region is humid continental with warm summers (summer mean temperature is 21–23 °C), and with an average annual precipitation of 450–600 mm^46^. The climate in the Dinaric region is transitional continental with similar temperature but slightly higher precipitation^47^. Most of the lakes in the Pannonian region are polimictic oxbows and shallow ponds developed in deflation pools^48,49^. The selected water bodies of the Dinaric region are deep reservoirs of low trophic status. Since the dataset includes species data with relative and absolute biomass abundances, this provides an opportunity to study trait distribution within the samples.

### Sampling and sample processing

Samples were taken by tube sampler from the trophic layer (2.5 × Secchi depth) at the deepest parts of the lakes. In the case of water bodies where the maximum depth (Zmax) was less than 2 m, the whole water column has been sampled. Conductivity (µS cm^−1^) and pH was measured by a portable-multiparameter digital meter (HQ30d) in the field (Supplementary Table S4). Phytoplankton samples (0.5 L) were fixed with Lugol’s solution. Qualitative and quantitative analyses of phytoplankton were performed using inverted microscopes according to Utermöhl’s method^50^. The samples were allowed to settle in 1, 5 or 10 cm^3^ counting chambers. In each sample at least 400 units (cells, filaments or colonies) were counted along transects at 400-fold magnifications. The small-celled taxa (chlorelloid green algae or *Romeria* spp.) which occasionally occurred in high numbers in the samples were counted in each field at 400-fold magnification. Area of the whole counting chamber was investigated at 100-fold magnification to determine the relative abundance of rare, large-sized taxa. All specimens were identified to species level. In oligotrophic lakes where centric diatoms occasionally attained higher relative abundance, samples were pretreated and investigated under 1000-fold magnification.

Since the trophic status of waters has a pronounced impact on phytoplankton composition and diversity^51^ five trophic state related variables were used as gradients: phytoplankton biomass, chlorophyll-*a*, total phosphorus (TP), total nitrogen (TN) and chemical oxygen demand (COD). Algae were identified to species level. Phytoplankton biomass was calculated by considering algal biovolume performed according to^52^ and converted to wet weight (mg/L) assuming a specific gravity of 1. Sestonic chlorophyll-*a* concentration was measured spectrophotometrically and corrected for phaeophytin^53^. Background variables (TP, TN and COD) were measured according to the national standards. Total phosphorus concentration was determined as soluble reactive phosphorus after H_2_SO_4_ digestion using the acid molybdate method^54^. To determine total nitrogen (TN) the method using oxidative digestion with peroxodisulfate was applied^55^. The measurement of chemical oxygen demand (COD) was based on the dichromate reaction method^56^. The values of measured parameters are shown in Supplementary Table S4.

We aimed to study within-year changes in the strength of trait convergence and divergence. Therefore, we used the dates of samplings measured by week within years as explanatory variables. Since the samples were taken in May–October periods in each year, its scale ranged from 19 to 44th weeks.

### Functional traits

Nine functional traits were studied. Eight of them are binary variables: flagellated, colonial, single celled, filamentous, mixotrophic, siliceous (mostly, but not exclusively diatoms), nitrogen fixing and vacuolated (species with gas vacuoles). Size was the only continuous trait, but we binarized that using linear dimensions, larger than 40 µm as a threshold. These traits are based on those visible morphological features of algae that refer to special biochemical, physiological or physical adaptations, and basically influence their functioning and ecological roles in the planktic assemblages^12^. Besides the single traits, we also applied a trait combination namely the “large flagellated” (Supplementary Table S2).

### Statistical analyses

**Figure 3:**
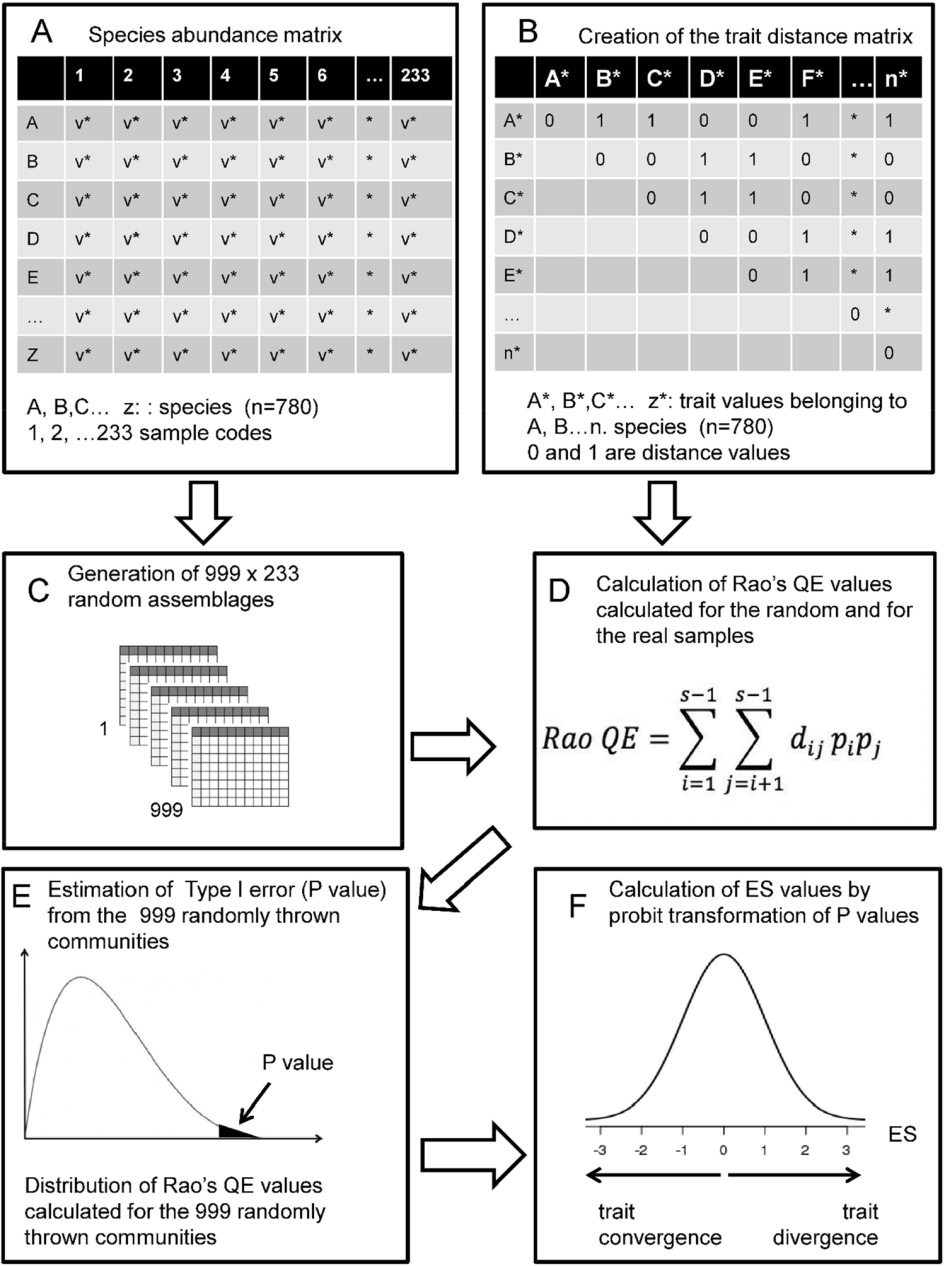
Main steps of the statistical approach.

**Figure 4:**
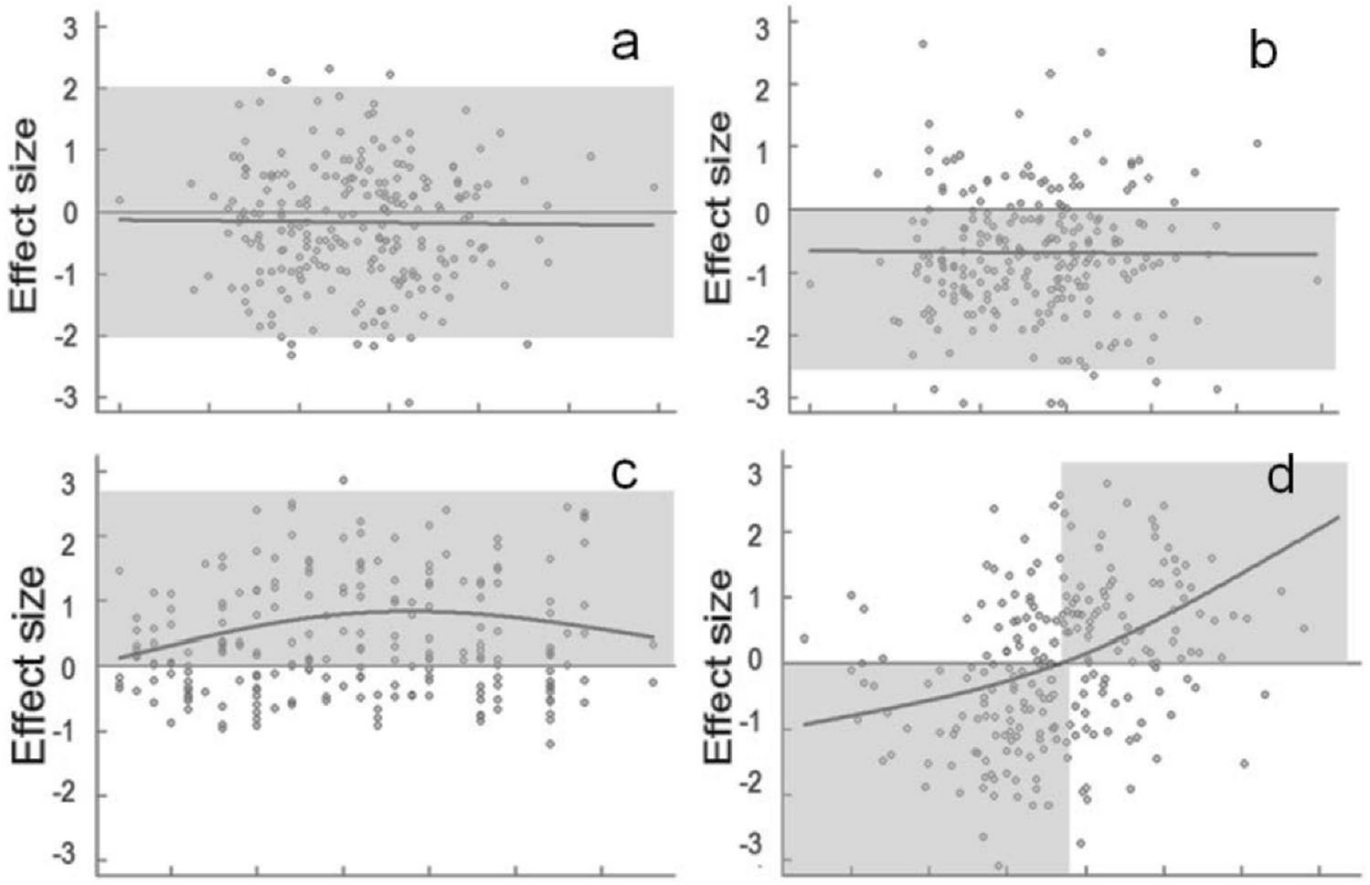
Possible outcomes of the analyses. Each dot represents a sample on the gradient/effect size plot. **(a)** Traits showing neither convergence nor divergence; **(b)** trait convergence, **(c)** trait divergence; **(d)** shift from trait convergence to divergence.

To study the convergence or divergence of traits we used a null model approach applied by^25^. Null model approaches are the most frequently used tools studying the assembly rules because these are suitable for identification of non-random components in community composition^1^. Details of the procedure are shown in Fig. 3. The essence of the approach is that trait divergence or convergence is characterised by a test statistic and this test statistic was calculated for 999 random samples created from the species pool. The test statistic was also calculated for the real samples and proportions of random communities where test statistic is more extreme than in the field sample p-values were calculated. We used probit transformed p-values as ‘effect size values’ (ES), which indicate the strength of trait divergence or convergence^57^. Positive ES values indicate that competition is the leading assembly rule, while negative ones refer to the leading role of environmental filtering (Fig. 4). Significant differences in ES values from zero for the whole dataset were tested by the Student’s t-test.

Since the dominant assembly rule may change seasonally or along environmental gradients, effect size values were plotted against various gradients. To reveal the direction of changes in ES values we applied the generalized additive model (GAM).

The whole procedure outlined above was repeated for each trait separately. The consecutive steps of the procedure are shown in Fig. 3.

This approach is a flexible framework where appropriate test statistics and randomization algorithms have to be selected in each study according to its purposes. We used Rao’s quadratic entropy^58^ as test statistic:

$$Rao\, QE= \sum_{i=1}^{s-1}\sum_{J=i+1}^{s-1}{d}_{ij }{p}_{i} {p}_{j}$$

where: Rao QE is the value of Rao’s quadratic entropy, *d*_*ij*_ is the trait-based distance between species *i* and *j*, *p*_*i*_ and *p*_*j*_ is the relative biomass abundance of species *i* and *j*.

There are lot of possible distance measures for binary data^59^. They differ when several binary variables are considered, but all results in the same value when only one variable (trait) is considered: zero is the distance if both species have or lack the trait, and the distance is one if only one of them has the given trait. We applied T1 randomization^60^ that means reshuffling trait values in the whole dataset while the matrix of species composition remained untouched. This combination of test statistic and randomization algorithm allows detecting both trait convergence due to environmental filtering, and trait divergence due to limiting similarity^60,61^. Illustration of the possible outcomes of the analyses are shown in Fig. 4.

To characterise the trait distribution along the gradients, we calculated community weighted mean (CWM) values^62^ for each sample. These values are the mean trait values weighted by the relative abundance of species. Since in this study we used binary traits, CWM is equal with the relative abundance of the trait in the sample. We applied GAM regression to derive the environmental gradient/CWM relationships.

We used redundancy analysis (RDA) to reveal the relationship between physical and chemical properties of water and the trait composition (i.e. CWMs of traits) of the planktic assemblages. To avoid nonlinearity and the impact of outliers, explanatory variables were log-transformed. We used the first two canonical axes of the RDA to study the patterns of trait distributions (CWM) and the trends in the traits’ ES values. Collinearity was tested using the variance inflation factor (VIF).

All statistical analyses and graphs were performed under R environment, with the relevant packages (vegan, ggplot2, dplyr and ade4)^63–65^.

### Evaluation of the community weighted mean (CWM)—effect size (ES) value relationship

Since the calculation of both CWM and ES values have been based on the relative abundance of binary traits, there were some regularities in the variation of these values. Maximum of the Rao Q value is obtained when biomass abundance of a given trait is 50%, therefore, an increase in ES can be expected when the CWM of the trait approaches 0.5 and starts from relative abundance range of 0 or 1. Exactly the same logic is standing behind the relationship between ES and CWM when the former shows U-shaped curve. In this case, CWM can occasionally show both bell-shaped and U-shaped distributions. It is feasible if CWMs are approaching the 0 or 1 abundance values in the middle range of the background variables.

## Supplementary information

Supplementary Table S1.

Supplementary Table S2.

Supplementary Table S3.

Supplementary Table S4.

Supplementary Figure S1.

Supplementary Figures S2–S10.

## Data Availability

The data that support the findings of this study are available from the corresponding author, upon reasonable request.
