# Supplementary material for: Trait convergence and trait divergence in lake phytoplankton reflect community assembly rules
Source: Sci Rep. 2020 Nov 11;10:19599. doi: 10.1038/s41598-020-76645-7 (PMC7658209; doi:10.1038/s41598-020-76645-7)
Supplement: Supplementary file 1 — Supplementary Table S1. [file 41598_2020_76645_MOESM1_ESM.docx]

Electronic Supplementary Material : Supplementary Table S1. Common taxa

**Trait convergence and trait divergence in lake phytoplankton reflect community assembly rules**

^1,2^Gábor Borics, ^2^Viktória B-Béres, ^3^István Bácsi, ^1^Balázs A. Lukács, ^1^E T-Krasznai, ^2,4^Zoltán Botta-Dukát, ^1,2^Gábor Várbíró^*^

^1^MTA Centre for Ecological Research, Danube Research Institute, Department of Tisza Research, 18/c. Bem square, 4026 Debrecen, Hungary

^2^MTA Centre for Ecological Research, GINOP Sustainable Ecosystems Group, 3. Klebelsberg Kuno str., H-8237 Tihany, Hungary

^3^University of Debrecen, Department of Hydrobiology, P.O. Box 57, H-4010 Debrecen, Hungary

^4^MTA Centre for Ecological Research, Institute of Ecology and Botany, 2-4. Alkotmány str., H-2163 Vácrátót, Hungary

| **Taxonomic group** | **Number of species** |
| --- | --- |
| [Sphaeropleales](http://www.algaebase.org/browse/taxonomy/?id=90759) | 225 |
| Bacillariophyta | 149 |
| Cyanobacteria | 114 |
| [Euglenophyceae](http://www.algaebase.org/browse/taxonomy/?id=4340) | 89 |
| Desmidiales | 52 |
| Chrysophyceae | 42 |
| [Volvocaceae](http://www.algaebase.org/browse/taxonomy/?id=4948) | 34 |
| [Dinophyceae](http://www.algaebase.org/browse/taxonomy/?id=4346) | 20 |
| Xanthophyceae | 19 |
| Cryptophyta | 17 |
| Klebsormidiales | 7 |
| Ulotrichales | 7 |
| Vacuolariaceae | 2 |
| Zygnematales | 2 |
| Eustigmatophyceae | 1 |
| [Prasinophytina](http://www.algaebase.org/browse/taxonomy/?id=142049) | 1 |
| Trebouxiophyceae | 1 |
| sum | 782 |
