# Supplementary material for: Trait convergence and trait divergence in lake phytoplankton reflect community assembly rules
Source: Sci Rep. 2020 Nov 11;10:19599. doi: 10.1038/s41598-020-76645-7 (PMC7658209; doi:10.1038/s41598-020-76645-7)
Supplement: Supplementary file 3 — Supplementary Table S3. [file 41598_2020_76645_MOESM3_ESM.docx]

Electronic Supplementary Material : Table 3. Chemical characteristics of lakes involved into the analyses (mean and minimum-maximum range)

**Trait convergence and trait divergence in lake phytoplankton reflect community assembly rules**

^1,2^Gábor Borics, ^2^Viktória B-Béres, ^3^István Bácsi, ^1^Balázs A. Lukács, ^1^E T-Krasznai, ^2,4^Zoltán Botta-Dukát, ^1,2^Gábor Várbíró^*^

^1^MTA Centre for Ecological Research, Danube Research Institute, Department of Tisza Research, 18/c. Bem square, 4026 Debrecen, Hungary

^2^MTA Centre for Ecological Research, GINOP Sustainable Ecosystems Group, 3. Klebelsberg Kuno str., H-8237 Tihany, Hungary

^3^University of Debrecen, Department of Hydrobiology, P.O. Box 57, H-4010 Debrecen, Hungary

^4^MTA Centre for Ecological Research, Institute of Ecology and Botany, 2-4. Alkotmány str., H-2163 Vácrátót, Hungary

| **Sampling location** | **No.** | **pH** | **Conductivity** | **TP** | **TN** | **COD)** | **Chlorophyll-a** | **Biomass** |
| --- | --- | --- | --- | --- | --- | --- | --- | --- |
|  |  |  | (µS cm^-1^) | (µgL^-1^) | (mgL^-1^) | (mgL^-1^) | (µgL^-1^) | (mgL^-1^) |
| Atkai-Holt Tisza, Algyő, Hungary | 5 | 8.33  (8.13 - 8.51) | 1051  (950 - 1270) | 292  (60 - 430) | 1.9  (1.510 - 2.20) | 30.9  (15.2 - 37.7) | 10.3  (5 - 25) | 1.13  (0.11 - 3.05) |
| Atkai-Holt Tisza, Szeged, Hungary | 4 | 8.29  (8.13 - 8.37) | 1057  (1030 - 1080) | 325  (160 - 480) | 2.58  (2.50 - 2.70) | 42.4  (35.6 - 53.6) | 9.7  (5.9 - 15) | 3.99  (0.3 - 9.25) |
| Egyeki Holt Tisza, Egyek ,Hungary | 18 | 7.63  (6.97 - 8.39) | 690  (577 - 827) | 485  (50 - 1860) | 3.26  (1.30 - 20.40) | 57.4  (3 - 88.2) | 42.8  (1.1 - 112) | 15.14  (0.14 - 60.77) |
| Egyek-Kócsi Tározó, Górés, Hungary | 15 | 7.7  (7.28 - 8) | 437  (366 - 506) | 251  (46.3 - 760) | 1.86  (1.30 - 5.90) | 36.6  (19.6 - 68.7) | 12.3  (0.5 - 31.6) | 5.25  (0.02 - 30.56) |
| Fancsika 1, Debrecen, Hungary | 5 | 8.79  (8.4 - 9.29) | 716  (616 - 826) | 407  (193 - 651) | 3.69  (3.03 - 4.60) | 134.0  (78 - 280) | 219.6  (134.5 - 440) | 123.08  (56.11 - 229.14) |
| Fancsika 2, Debrecen ,Hungary | 4 | 8.61  (8.3 - 9.15) | 494  (453 - 565) | 269  (226 - 300) | 2.21  (1.978 - 2.39) | 55.5  (34 - 70) | 89.0  (42.1 - 141.9) | 68.47  (35.4 - 144.76) |
| Félhalmi-holtág, Hungary | 3 | 8.1  (7.9 - 8.36) | 641  (599 - 670) | 109  (40 - 167) | 2.19  (1.064 - 2.90) | 89.4  (37.3 - 189.2) | 16.3  (7.2 - 27.7) | 9.51  (6.53 - 12.88) |
| Galaţui, Romania | 2 | 8.2  (8.1 - 8.3) | 521  (515 - 528) | 191  (125 - 258) | 2.91  (2.26 - 3.55) | 15.0  (15 - 15) | 27.7  (1 - 54.5) | 23.56  (3.11 - 44.01) |
| Holt-Szamos, Géberjén, Hungary | 9 | 8.16  (7.89 - 8.51) | 674  (611 - 772) | 468  (220 - 1170) | 2.73  (1.56 - 3.30) | 40.57  (6.7 - 66.9) | 28.7  (11.7 - 62.5) | 24.21  (4.14 - 93.94) |
| Holt-Szamos, Tunyogmatolcs, Hungary | 8 | 8.31  (7.81 - 8.67) | 611  (528 - 648) | 699  (242 - 1070) | 2.96  (2.30 - 4.20) | 36.0  (10 - 53) | 24.9  (11.1 - 38.4) | 19.84  (5.04 - 41.92) |
| Kakasszéki-tó, Székkutas, Hungary | 3 | 8.5  (8.32 - 8.65) | 2100  (1980 - 2300) | 373  (170 - 510) | 22.77  (3.70 - 60) | 112.0  (96 - 141) | 176.3  (88.8 - 280) | 26.05  (1.12 - 74.44) |
| Kati-tó, Debrecen, Hungary | 5 | 8.14  (7.78 - 8.6) | 569  (514 - 638) | 277  (220 - 310) | 1.69  (1.32 - 2.42) | 49.0  (30 - 69) | 103.7  (11.6 - 201.7) | 30.23  (3.52 - 65.7) |
| Lake Crniševo Croatia | 5 | 8.16  (7.9 - 8.3) | 384.6  (366 - 399) | 15  (9 - 20) | 0.71  (0.51 - 0.92) | 9.95  (9.61 - 10.33) | 1.44  (0.8 - 2.1) | 0.5  (0.29 - 0.77) |
| Lake Kozjak Croatia | 6 | 8.23  (8.1 - 8.3) | 412  (389 - 428) | 18.5  (12 - 25) | 0.72  (0.6 - 0.94) | 10.66  (10.09 - 11.44) | 4.4  (2.45 - 7.83) | 1.64  (0.3 - 2.98) |
| Lake Oćuša Croatia | 6 | 8.28  (8 - 8.4) | 428.67  (424 - 434) | 18.67  (13 - 25) | 0.31  (0.1 - 0.77) | 11.88  (10.99 - 12.93) | 1.8  (0.4 - 5.64) | 0.53  (0.21 - 1.23) |
| Lake Prošće  Croatia | 6 | 8.21  (8.1 - 8.3) | 548.5  (422 - 600) | 11.67  (1.5 - 36) | 0.42  (0.2 - 0.66) | 11.35  (9.85 - 12.87) | 3.5  (1.04 - 5.67) | 0.77  (0.34 - 1.71) |
| Lake Visovačko Croatia | 6 | 8.83  (8.3 - 9.3) | 2295  (1710 - 3480) | 30.83  (22 - 46) | 0.95  (0.77 - 1.07) | 75.01  (46.66 - 99.16) | 7.36  (2.69 - 15.43) | 5.71  (1.3 - 12.1) |
| Lake Vransko (Biograd)  Croatia | 10 | 8.4  (8 - 9.3) | 1647.58  (503 - 5300) | 24.33  (13 - 42) | 0.66  (0.24 - 1.02) | 43.56  (9.19 - 101.08) | 11.71  (2.61 - 60.9) | 4.4  (0.61 - 13.45) |
| Lake Vransko (Cres Island)  Croatia | 6 | 8.13  (8.04 - 8.24) | 2196.17  (587 - 2930) | 16.08  (1.5 - 45) | 0.55  (0.32 - 0.85) | 13.88  (11.15 - 17.33) | 3.17  (1.96 - 5.26) | 0.77  (0.39 - 1.1) |
| Madarász-tó, Mórahalom, Hungary | 5 | 8.55  (8.24 - 9.2) | 1340  (730 - 1980) | 410  (190 - 900) | 3.4  (3 - 3.68) | 115.6  (68.3 - 252) | 204.6  (36 - 790) | 30.93  (3.25 - 120.54) |
| Mézeshegyi tó, Debrecen, Hungary | 9 | 8.59  (7.4 - 9.45) | 505  (366 - 608) | 615  (283 - 1801) | 2.58  (1.17 - 4.35) | 128.3  (47 - 304) | 326.2  (18.4 - 1333.7) | 206.04  (0.72 - 640.46) |
| Nagybaracskai Holt-Duna, Dunafalva, Hungary | 3 | 7.59  (7.25 - 7.81) | 756  (585 - 880) | 170  (50 - 350) | 2.43  (1.80 - 2.90) | 29.6  (28.7 - 30.5) | 10.7  (1.2 - 20) | 0.51  (0.17 - 1.17) |
| Nagyréti - tározó, Hungary | 3 | 8.21  (8.11 - 8.31) | 692  (655 - 733) | 326  (297 - 350) | 16.33  (1.30 - 45) | 52.1  (48.5 - 54.2) | 37.4  (25.9 - 48.6) | 8.51  (4.66 - 11.56) |
| Rétközi-tó, Szabolcsveresmart, Hungary | 5 | 8.24  (8.04 - 8.85) | 320  (259.8 - 367) | 312  (126 - 493) | 3.22  (2.1 - 4) | 39.4  (29.9 - 51.7) | 64.2  (29.4 - 126.1) | 28.64  (8.82 - 66.84) |
| Serházzugi Holt-Tisza, Csongrád, Hungary | 7 | 8.39  (7.66 - 9.09) | 835.71  (795 - 880) | 744.29  (110 - 4200) | 2.82  (2.43 - 3.4) | 51.86  (35 - 88.5) | 90.4  (72 - 120) | 10.32  (0.44 - 23.3) |
| Snagov, Romania | 8 | 8.21  (7.9 - 8.6) | 524.14  (487 - 561) | 49.88  (10 - 130) | 1.69  (1.2 - 2.396) | 22.82  (18.88 - 28.8) | 15.0  (1.17 - 27.3) | 6.21  (0.91 - 18.68) |
| Szarvas-Békésszentandrási holtág, Hungary | 6 | 8.03  (7.9 - 8.3) | 427.5  (384 - 505) | 247.5  (140 - 380) | 2.14  (1.1 - 2.9) | 21.47  (14.9 - 32.2) | 42.93  (11.7 - 119.3) | 8.39  (1.34 - 14.42) |
| Szarvasi-holtág, Hungary | 2 | 8.21  (7.9 - 8.3) | 519  (519 - 519) | 377  (377 - 377) | 2.29  (1.88 - 2.69) | 26  (25 - 27) | 6.9  (5.5 - 8.3) | 0.17  (0.16 - 0.17) |
| Szelidi-tó, Dunapataj, Hungary | 8 | 9.01  (8.51 - 9.84) | 2191.67  (1990 - 2420) | 93.75  (50 - 210) | 2.62  (1.61 - 4.4) | 73.58  (49 - 145) | 23.85  (7.4 - 43) | 16.91  (0.95 - 62.06) |
| Tiszadobi Holt-Tisza, Darab Tisza, Hungary | 19 | 7.81  (7.28 - 8.14) | 257.76  (218 - 322) | 408.42  (33.3 - 2160) | 1.56  (1.16 - 2.2) | 19.89  (10.4 - 28.4) | 16.63  (1.4 - 114.6) | 10.75  (0.22 - 51.78) |
| Tiszadobi Holt-Tisza, Falu-Tisza, Hungary | 13 | 8.16  (7.84 - 8.51) | 332.42  (272 - 369) | 1132.31  (40 - 9450) | 1.5  (1.1 - 1.92) | 29.1  (20.9 - 42.8) | 18.39  (5.2 - 42) | 19.71  (3.91 - 54.79) |
| Tiszadobi Holt-Tisza, Felső Darab Tisza, Hungary | 11 | 7.78  (7.36 - 8.49) | 296.09  (246 - 365) | 449.09  (80 - 1120) | 1.6  (1.24 - 2.2) | 38.79  (13.4 - 80.2) | 68.35  (1.2 - 329.2) | 31.47  (0.01 - 95) |
| Tiszadobi Holt-Tisza, Malom-Tisza flooting, Hungary | 15 | 7.82  (7.47 - 8.22) | 302.6  (276 - 328) | 262.74  (42 - 1360) | 1.67  (1.07 - 2.3) | 30.92  (21.6 - 48.7) | 14.52  (2.3 - 31.5) | 21.69  (1.87 - 148.89) |
| Tiszadobi Holt-Tisza, Malom-Tisza open, Hungary | 5 | 8.14  (7.96 - 8.28) | 299.2  (293 - 305) | 58.8  (47 - 76) | 2.22  (1.6 - 3.1) | 31.28  (21.7 - 37.5) | 28.64  (11.2 - 83.7) | 10.09  (6.51 - 14.35) |
| Tiszadobi Holt-Tisza, Szűcs- Tisza, Hungary | 22 | 7.79  (7.3 - 8.75) | 344.88  (259.1 - 395) | 241.83  (46 - 2430) | 1.9  (1.22 - 5.3) | 37.57  (26.4 - 64.8) | 18.45  (4.7 - 60.4) | 12.38  (1.17 - 49.61) |
| Vadkerti-tó, Soltvadkert, Hungary | 7 | 8.68  (8.06 - 9.04) | 581.71  (492 - 685) | 112.86  (60 - 180) | 3.28  (2 - 6.2) | 64.79  (48.5 - 102) | 69.14  (16 - 160) | 25.37  (1.83 - 99.26) |
| Vidreéri halastavak, Felgyő, Hungary | 4 | 8.29  (7.81 - 8.61) | 1307.5  (1070 - 1520) | 450  (170 - 730) | 3.47  (2.3 - 5.48) | 110.03  (36.4 - 175) | 144.75  (30 - 340) | 15.04  (5.56 - 29.2) |
