# Supplementary material for: Trait convergence and trait divergence in lake phytoplankton reflect community assembly rules
Source: Sci Rep. 2020 Nov 11;10:19599. doi: 10.1038/s41598-020-76645-7 (PMC7658209; doi:10.1038/s41598-020-76645-7)
Supplement: Supplementary file 4 — Supplementary Table S4. [file 41598_2020_76645_MOESM4_ESM.docx]

Electronic Supplementary Material : Table S4. Characteristics of lakes involved into the analyses (mean and minimum-maximum range)

**Trait convergence and trait divergence in lake phytoplankton reflect community assembly rules**

^1,2^Gábor Borics, ^2^Viktória B-Béres, ^3^István Bácsi, ^1^Balázs A. Lukács, ^1^E T-Krasznai, ^2,4^Zoltán Botta-Dukát, ^1,2^Gábor Várbíró^*^

^1^MTA Centre for Ecological Research, Danube Research Institute, Department of Tisza Research, 18/c. Bem square, 4026 Debrecen, Hungary

^2^MTA Centre for Ecological Research, GINOP Sustainable Ecosystems Group, 3. Klebelsberg Kuno str., H-8237 Tihany, Hungary

^3^University of Debrecen, Department of Hydrobiology, P.O. Box 57, H-4010 Debrecen, Hungary

^4^MTA Centre for Ecological Research, Institute of Ecology and Botany, 2-4. Alkotmány str., H-2163 Vácrátót, Hungary

| **Sampling location** | **No.** | **Depth** | **Surface Area** | **WGS’84** | **WGS’84** |
| --- | --- | --- | --- | --- | --- |
|  |  | (m) | (km^2^) | North | East |
| Atkai-Holt Tisza, Algyő, Hungary | 5 | 3.8 | 1.09 | N47.1352 | E20.2283 |
| Atkai-Holt Tisza, Szeged, Hungary | 4 | 3.8 | 1.09 | N47.1531 | E20.2052 |
| Egyeki Holt Tisza, Egyek, Hungary | 18 | 1 | 0.87 | N47.1631 | E20.2171 |
| Egyek-Kócsi Tározó, Górés, Hungary | 15 | 1 | 0.36 | N47.5642 | E20.9271 |
| Fancsika 1, Debrecen, Hungary | 5 | 1.5 | 0.85 | N47.51153 | E21.7138 |
| Fancsika 2, Debrecen, Hungary | 4 | 1 | 0.025 | N47.5011 | E21.7352 |
| Félhalmi-holtág, Hungary | 3 | 2 | 0.73 | N46.9087 | E20.9532 |
| Galaţui, Romania | 2 | 3 | 0.75 | N44.2582 | E27.1122 |
| Holt-Szamos, Géberjén, Hungary | 9 | 1.9 | 1.43 | N47.9358 | E22.4610 |
| Holt-Szamos, Tunyogmatolcs, Hungary | 8 | 1.9 | 1.43 | N47.9764 | E22.4589 |
| Kakasszéki-tó, Székkutas, Hungary | 3 | 1.5 | 0.54 | N46.5419 | E20.5922 |
| Kati-tó, Debrecen, Hungary | 5 | 1 | 0.012 | N47.4515 | E21.6822 |
| Lake Crniševo Croatia | 5 | 31 | 0.43 | N43.04 | E17.24 |
| Lake Kozjak Croatia | 6 | 48 | 0.82 | N44.53 | E15.36 |
| Lake Oćuša Croatia | 6 | 20 | 0.75 | N43.04 | E17.25 |
| Lake Prošće  Croatia | 6 | 38 | 0.68 | N44.51 | E15.36 |
| Lake Visovačko Croatia | 6 | 30 | 5.88 | N43.50 | E15.59 |
| Lake Vransko (Biograd)  Croatia | 10 | 5 | 30.7 | N43.55 | E15.32 |
| Lake Vransko (Cres Island)  Croatia | 6 | 78 | 5.43 | N44.51 | E14.22 |
| Madarász-tó, Mórahalom, Hungary | 5 | 1.9 | 0.77 | N46.1838 | E19.9288 |
| Mézeshegyi tó, Debrecen, Hungary | 9 | 1 | 0.43 | N47.4524 | E21.7097 |
| Nagybaracskai Holt-Duna, Dunafalva, Hungary | 3 | 1 | 0.82 | N46.0599 | E18.8383 |
| Nagyréti - tározó, Hungary | 3 | 1.7 | 2.17 | N47.8928 | E21.6824 |
| Rétközi-tó, Szabolcsveresmart, Hungary | 5 | 2.3 | 3.39 | N48.2943 | E22.0353 |
| Serházzugi Holt-Tisza, Csongrád, Hungary | 7 | 2 | 1.34 | N46.7048 | E20.1512 |
| Snagov, Romania | 8 | 11 | 5.75 | N44.7112 | E26.1662 |
| Szarvas-Békésszentandrási holtág, Hungary | 6 | 2.2 | 2.07 | N46.8881 | E20.4980 |
| Szarvasi-holtág, Hungary | 2 | 2.2 | 2.07 | N46.8230 | E20.5285 |
| Szelidi-tó, Dunapataj, Hungary | 8 | 2.6 | 0.52 | N46.6304 | E19.0484 |
| Tiszadobi Holt-Tisza, Darab Tisza, Hungary | 19 | 1 | 0.07 | N48.0211 | E21.2138 |
| Tiszadobi Holt-Tisza, Falu-Tisza, Hungary | 13 | 3.1 | 0.21 | N48.0129 | E21.1714 |
| Tiszadobi Holt-Tisza, Felső Darab Tisza, Hungary | 11 | 1 | 0.03 | N48.0176 | E21.2293 |
| Tiszadobi Holt-Tisza, Malom-Tisza open, Hungary | 5 | 7 | 0.25 | N48.0113 | E21.1705 |
| Tiszadobi Holt-Tisza, Malom-Tisza flooting, Hungary | 15 | 2 | 0.05 | N48.0184 | E21.1813 |
| Tiszadobi Holt-Tisza, Szűcs- Tisza, Hungary | 22 | 1.5 | 0.06 | N48.0125 | E21.2057 |
| Vadkerti-tó, Soltvadkert, Hungary | 7 | 1.5 | 0.73 | N46.6124 | E19.3961 |
| Vidreéri halastavak, Felgyő, Hungary | 4 | 1.6 | 0.74 | N46.3832 | E20.0729 |
