# Supplementary material for: Trait convergence and trait divergence in lake phytoplankton reflect community assembly rules
Source: Sci Rep. 2020 Nov 11;10:19599. doi: 10.1038/s41598-020-76645-7 (PMC7658209; doi:10.1038/s41598-020-76645-7)
Supplement: Supplementary file 5 — Supplementary Figure S1. [file 41598_2020_76645_MOESM5_ESM.docx]

Electronic Supplementary Material: Figure S1. Effect sizes (ES) of functional traits along the first two canonical axes of the RDA. Dotted lines indicate the position of ES = 0. Positive values indicate divergence, negative ones convergence of traits.

**Trait convergence and trait divergence in lake phytoplankton reflect community assembly rules**

^1,2^Gábor Borics, ^2^Viktória B-Béres, ^3^István Bácsi, ^1^Balázs A. Lukács, ^1^E T-Krasznai, ^2,4^Zoltán Botta-Dukát, ^1,2^Gábor Várbíró^*^

^1^MTA Centre for Ecological Research, Danube Research Institute, Department of Tisza Research, 18/c. Bem square, 4026 Debrecen, Hungary

^2^MTA Centre for Ecological Research, GINOP Sustainable Ecosystems Group, 3. Klebelsberg Kuno str., H-8237 Tihany, Hungary

^3^University of Debrecen, Department of Hydrobiology, P.O. Box 57, H-4010 Debrecen, Hungary

^4^MTA Centre for Ecological Research, Institute of Ecology and Botany, 2-4. Alkotmány str., H-2163 Vácrátót, Hungary

| Traits | Distribution of the ES values against the RDA Axis one | Distribution of the ES values against the RDA Axis two |
| --- | --- | --- |
| Flagellated | 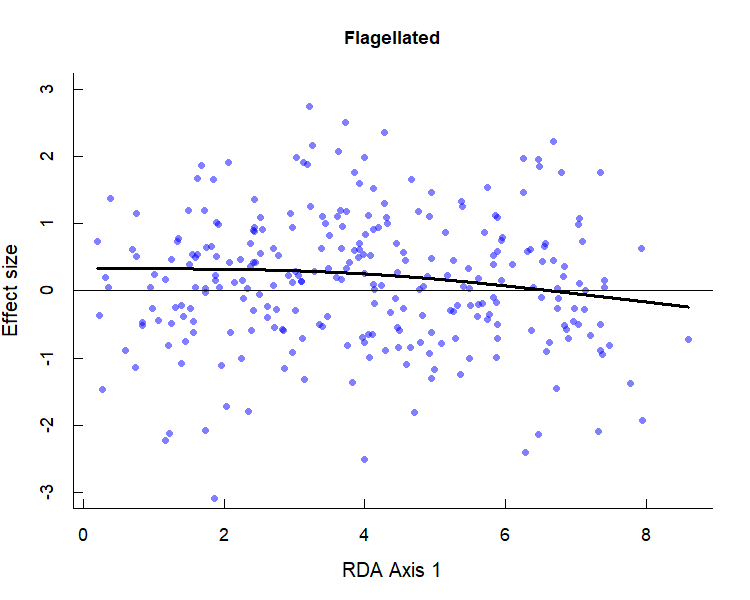 | 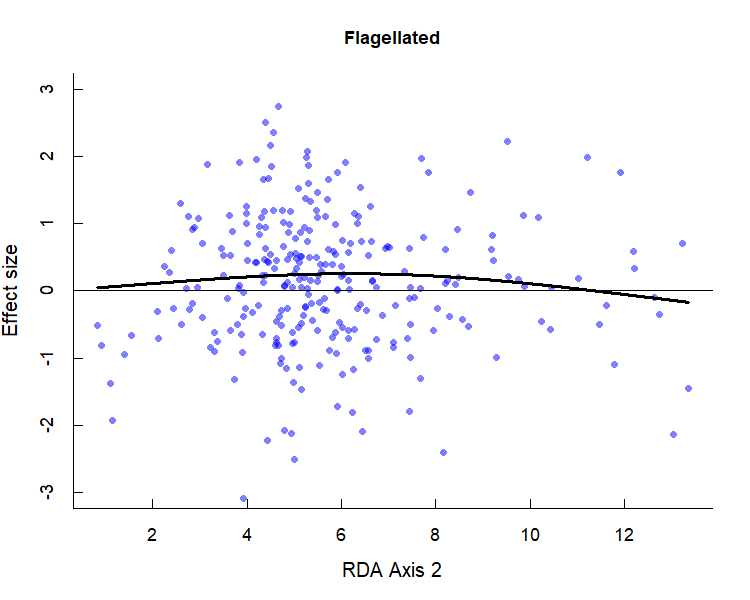 |
| Size (larger >40 µm) | 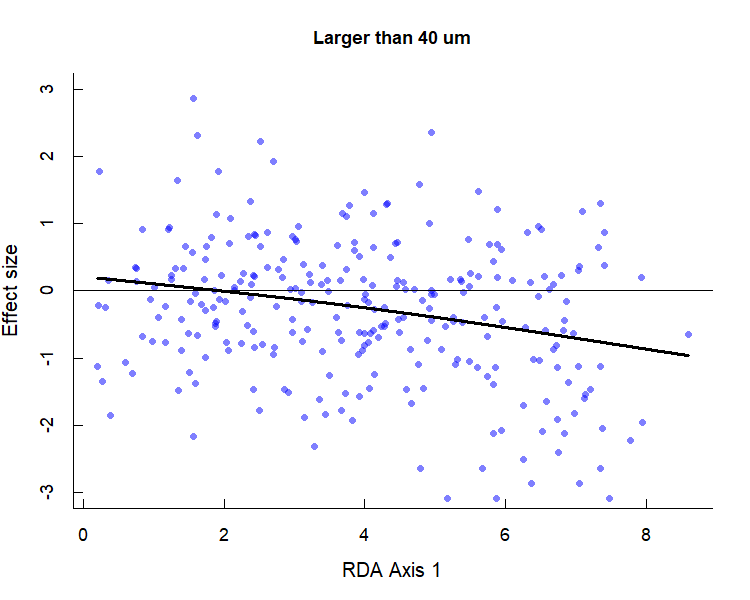 | 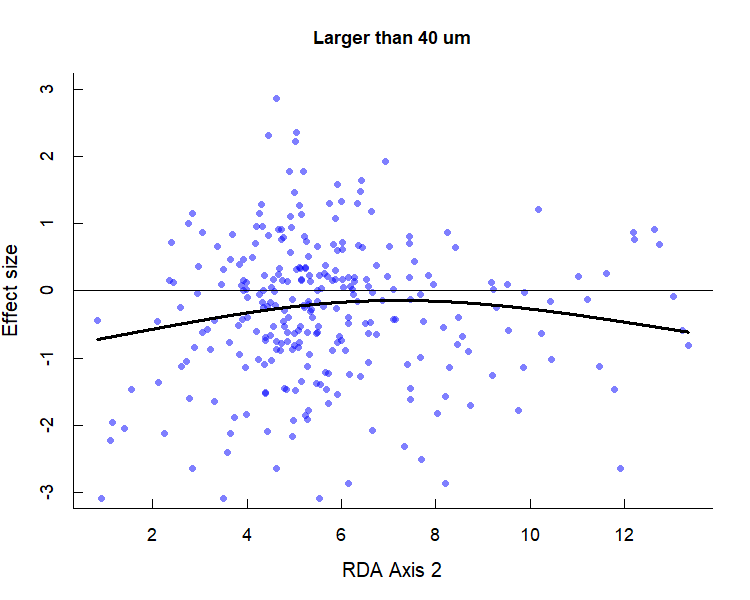 |
| Colonial | 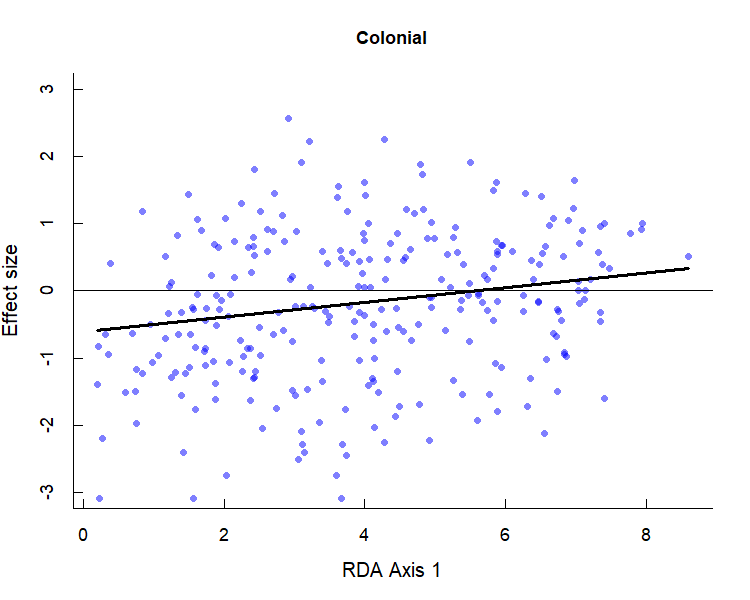 | 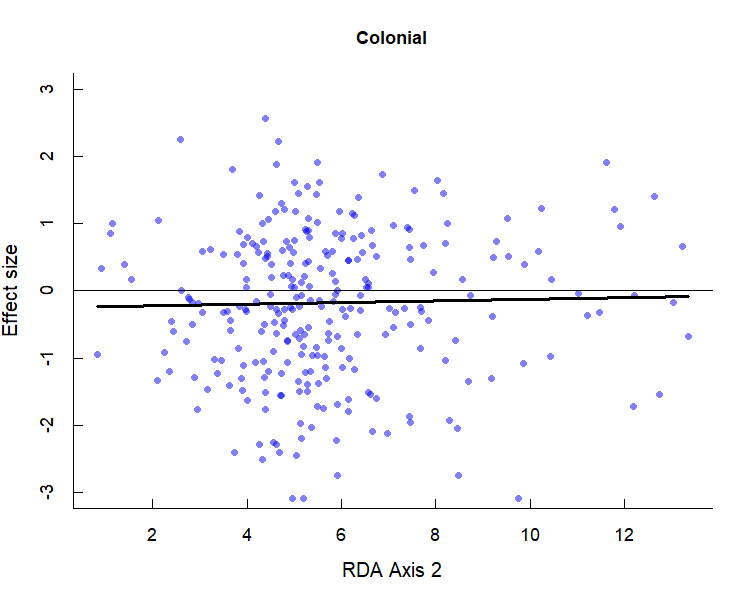 |
| Single celled | 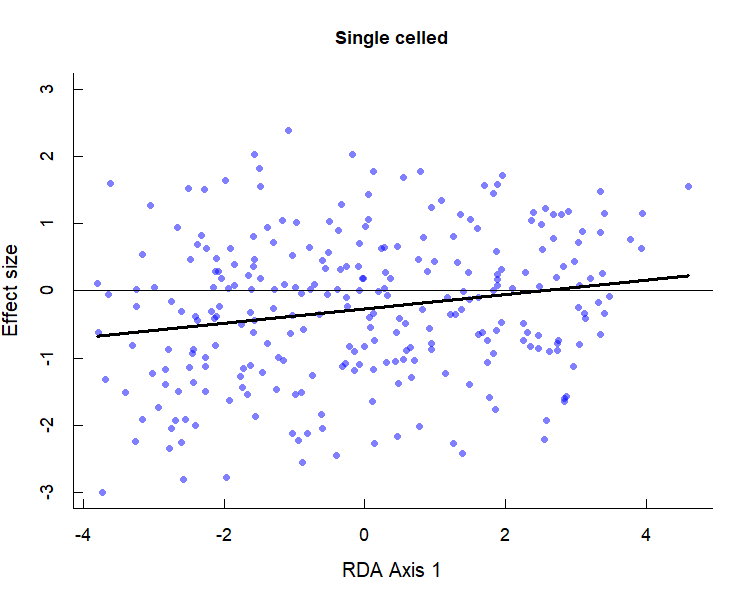 | 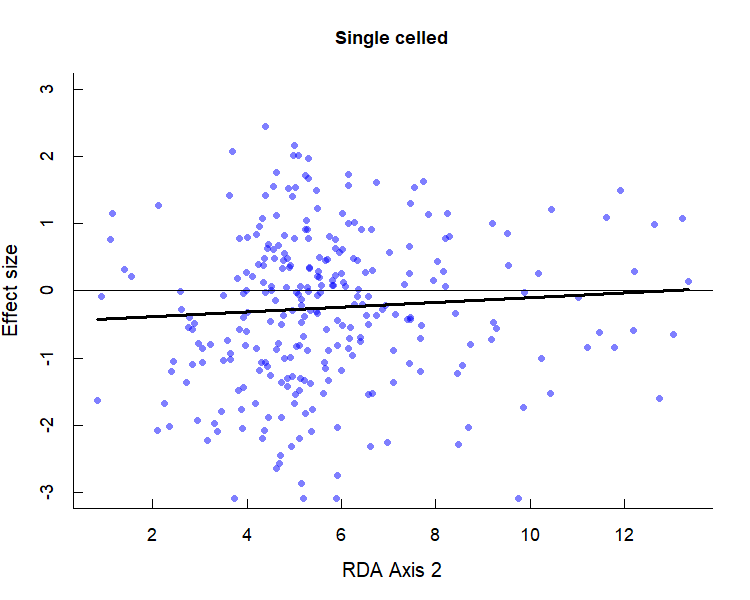 |
| Filamentous | 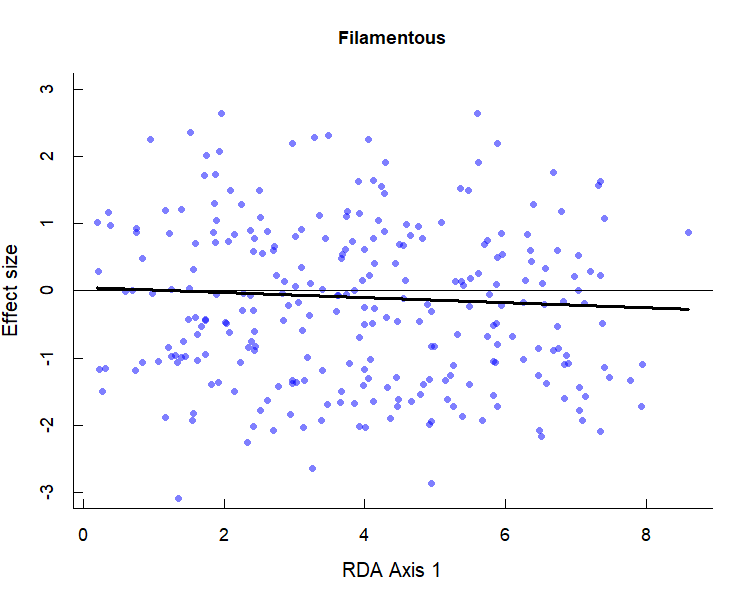 | 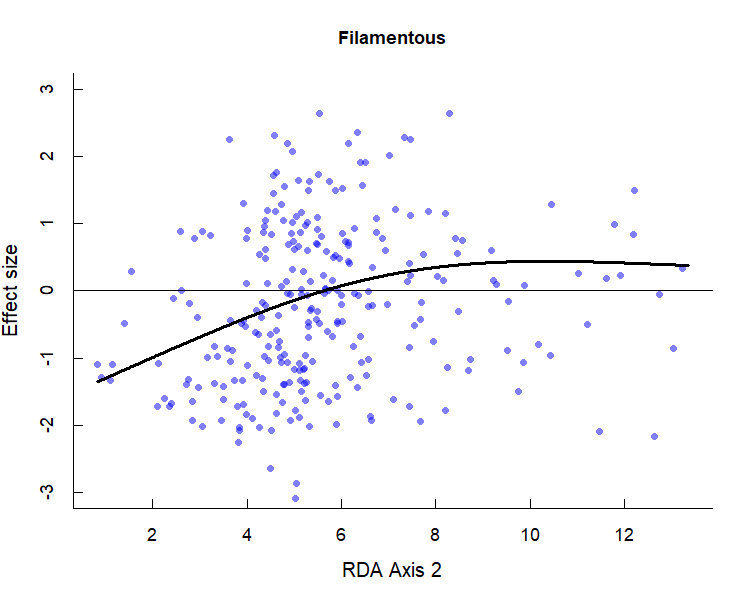 |
| Mixotrophic | 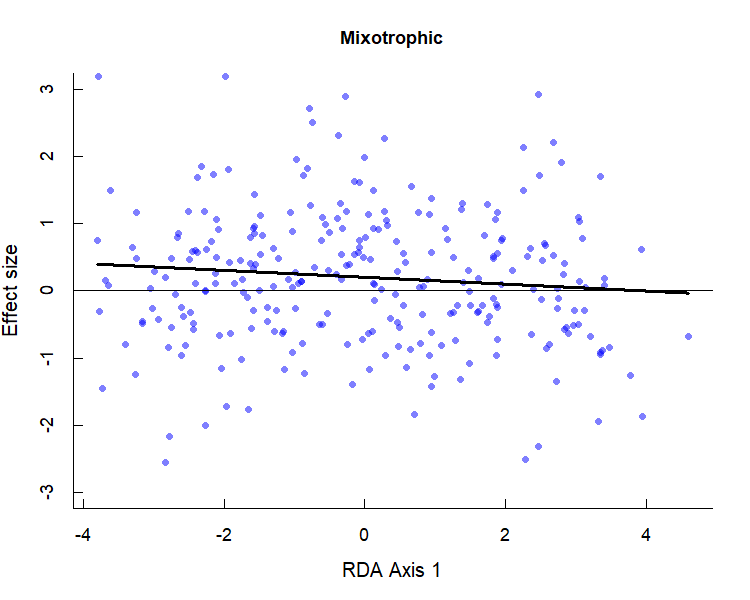 | 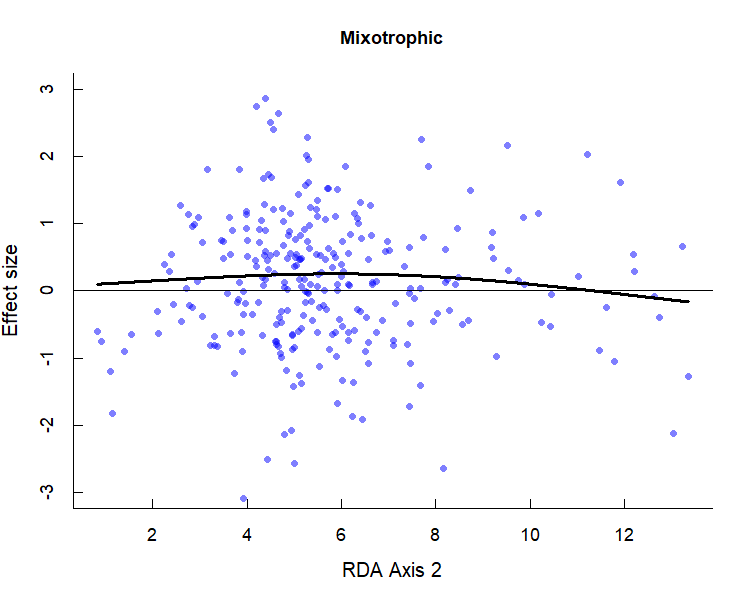 |
| Silicious | 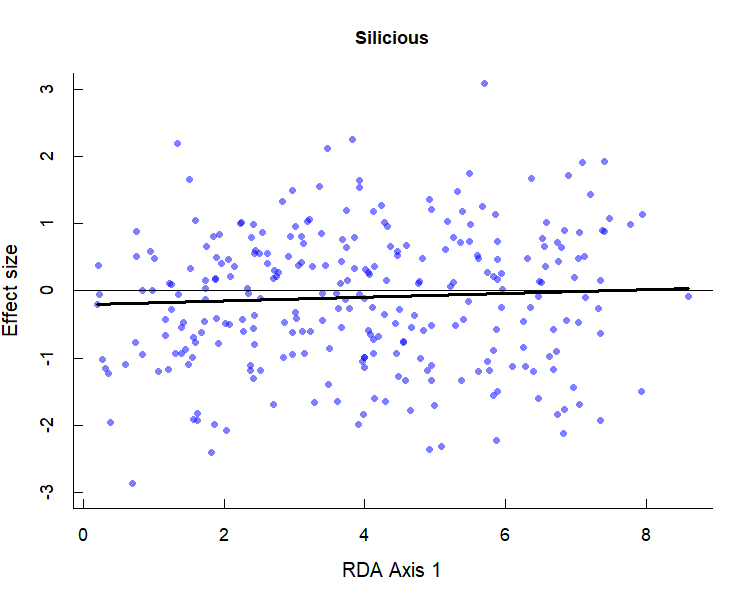 | 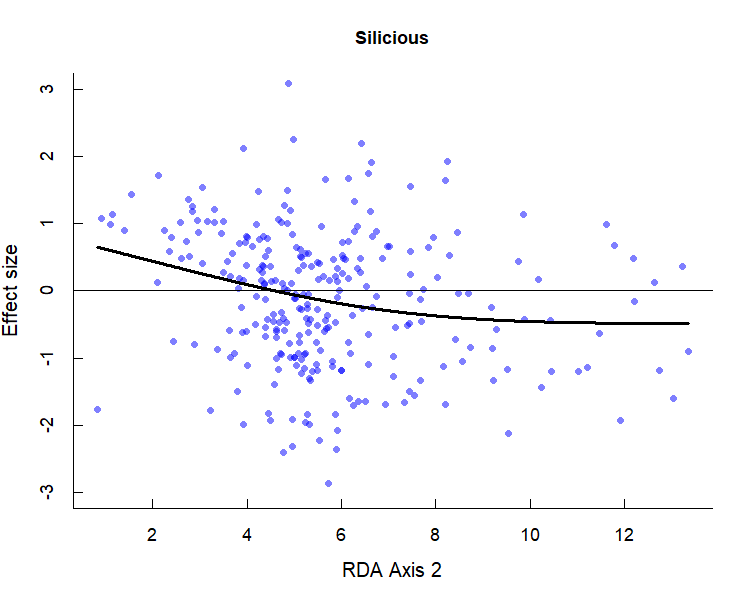 |
| Nitrogen-fixing | 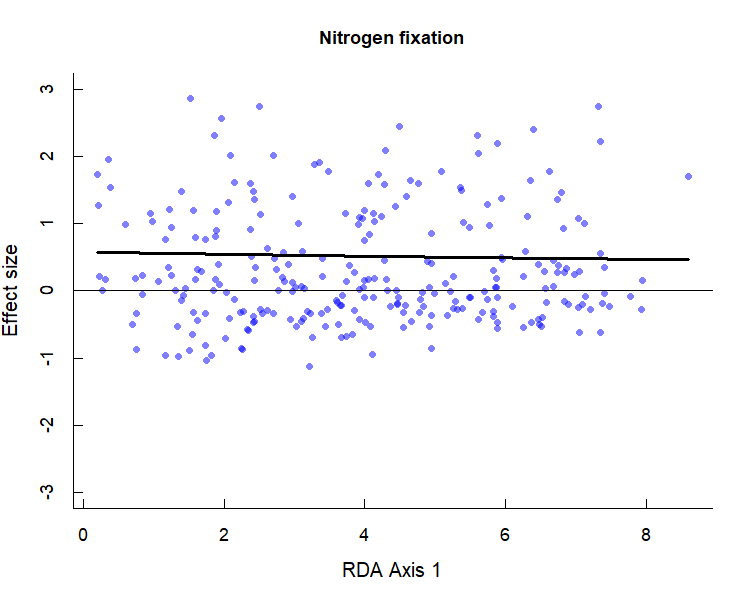 | 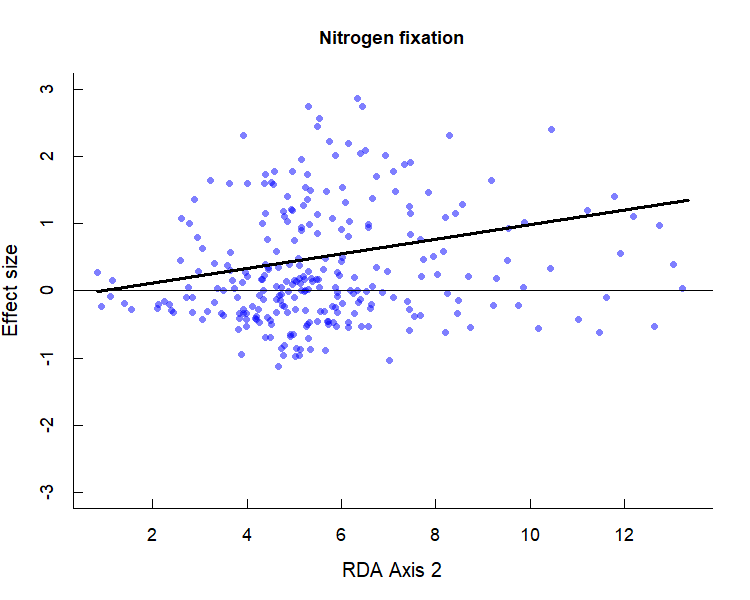 |
| Vacuolated | 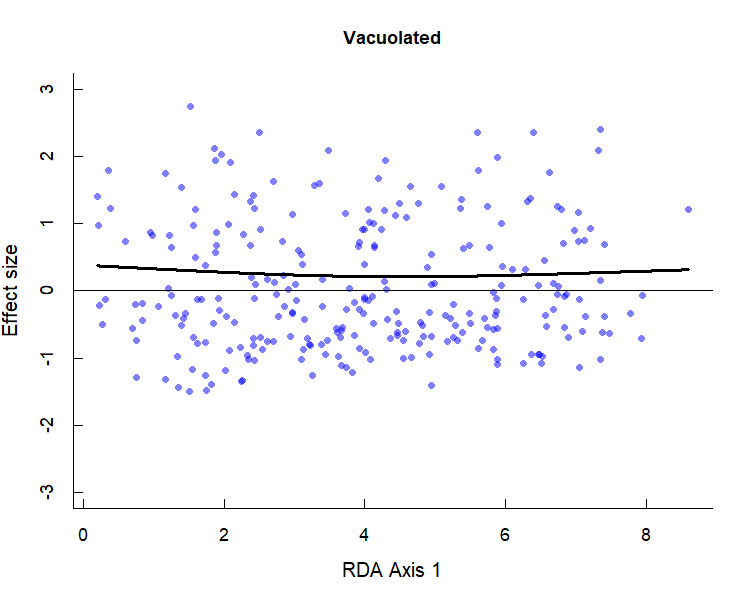 | 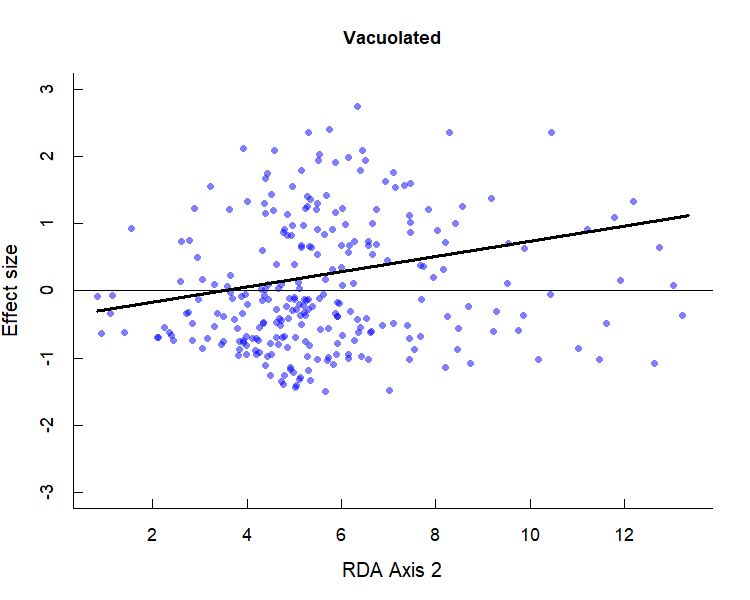 |
| Large Flagellated | 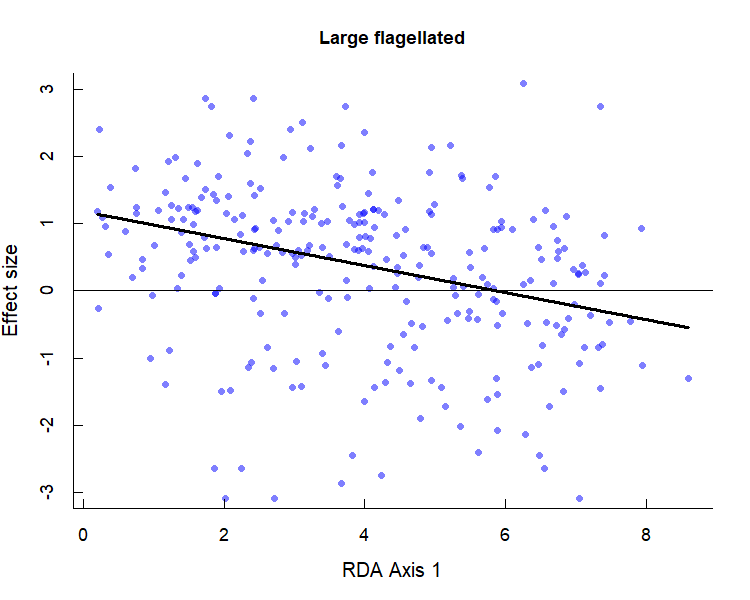 | 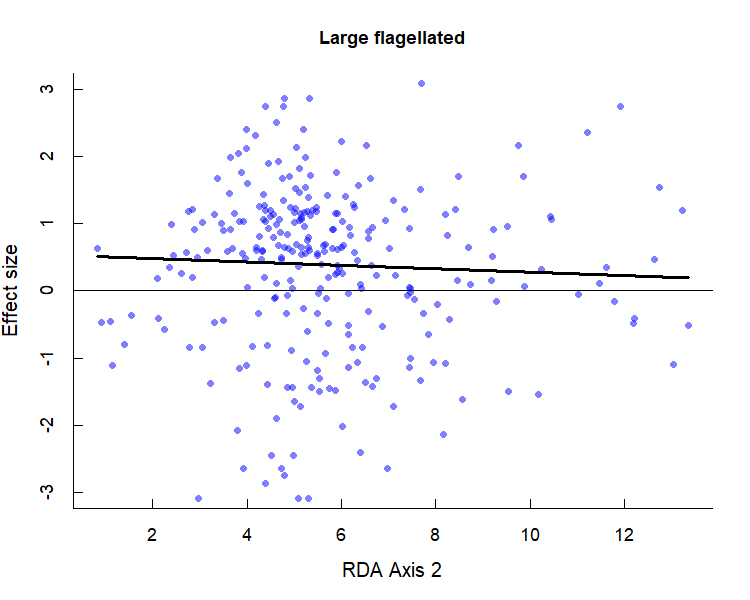 |
|  |  |  |
