# Supplementary material for: Trait convergence and trait divergence in lake phytoplankton reflect community assembly rules
Source: Sci Rep. 2020 Nov 11;10:19599. doi: 10.1038/s41598-020-76645-7 (PMC7658209; doi:10.1038/s41598-020-76645-7)
Supplement: Supplementary file 6 — Supplementary Figures S2–S10. [file 41598_2020_76645_MOESM6_ESM.docx]

Electronic Supplementary Material:

**Trait convergence and trait divergence in lake phytoplankton reflect community assembly rules**

^1,2^Gábor Borics, ^2^Viktória B-Béres, ^3^István Bácsi, ^1^Balázs A. Lukács, ^1^E T-Krasznai, ^2,4^Zoltán Botta-Dukát, ^1,2^Gábor Várbíró^*^

^1^MTA Centre for Ecological Research, Danube Research Institute, Department of Tisza Research, 18/c. Bem square, 4026 Debrecen, Hungary

^2^MTA Centre for Ecological Research, GINOP Sustainable Ecosystems Group, 3. Klebelsberg Kuno str., H-8237 Tihany, Hungary

^3^University of Debrecen, Department of Hydrobiology, P.O. Box 57, H-4010 Debrecen, Hungary

^4^MTA Centre for Ecological Research, Institute of Ecology and Botany, 2-4. Alkotmány str., H-2163 Vácrátót, Hungary

Figure S2. Distribution of the ES values and distribution of the CWM values against numbers of Weeks as Time. Each dot represents a sample on the gradient. Curves indicate the GAM models’ trendlines.

| Traits | Distribution of the ES values against the variables | Distribution of the CWM values against the variables |
| --- | --- | --- |
| Flagellated | 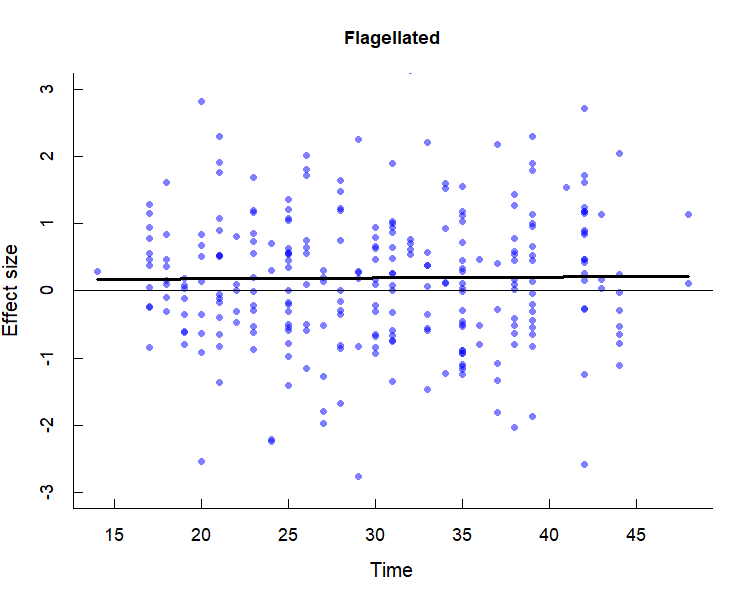 | 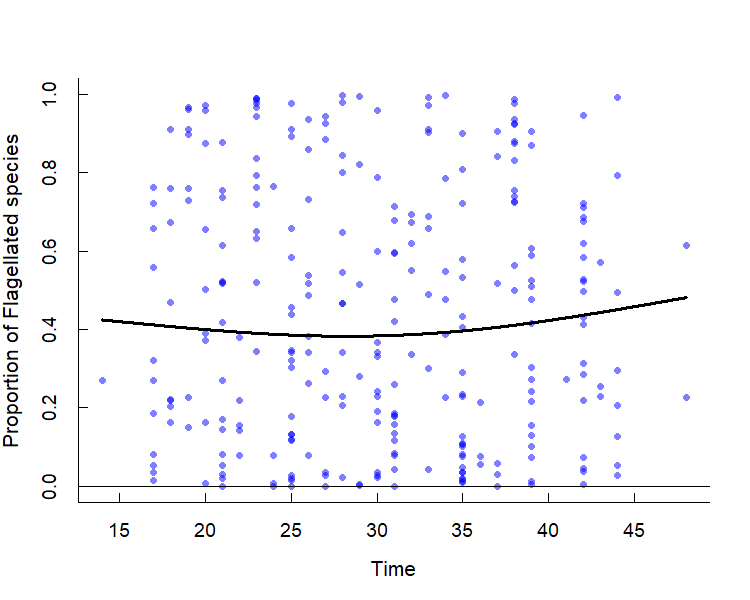 |
| Size (larger >40 µm) | 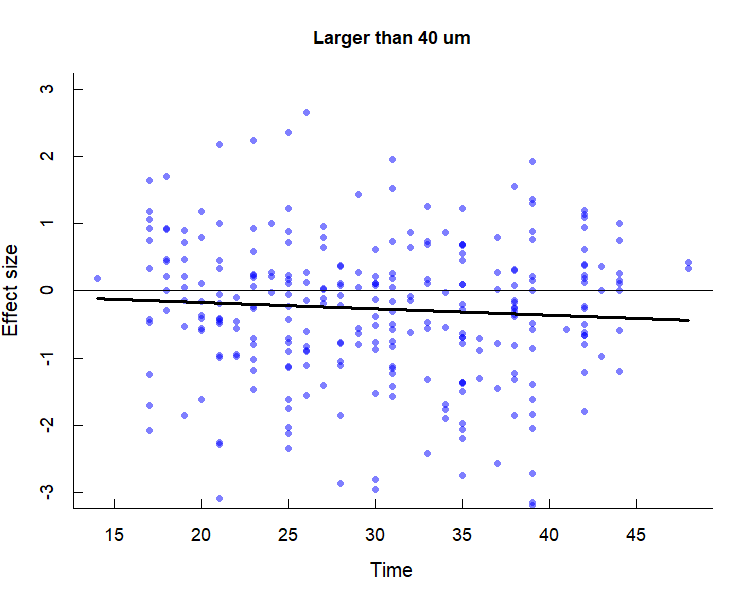 | 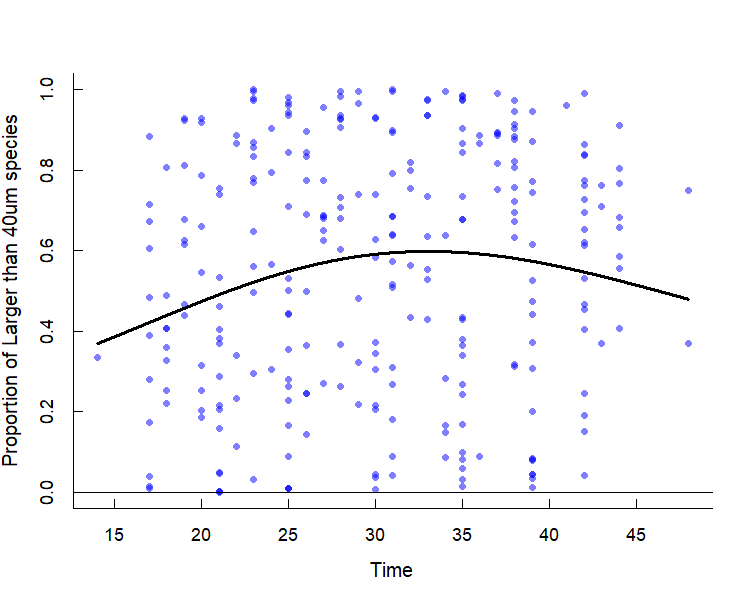 |
| Colonial | 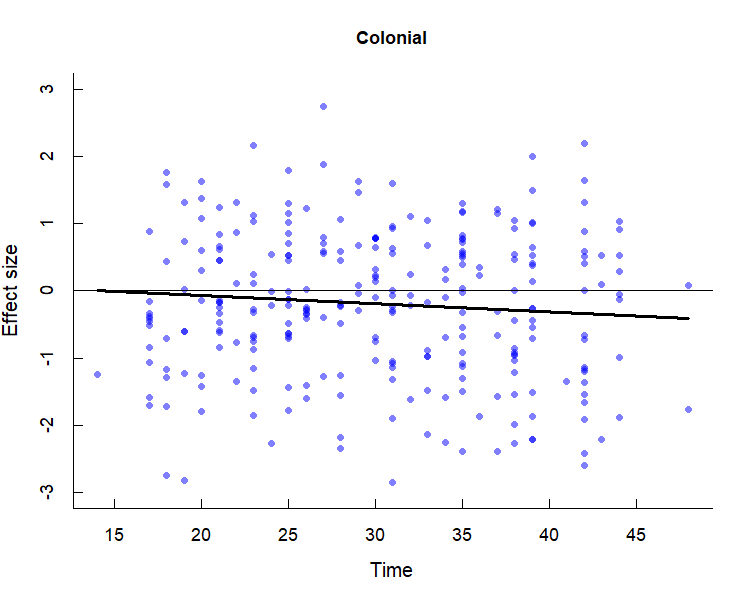 | 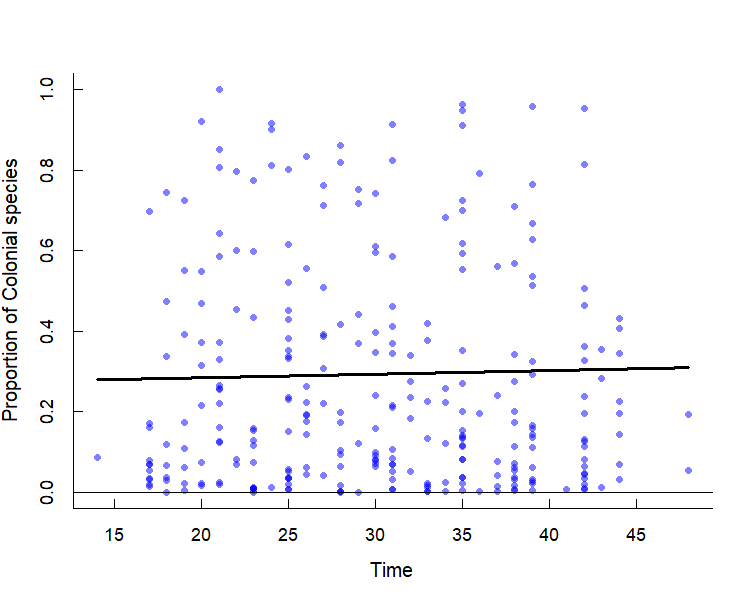 |
| Single celled | 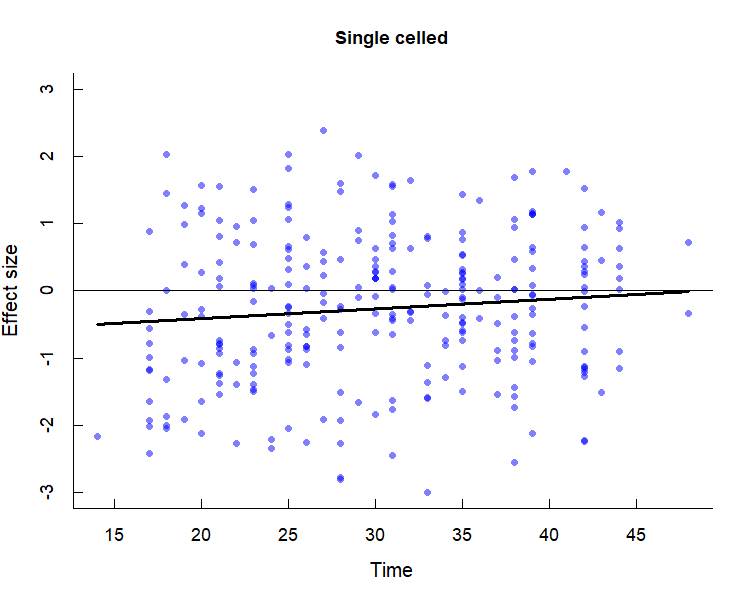 | 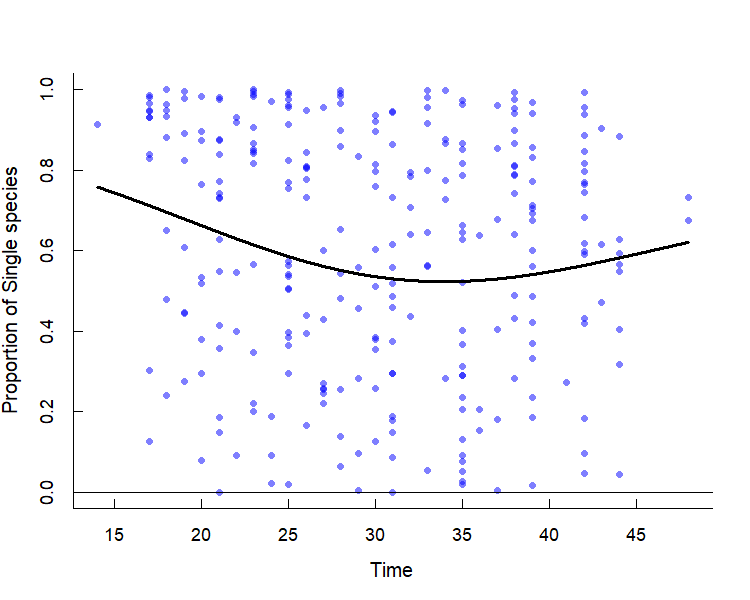 |
| Filamentous | 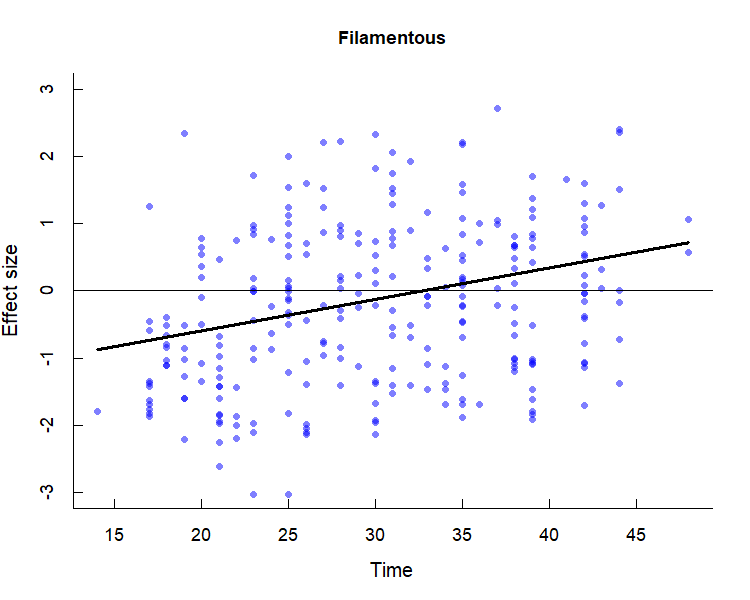 | 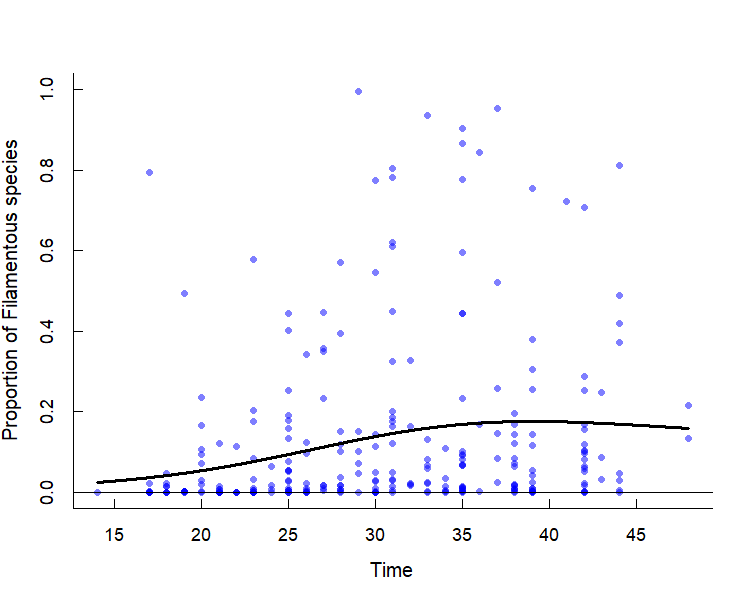 |
| Mixotrophic | 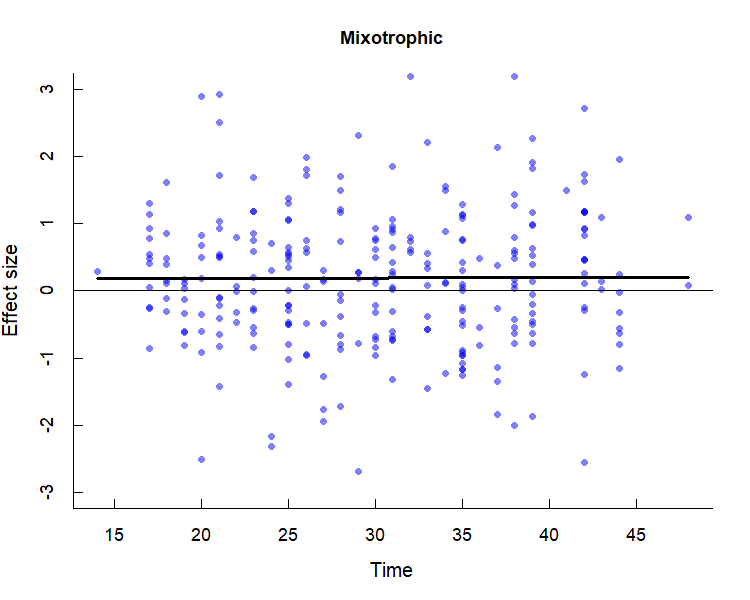 | 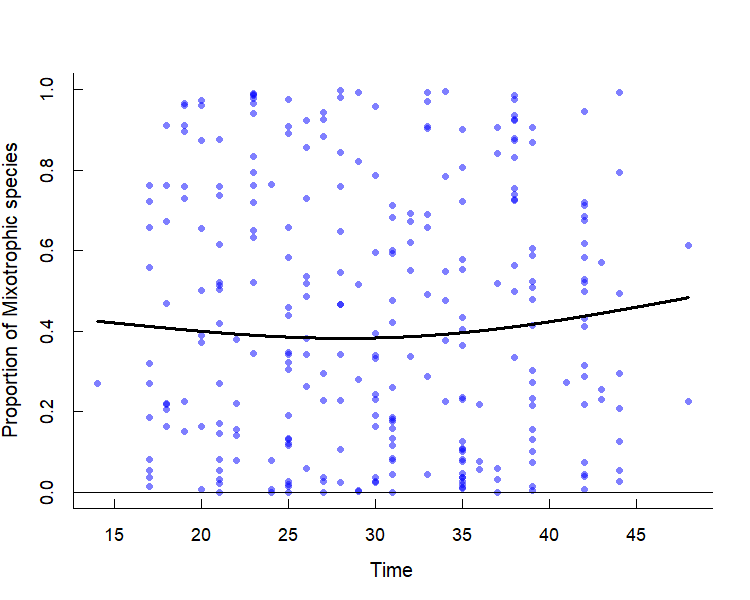 |
| Silicious | 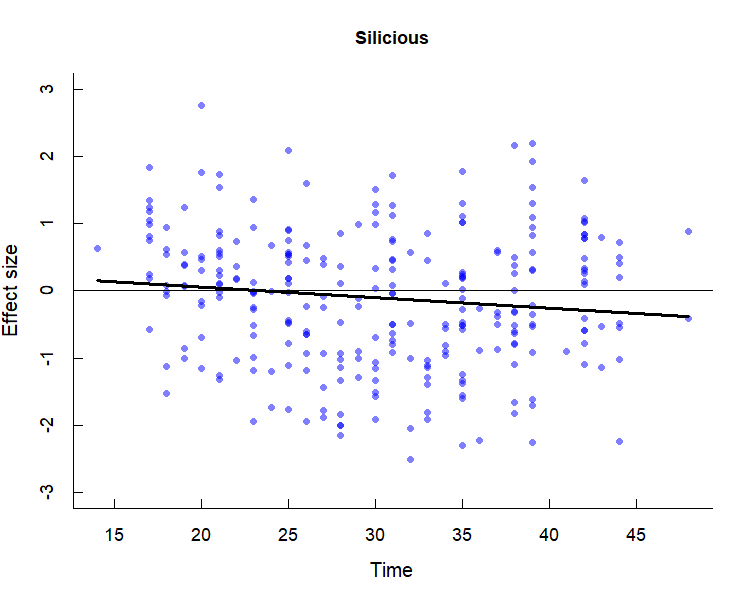 | 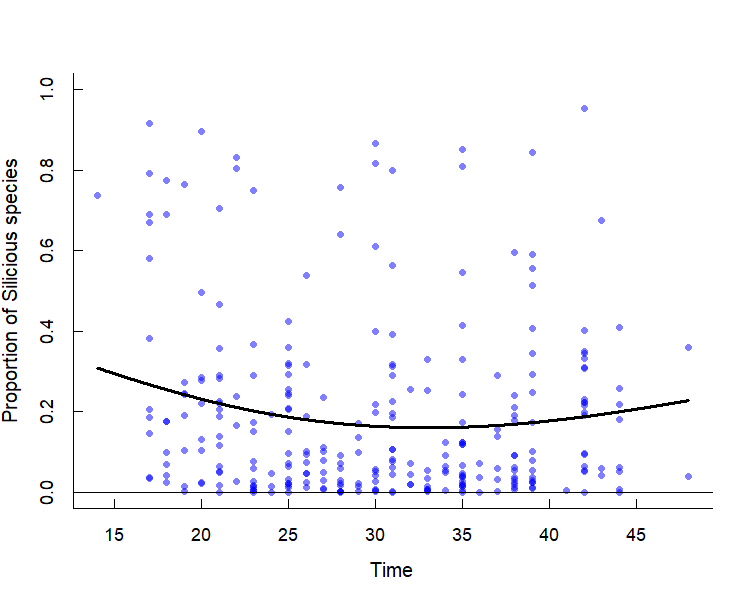 |
| Nitrogen-fixing | 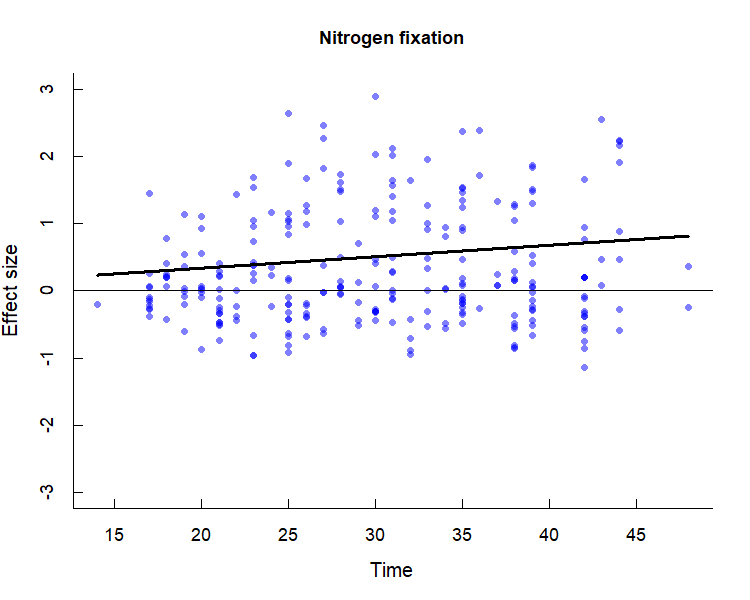 | 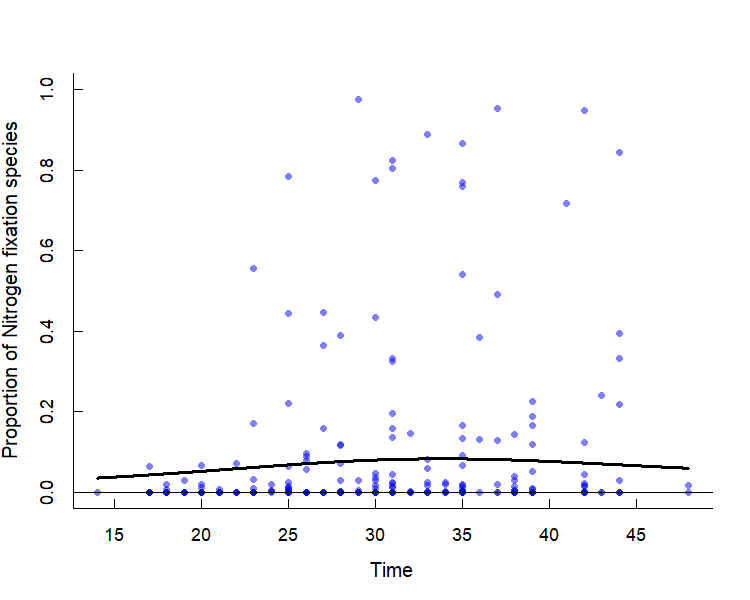 |
| Vacuolated | 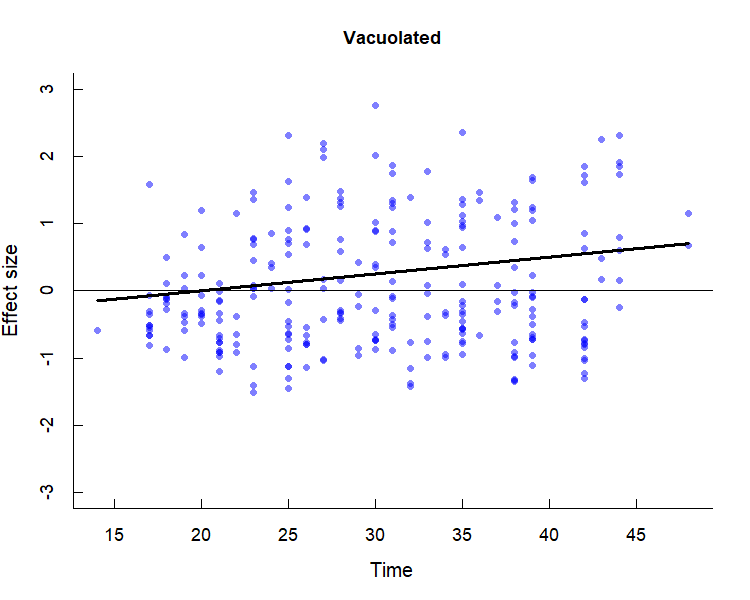 | 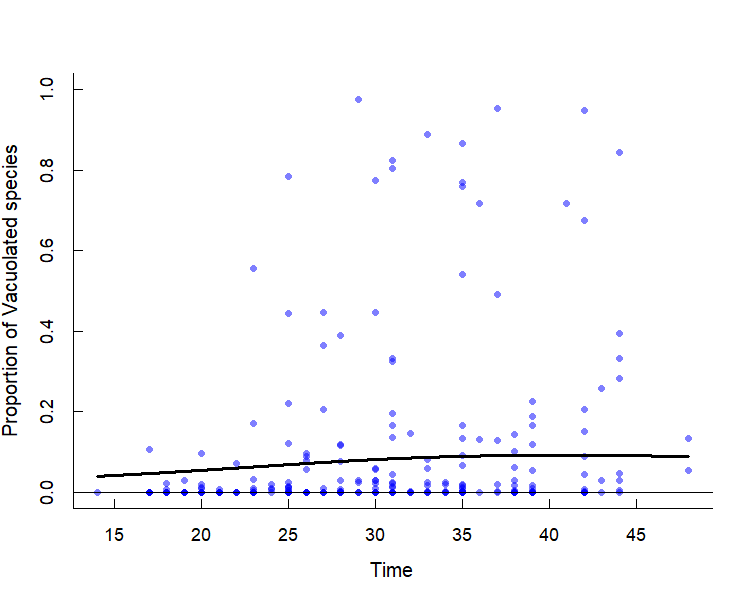 |
| Large Flagellated | 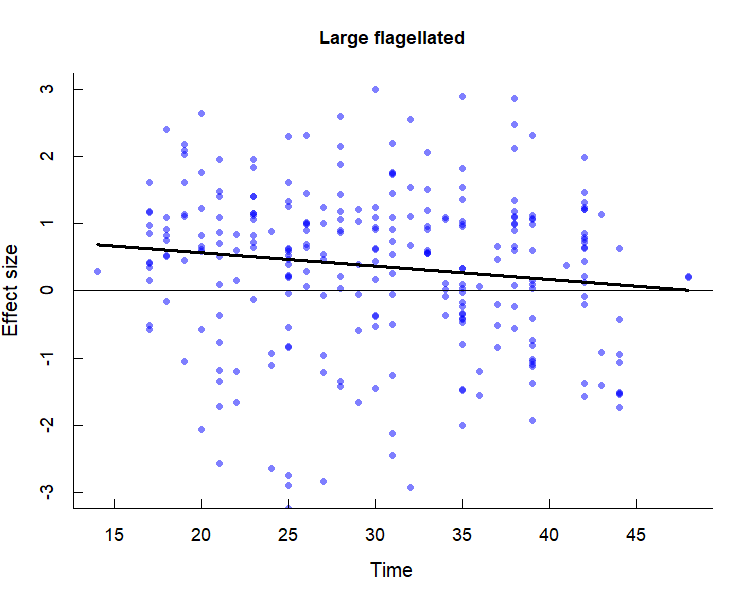 | 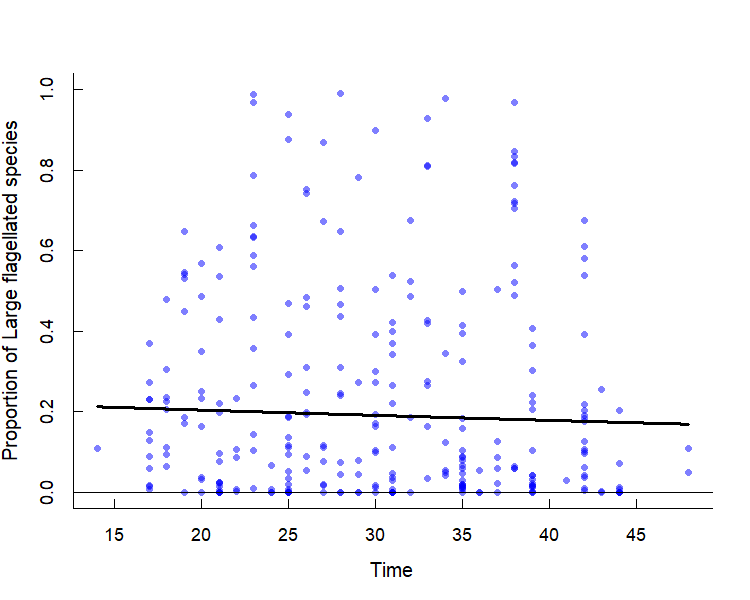 |
|  |  |  |

Figure S3. Distribution of the ES values and distribution of the CWM values against Total Phosphorus (log (µgL^-1^). Each dot represents a sample on the gradient. Curves indicate the GAM models’ trendlines.

| Traits | Distribution of the ES values against the variables | Distribution of the CWM values against the variables |
| --- | --- | --- |
|  |  |  |
| Flagellated | 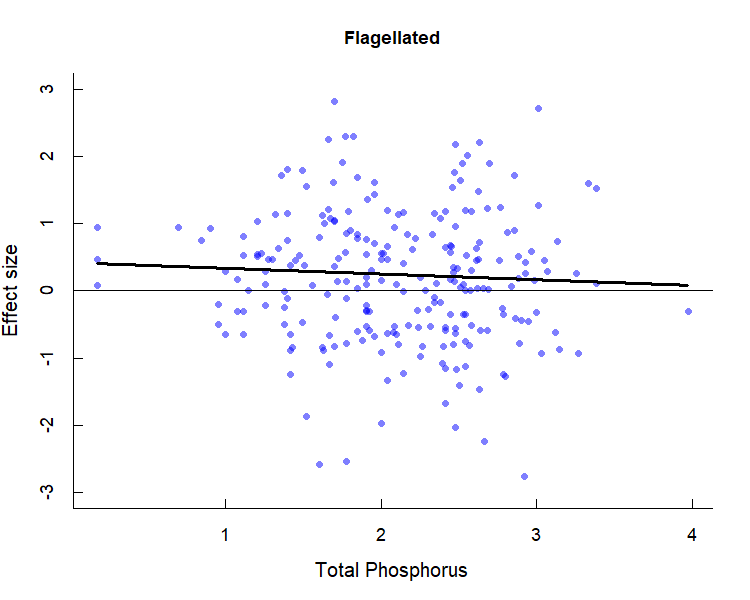 | 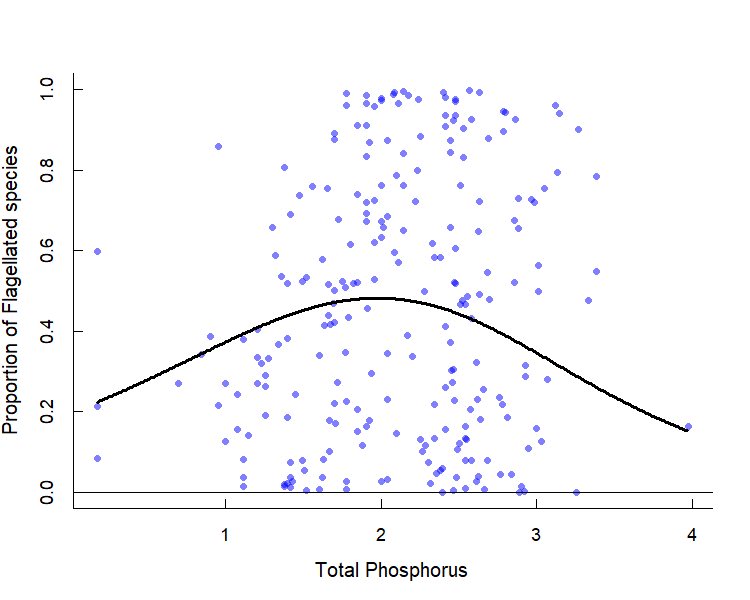 |
| Size (larger >40 µm) | 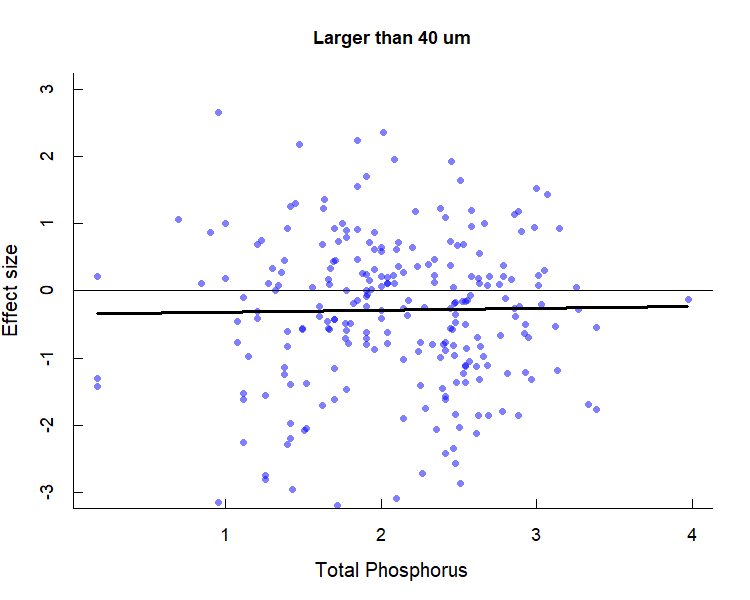 | 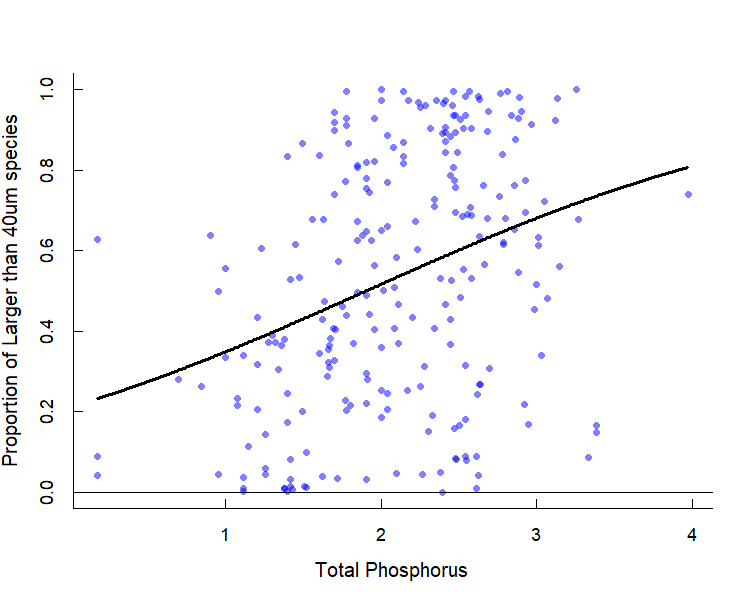 |
| Colonial | 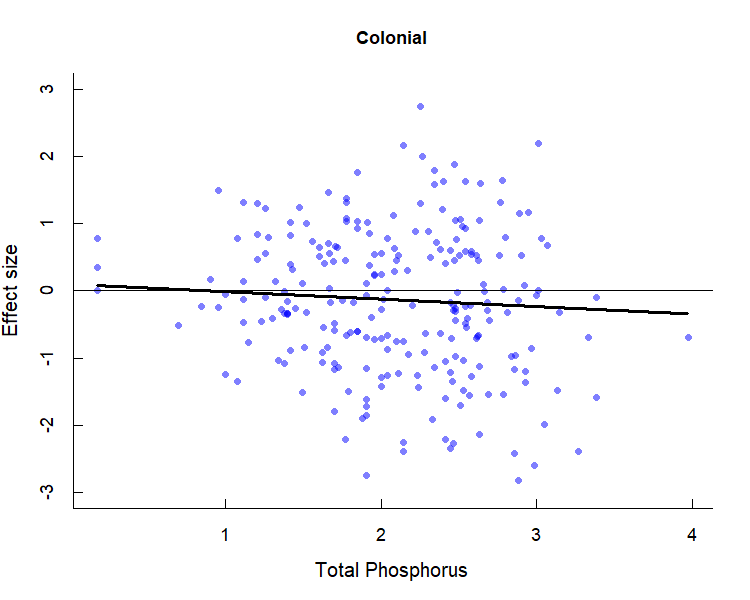 | 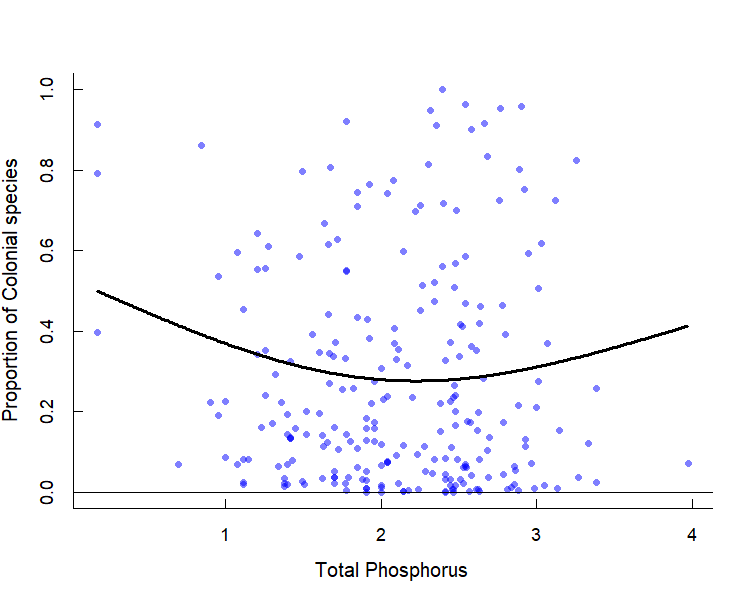 |
| Single celled | 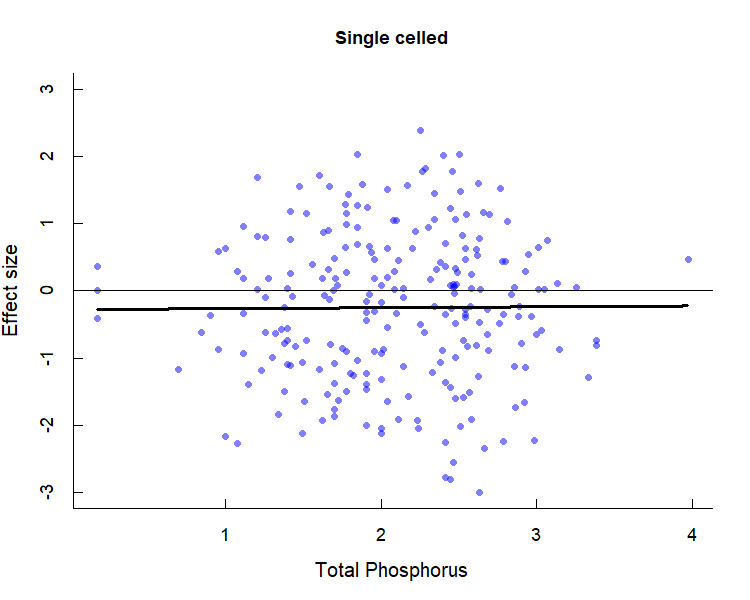 | 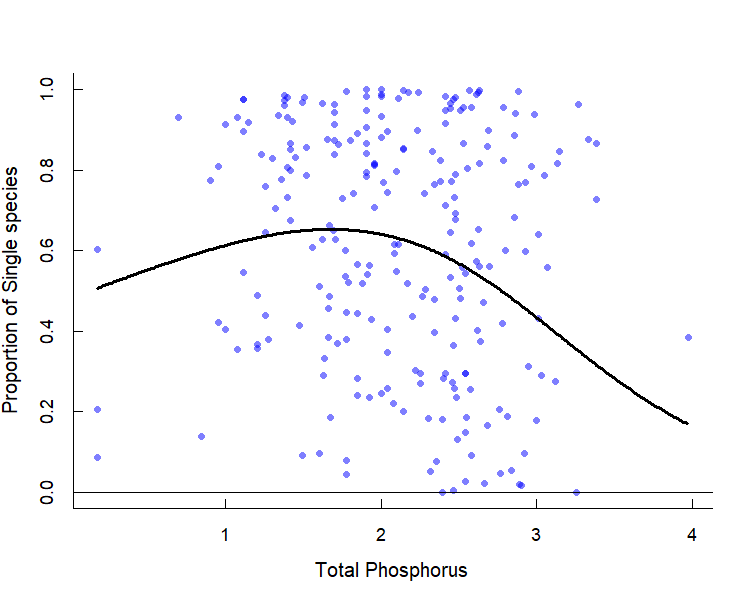 |
| Filamentous | 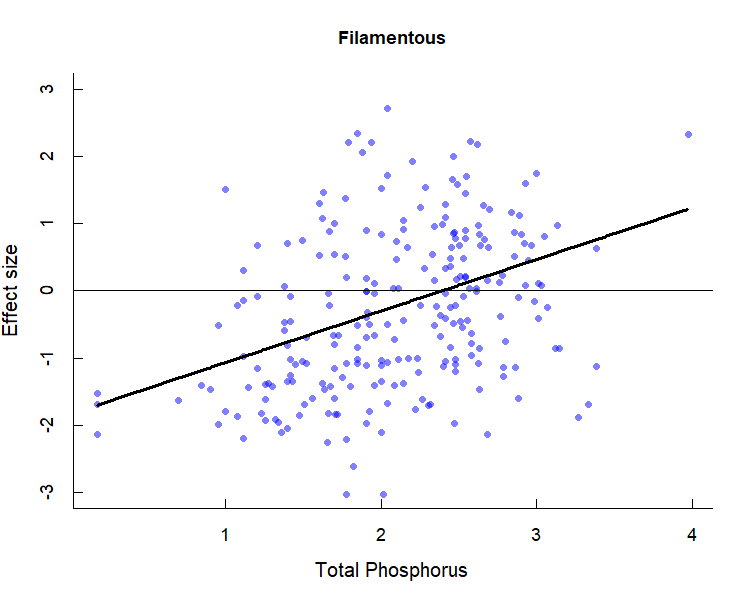 | 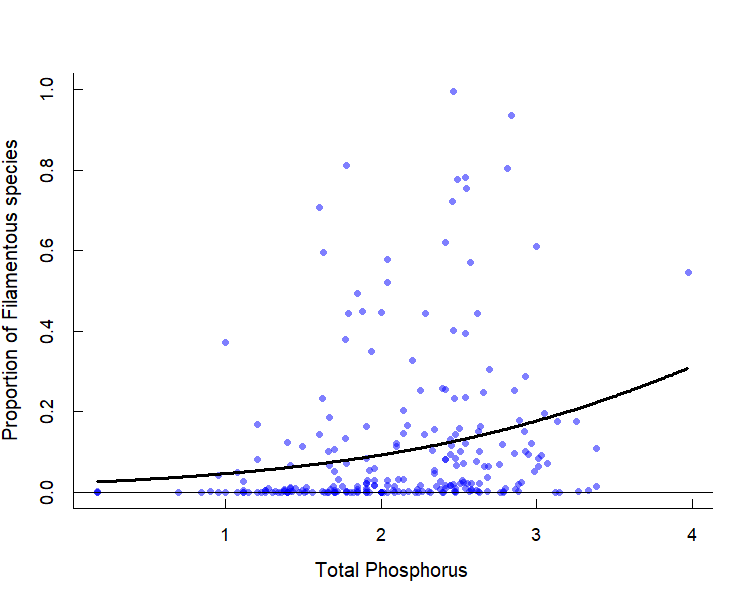 |
| Mixotrophic | 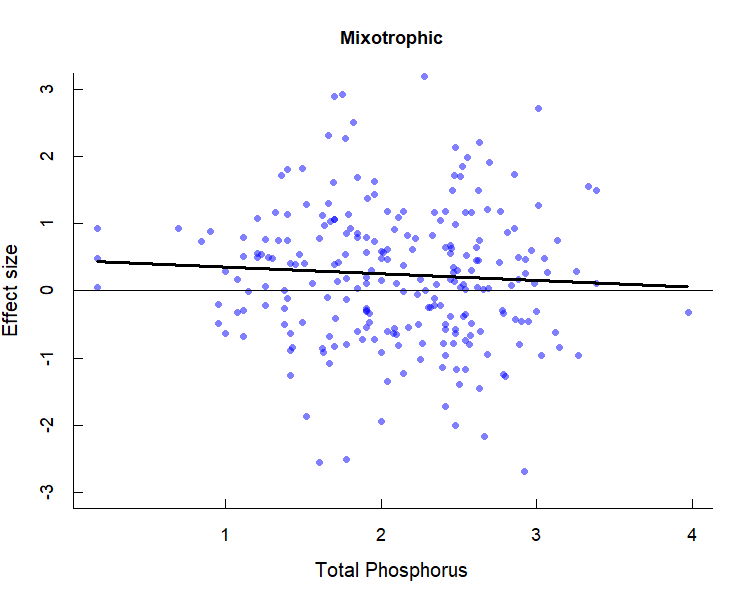 | 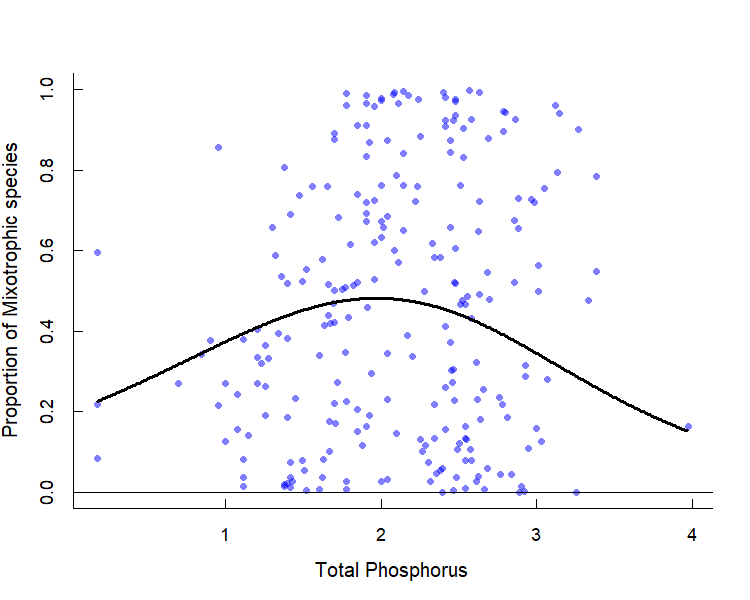 |
| Silicious | 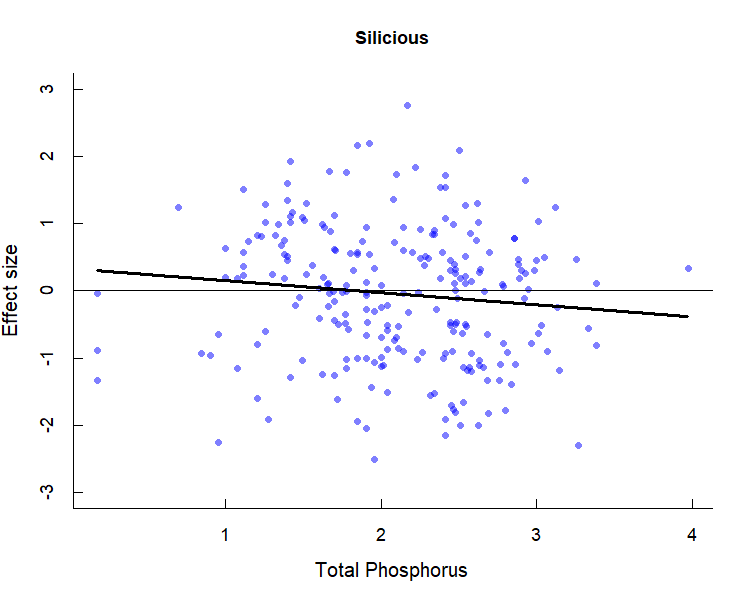 | 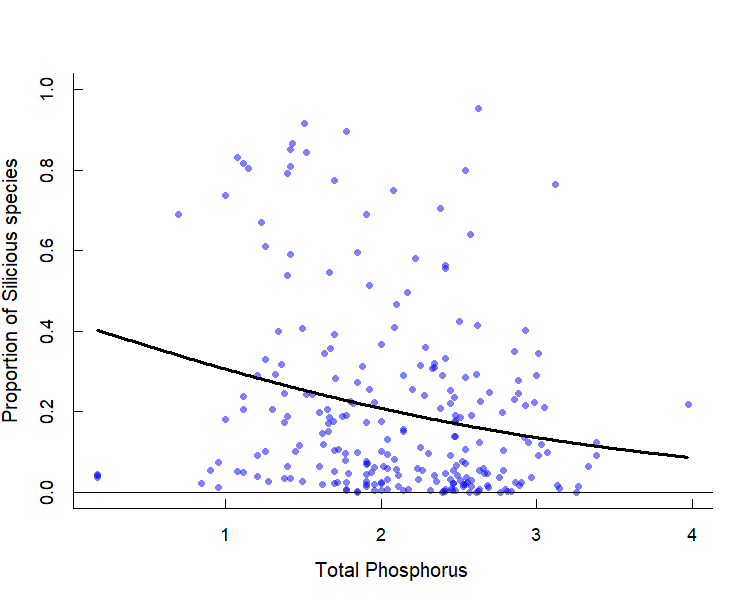 |
| Nitrogen-fixing | 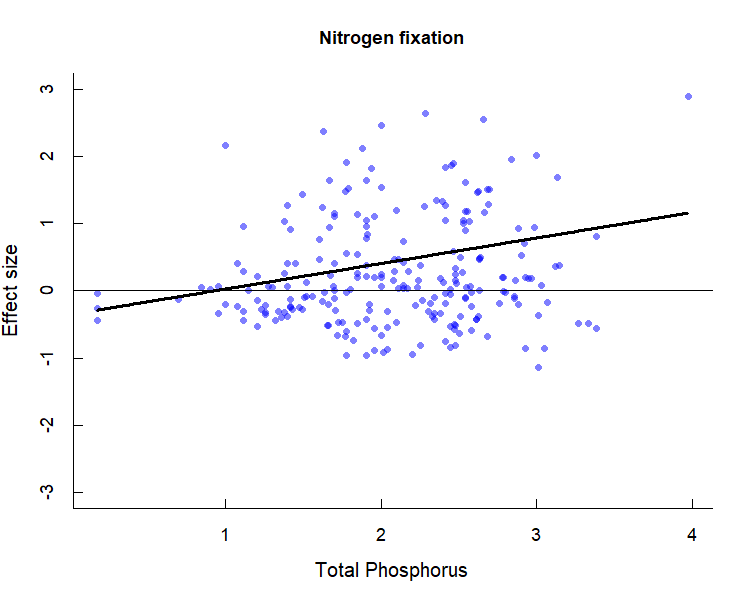 | 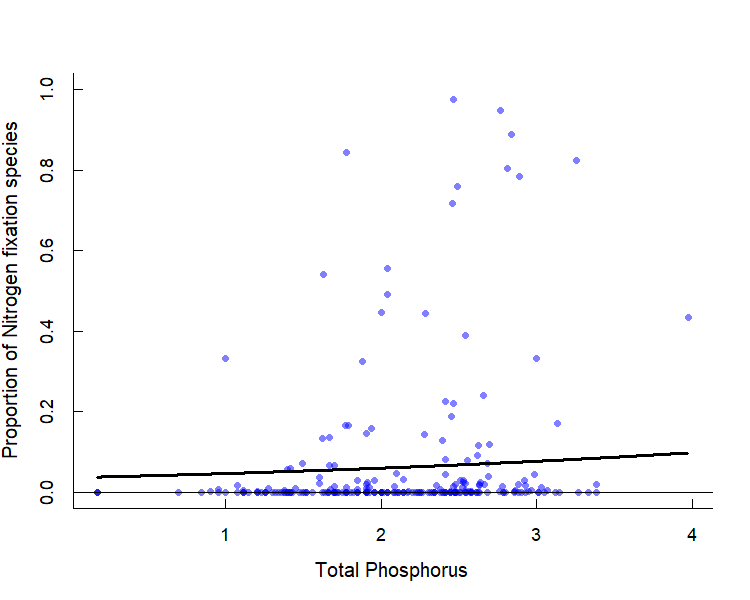 |
| Vacuolated | 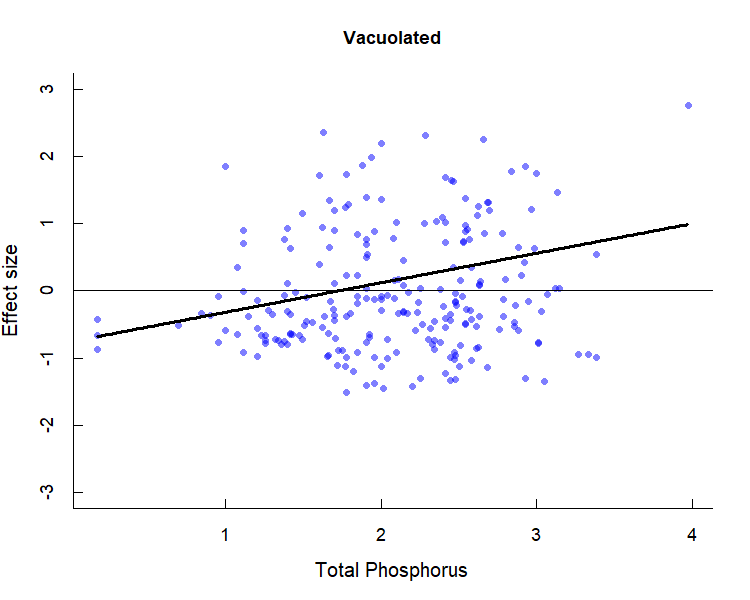 | 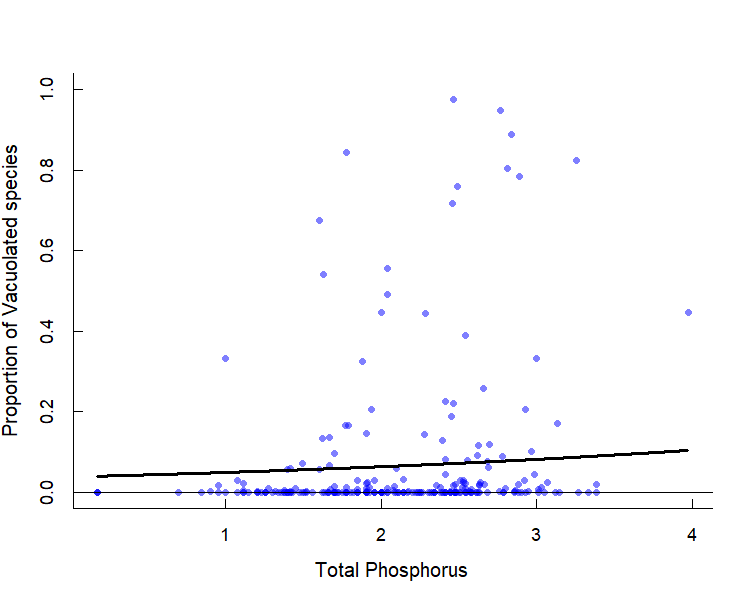 |
| Large Flagellated | 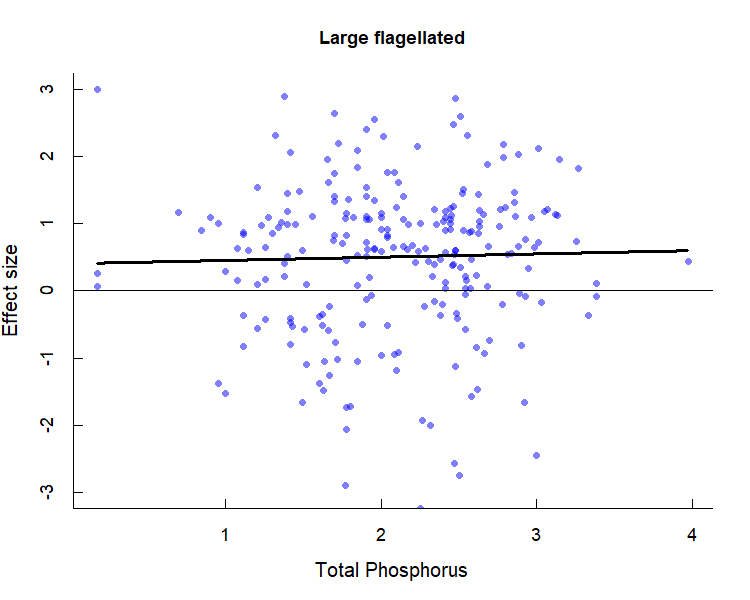 | 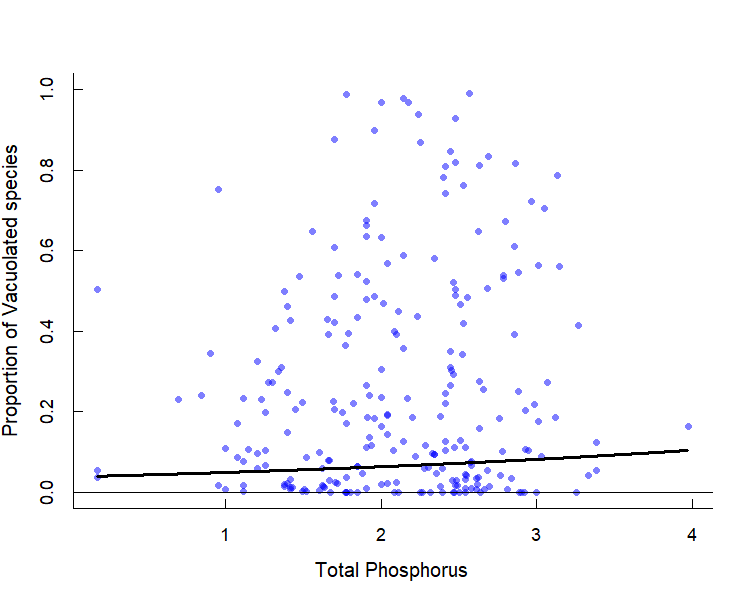 |

Figure S4. Distribution of the ES values and distribution of the CWM values against Total Nitrogen(log (µgL^-1^). Each dot represents a sample on the gradient. Curves indicate the GAM models’ trendlines.

| Traits | Distribution of the ES values against the variables | Distribution of the CWM values against the variables |
| --- | --- | --- |
|  |  |  |
| Flagellated | 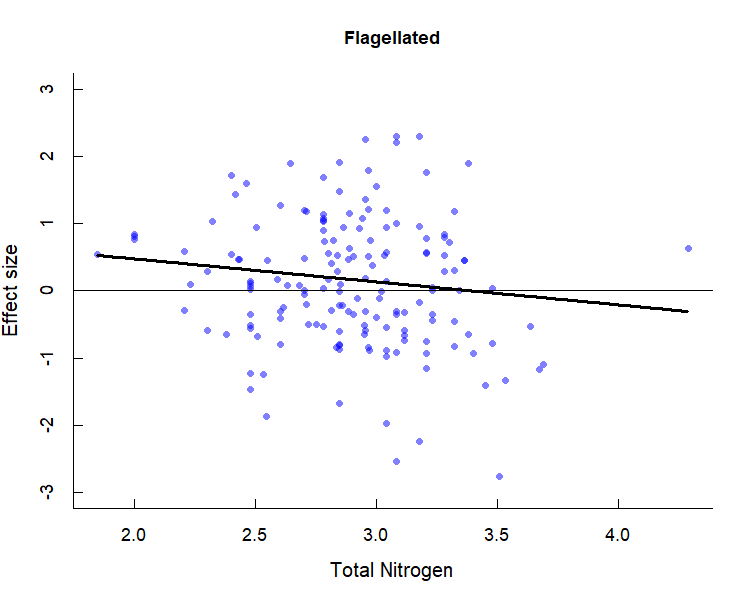 | 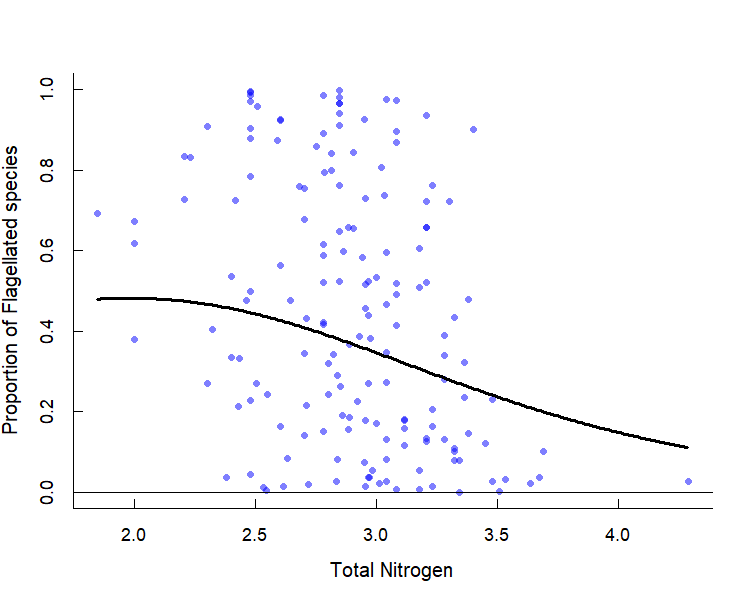 |
| Size (larger >40 µm) | 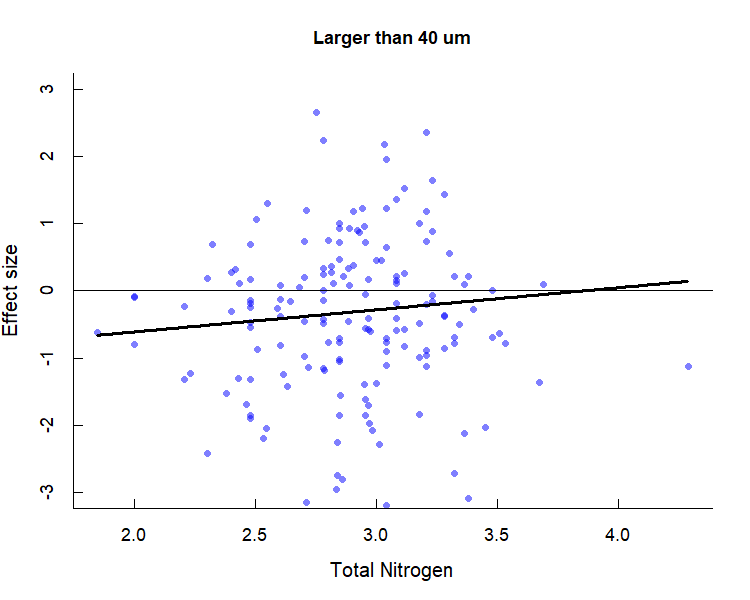 | 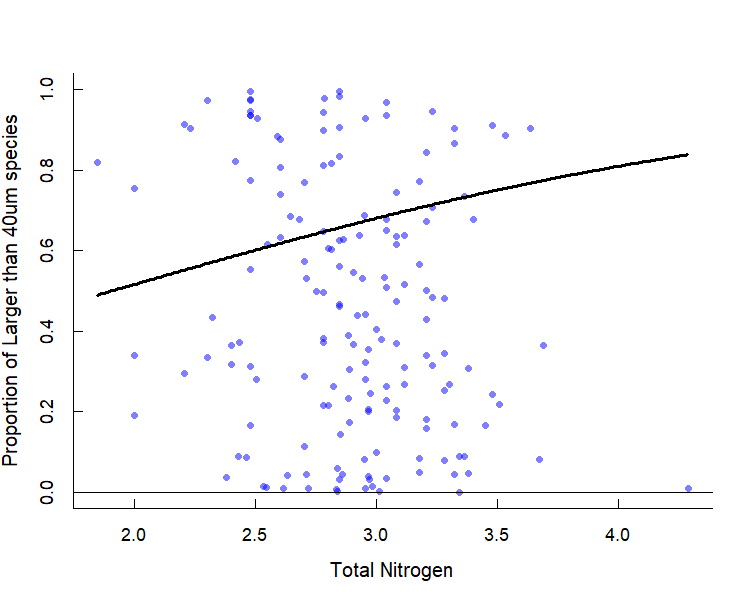 |
| Colonial | 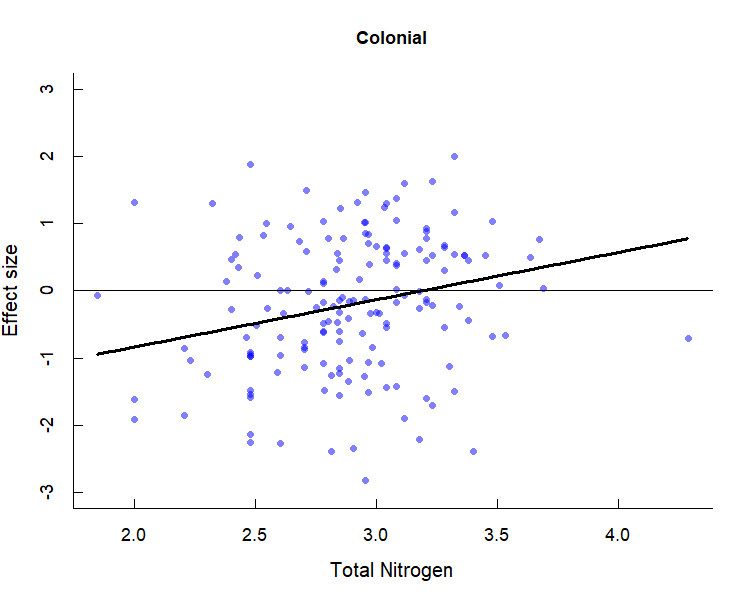 | 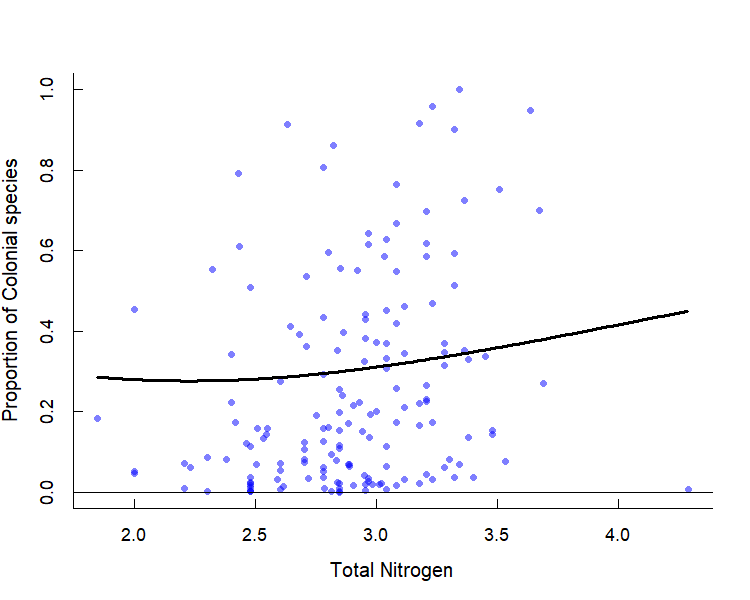 |
| Single celled | 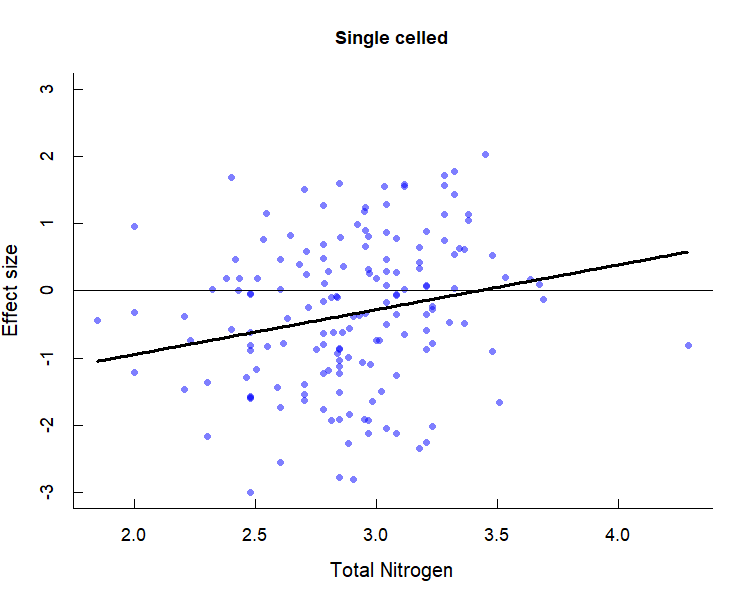 | 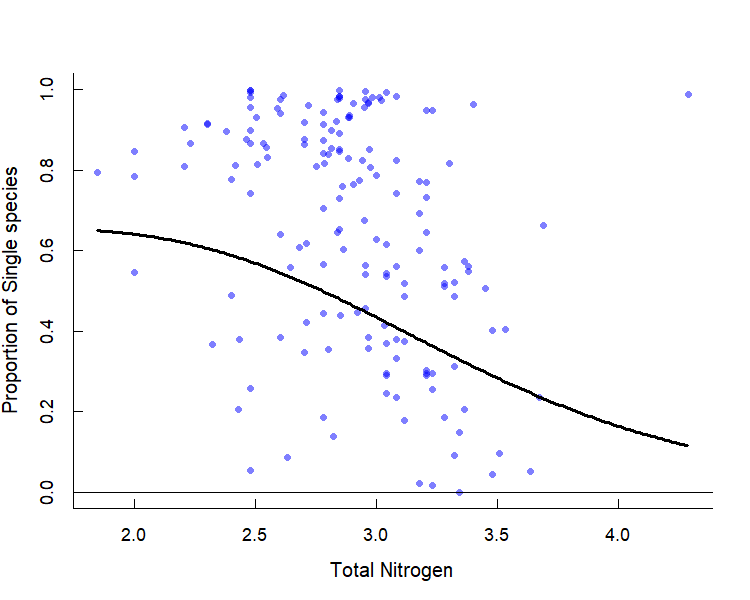 |
| Filamentous | 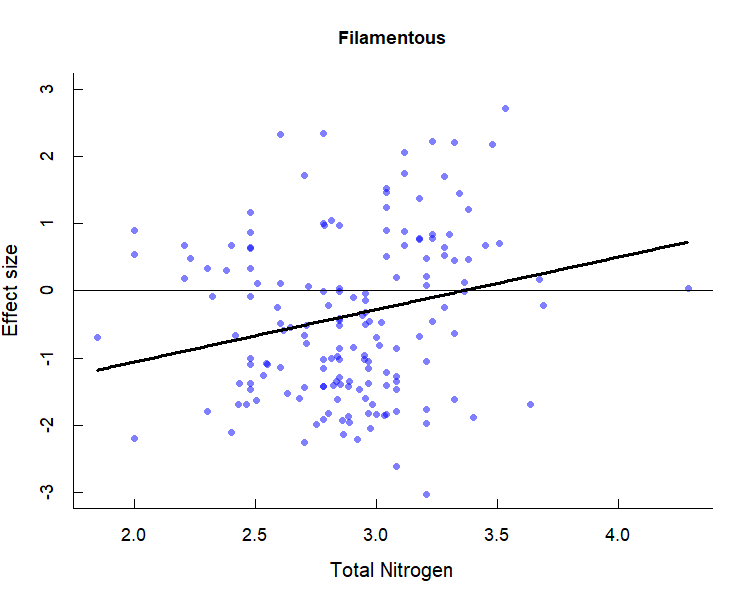 | 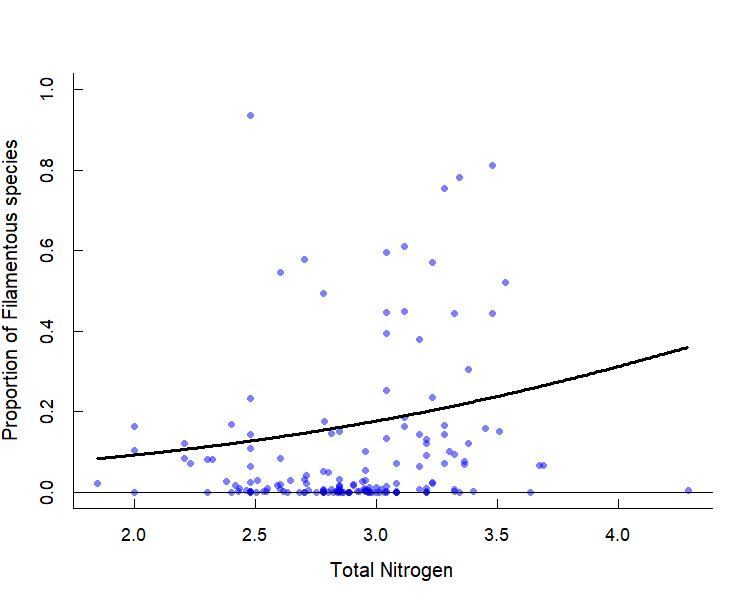 |
| Mixotrophic | 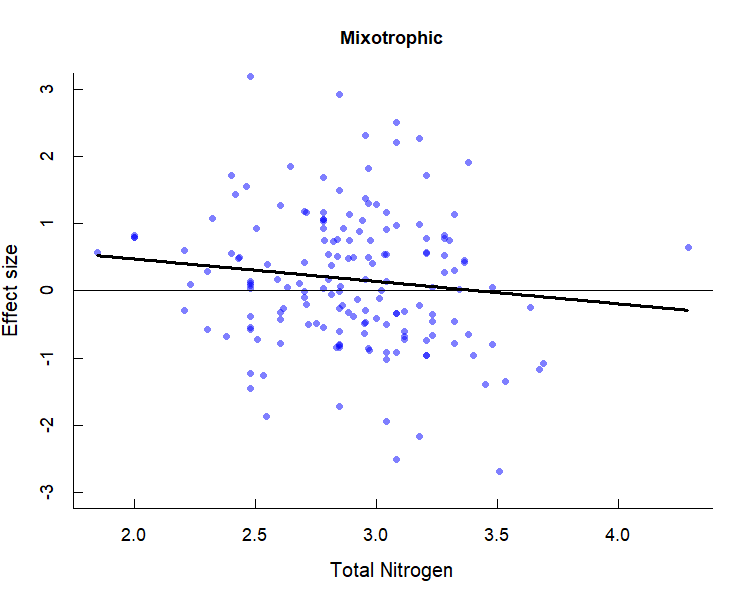 | 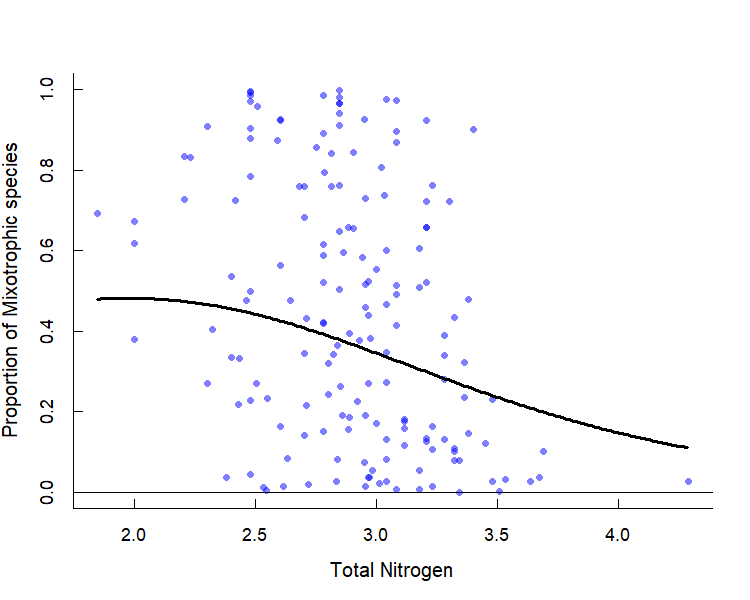 |
| Silicious | 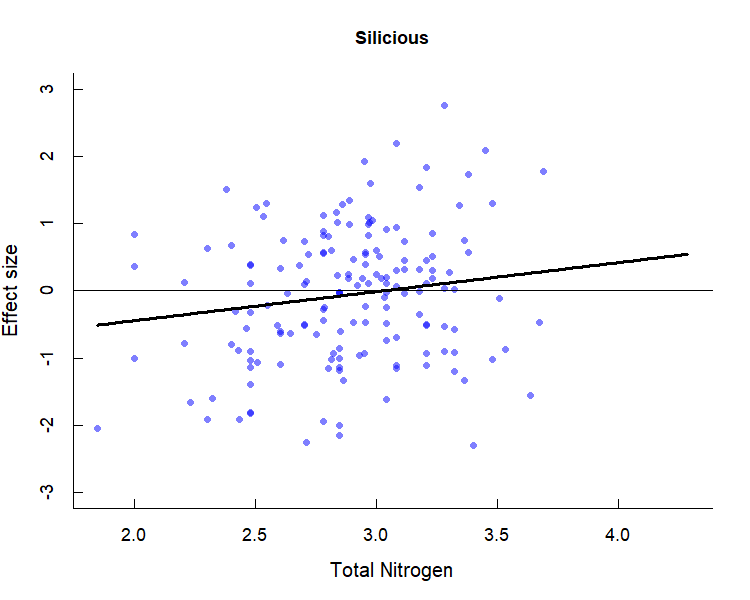 | 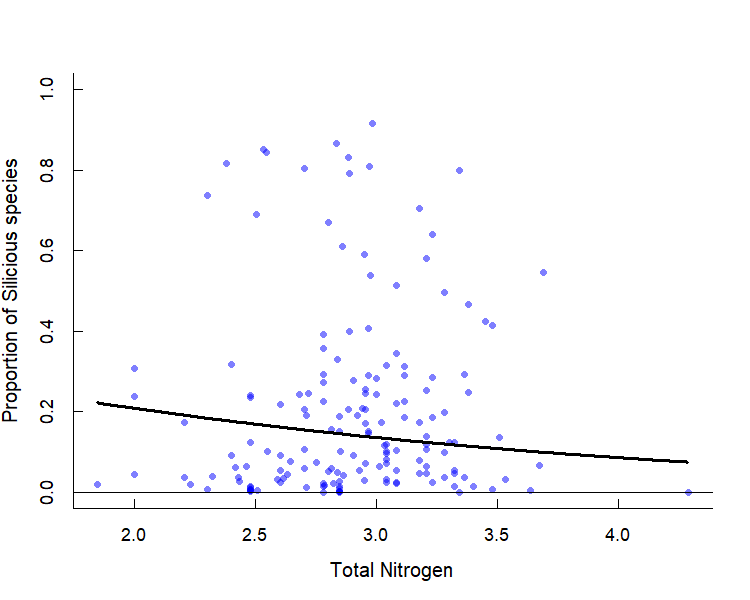 |
| Nitrogen-fixing | 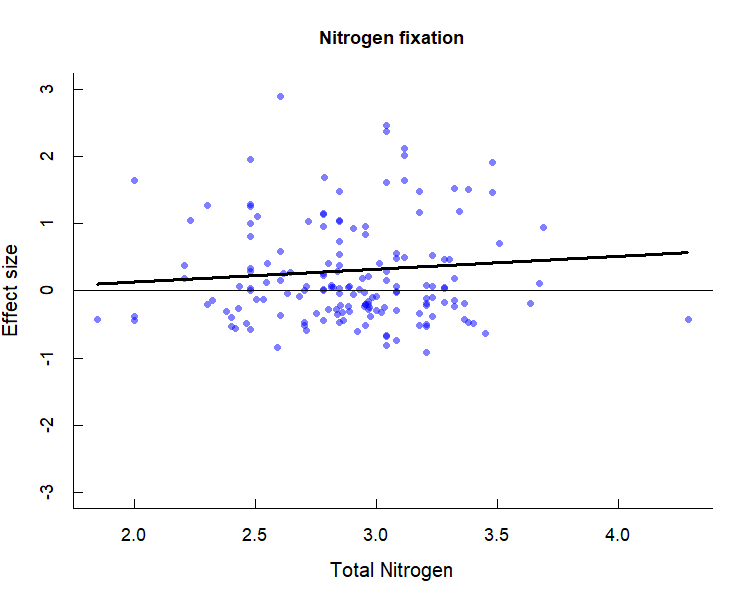 | 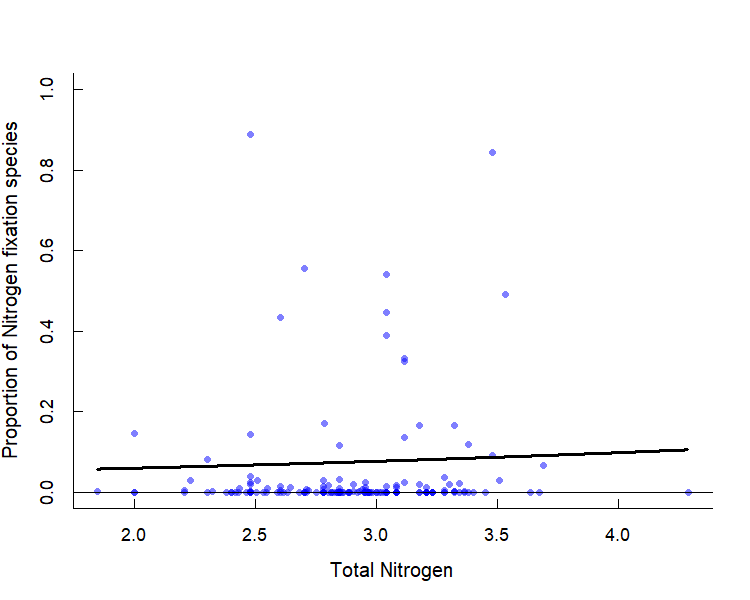 |
| Vacuolated | 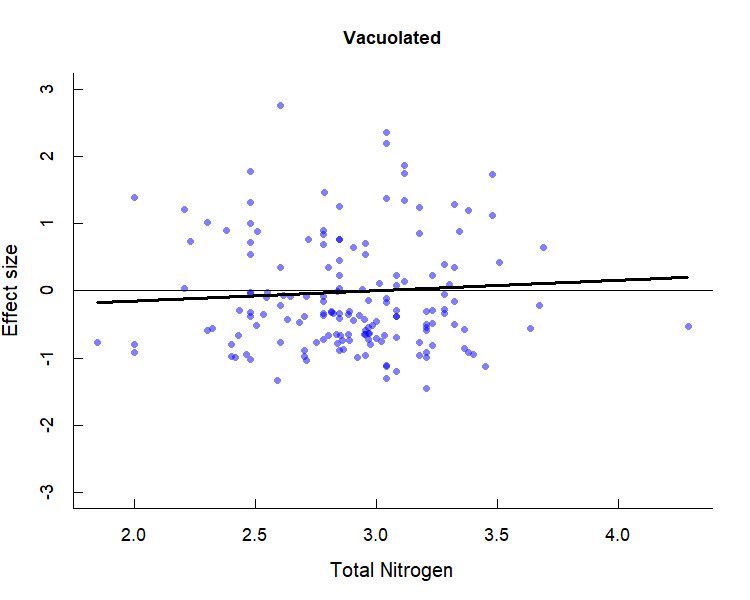 | 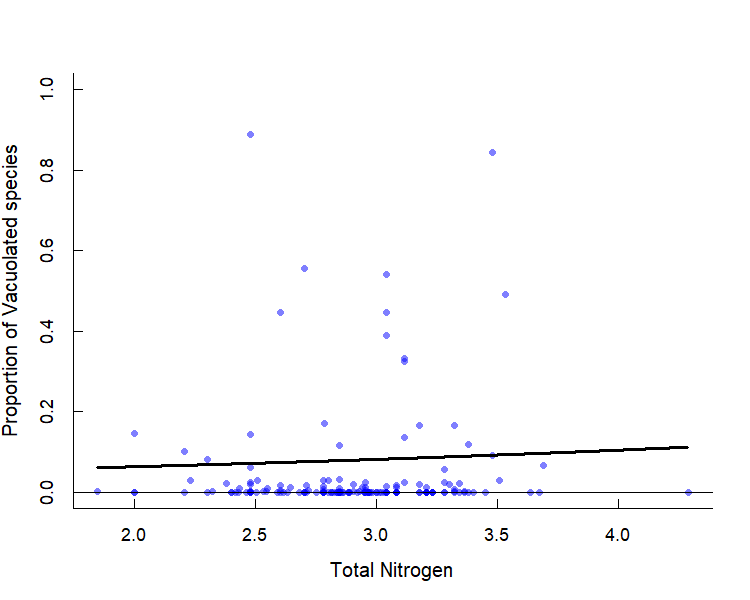 |
| Large Flagellated | 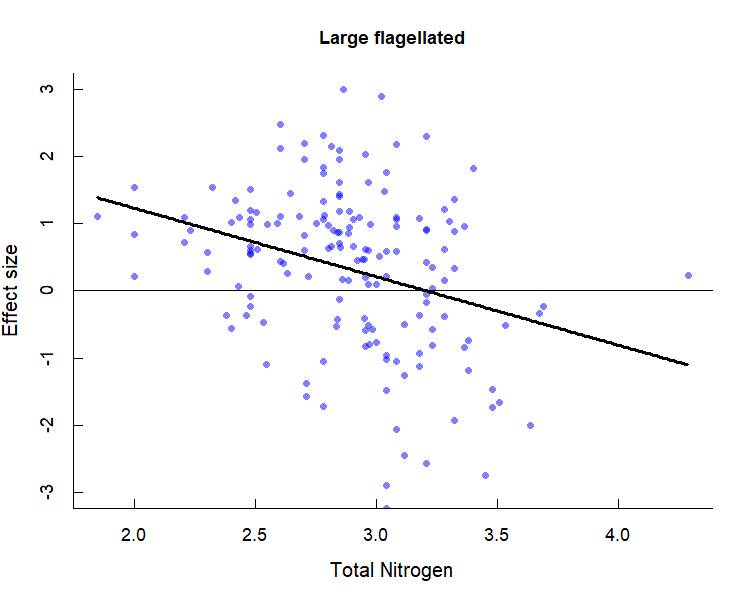 | 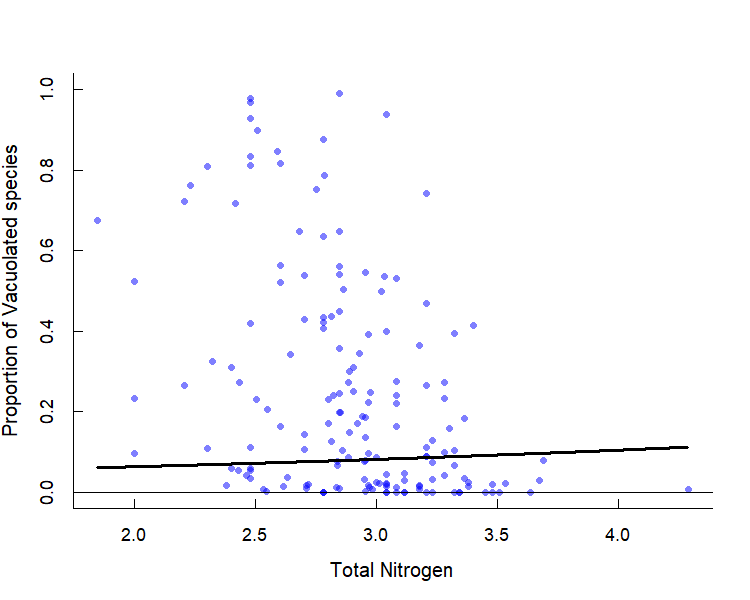 |

Figure S5. Distribution of the ES values and distribution of the CWM values against Algal biomass (log (mgL^-1^). Each dot represents a sample on the gradient. Curves indicate the GAM models’ trendlines.

| Traits | Distribution of the ES values against the variables | Distribution of the CWM values against the variables |
| --- | --- | --- |
|  |  |  |
| Flagellated | 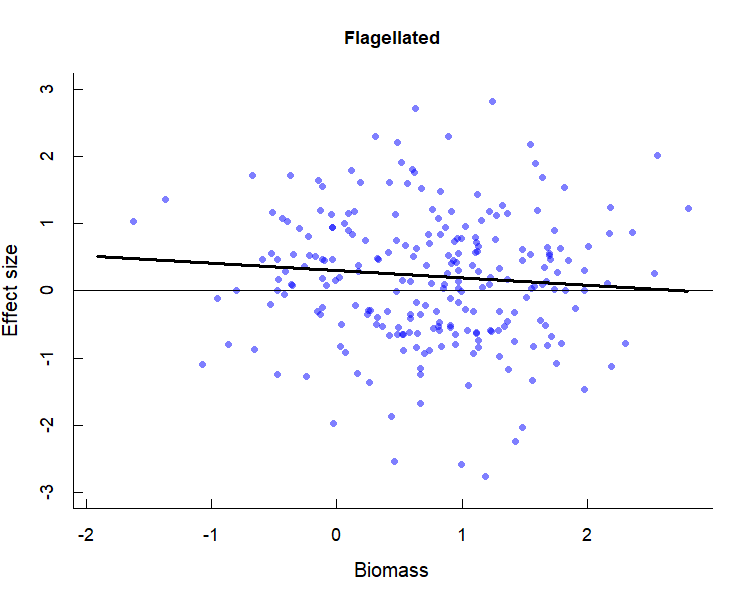 | 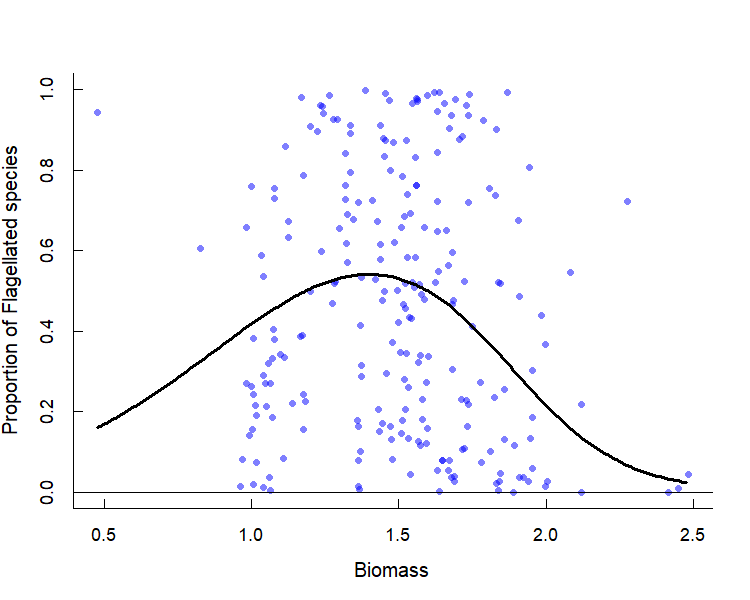 |
| Size  (larger >40 µm) | 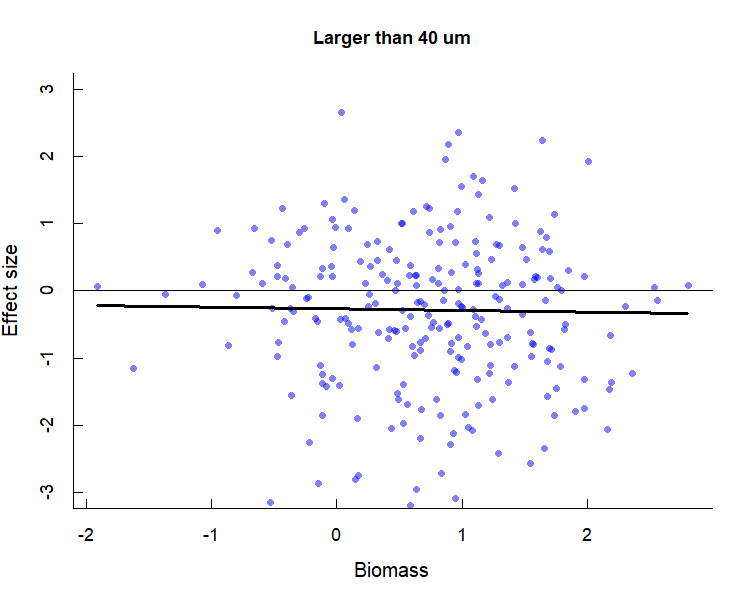 | 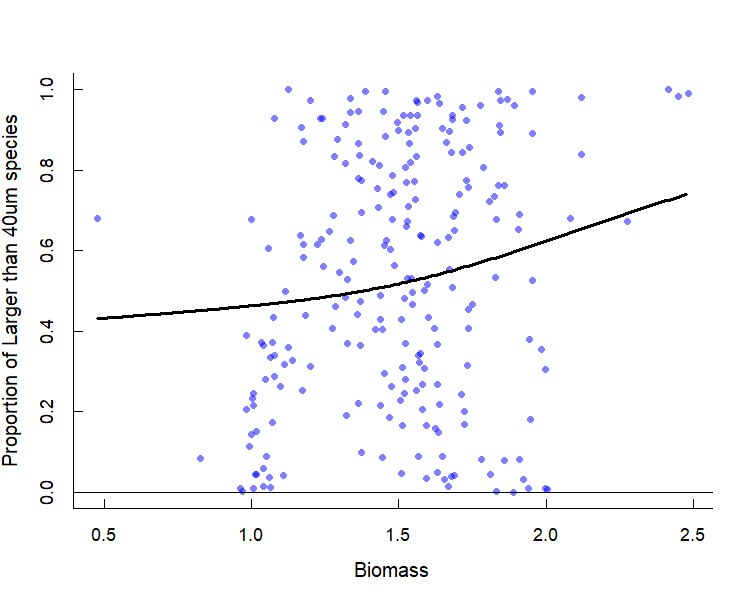 |
| Colonial | 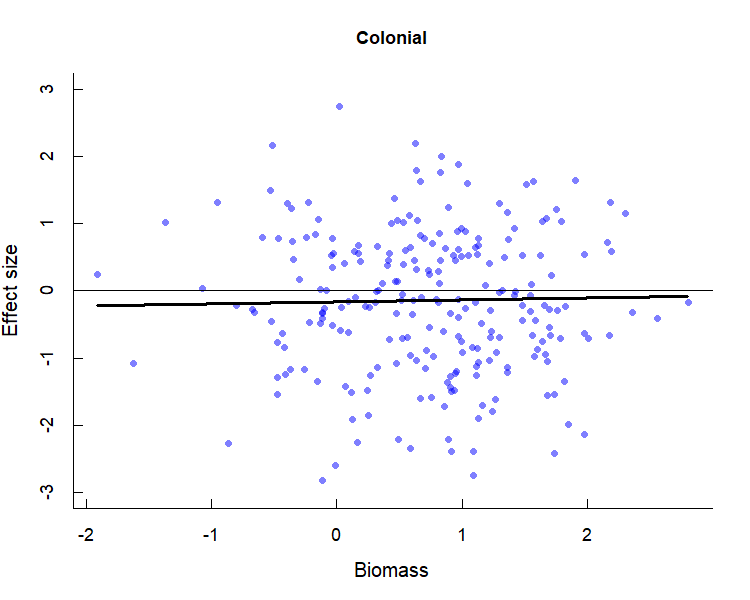 | 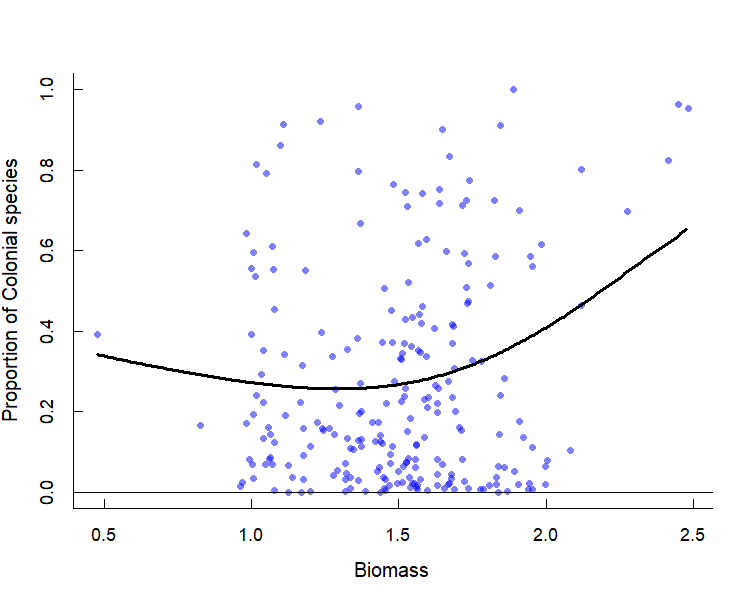 |
| Single celled | 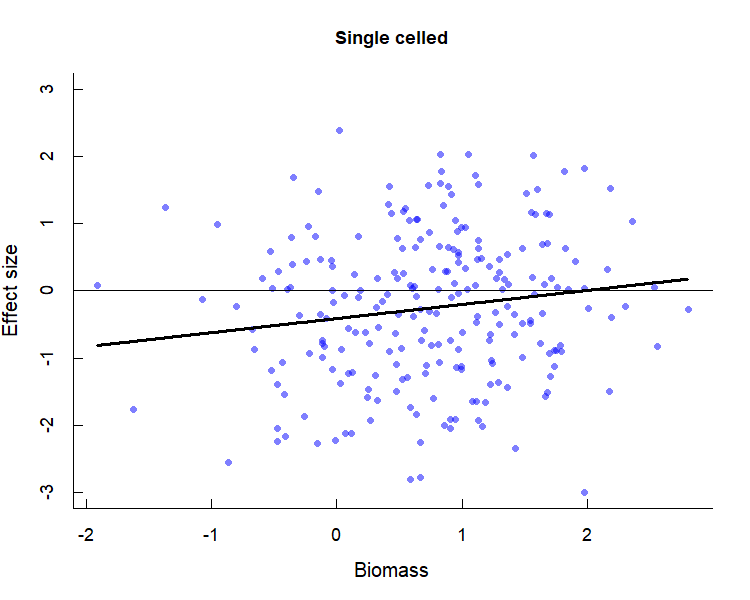 | 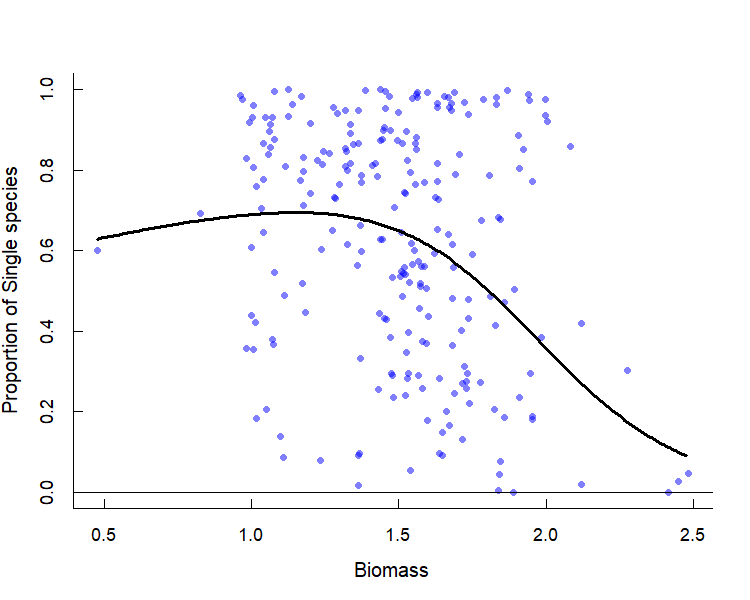 |
| Filamentous | 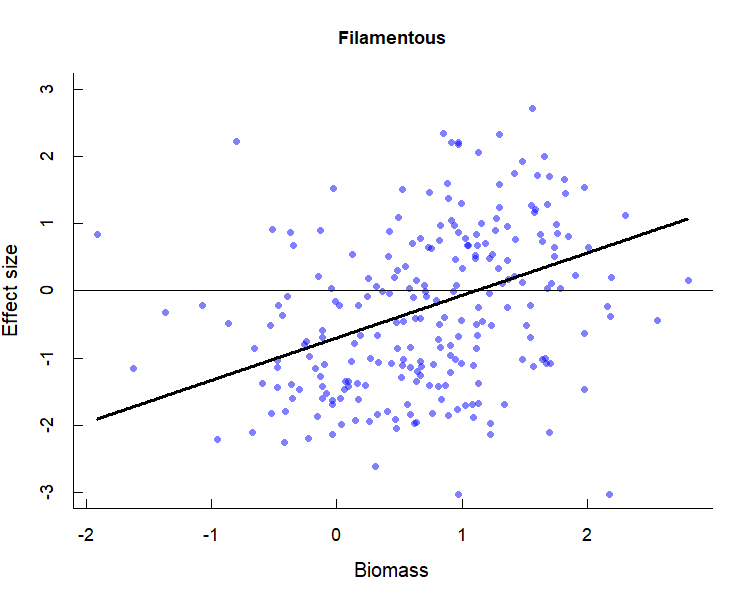 | 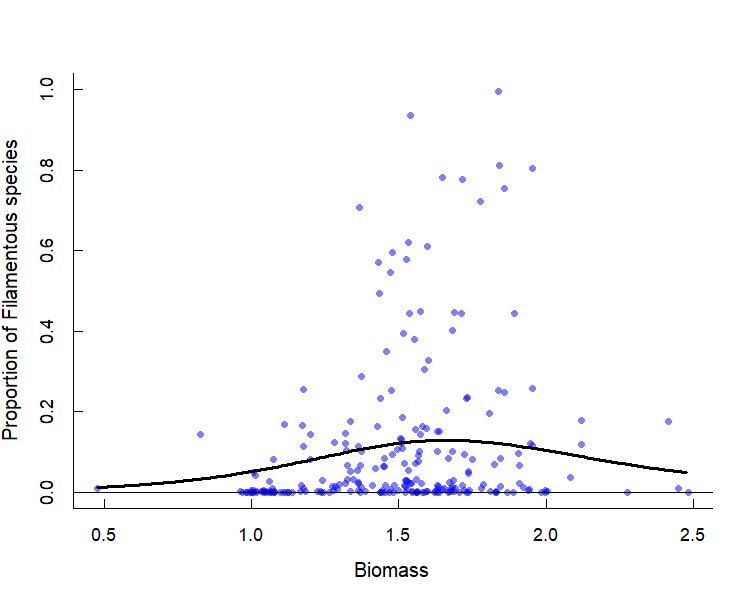 |
| Mixotrophic | 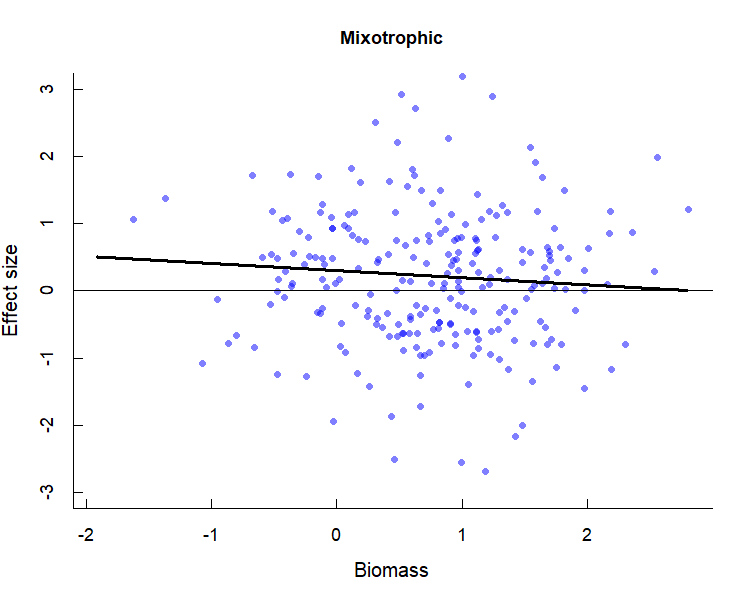 | 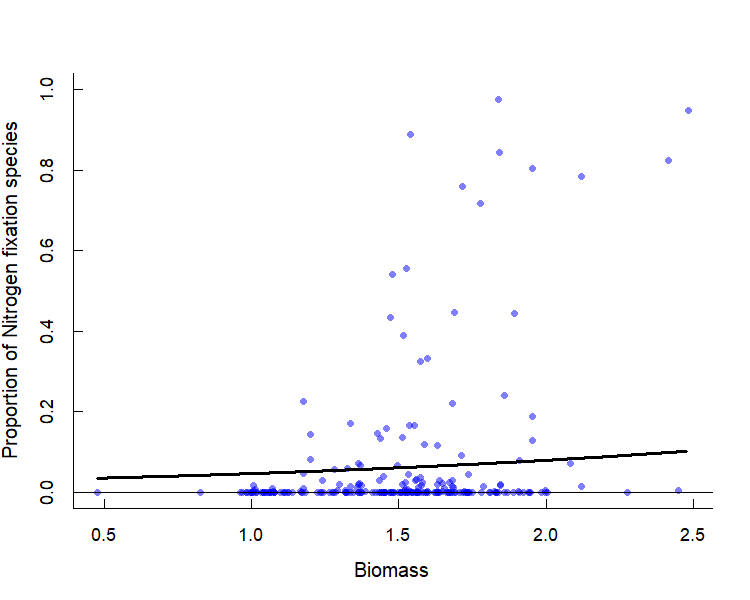 |
| Silicious | 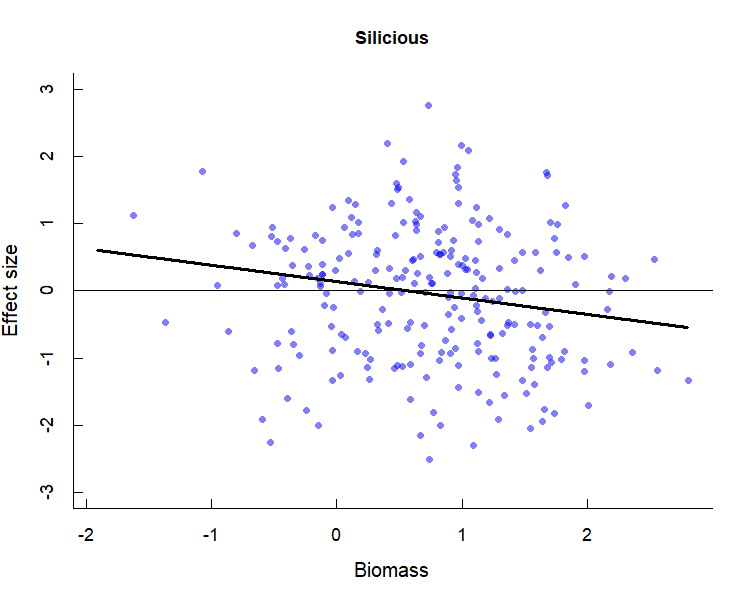 | 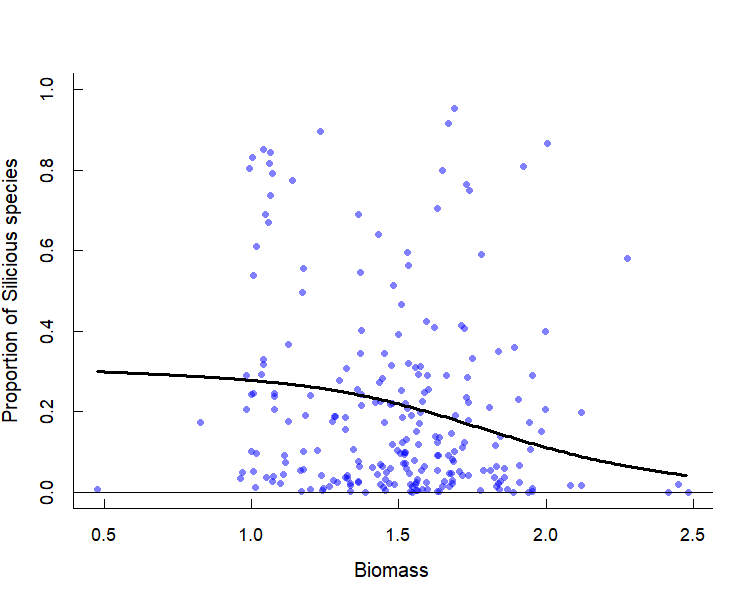 |
| Nitrogen-fixing | 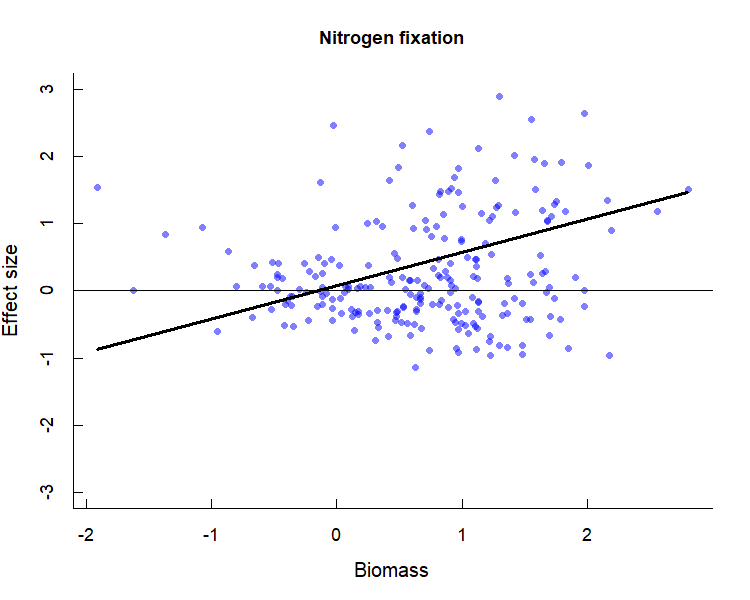 | 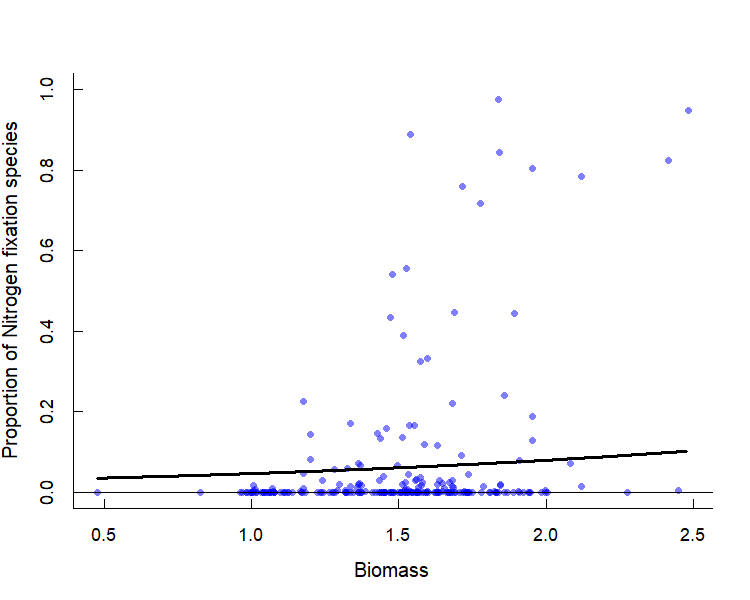 |
| Vacuolated | 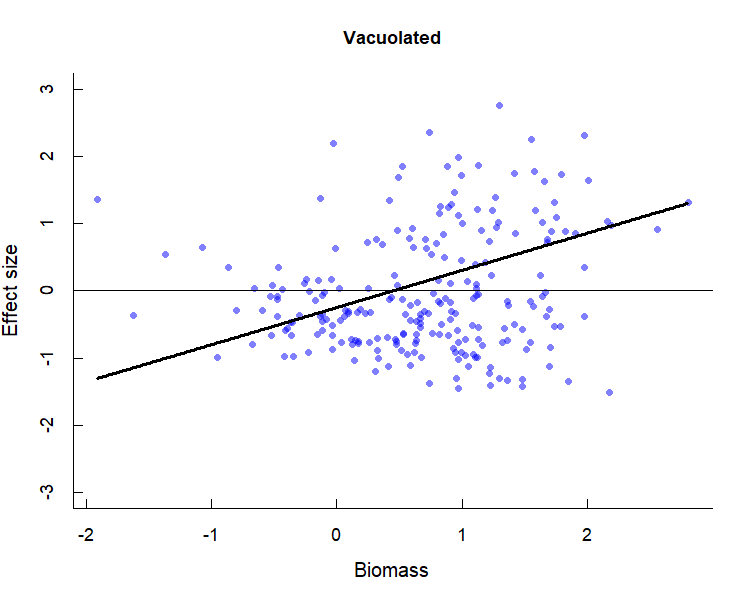 | 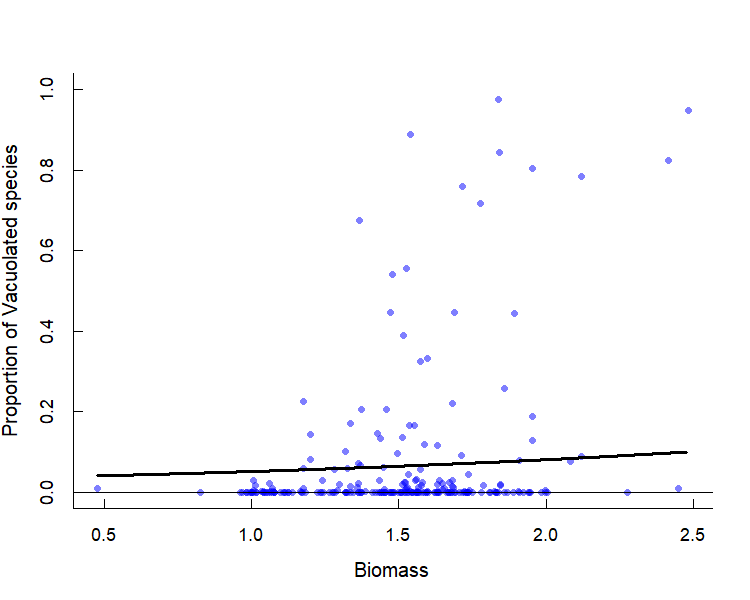 |
| Large Flagellated | 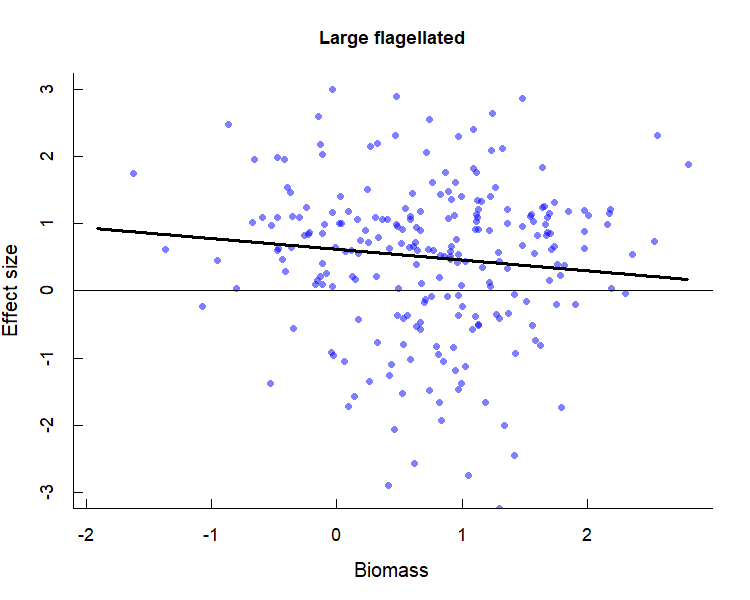 | 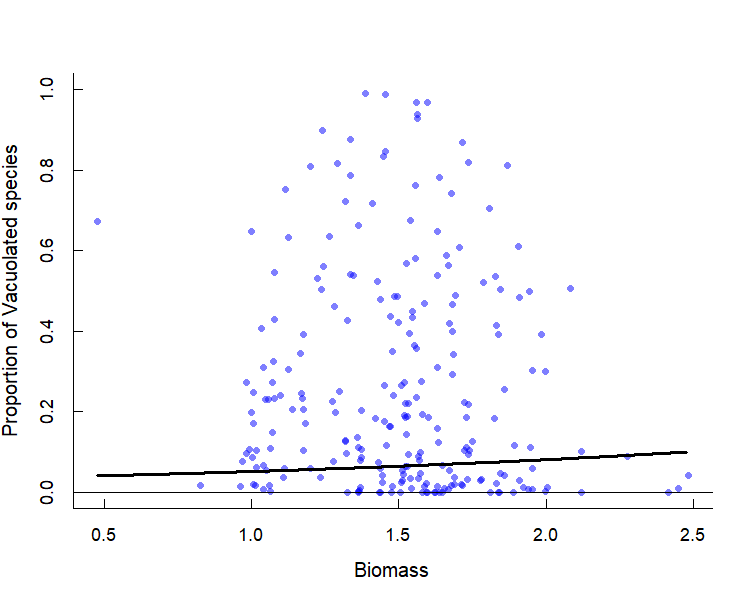 |

Figure S6. Distribution of the ES and CWM values against Chlorophyll-*a* (log (mgL^-1^). Each dot represents a sample on the gradient. Curves indicate the GAM models’ trendlines.

| Traits | Distribution of the ES values against the variables | Distribution of the CWM values against the variables |
| --- | --- | --- |
|  |  |  |
| Flagellated | 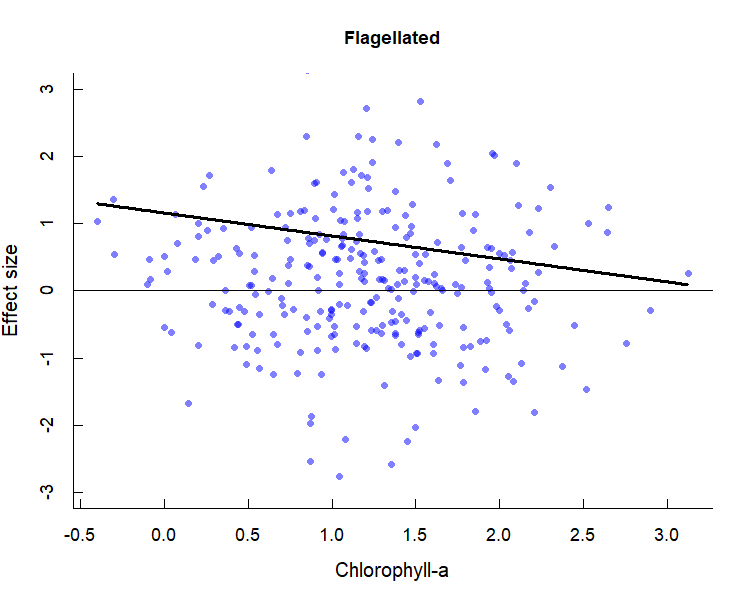 | 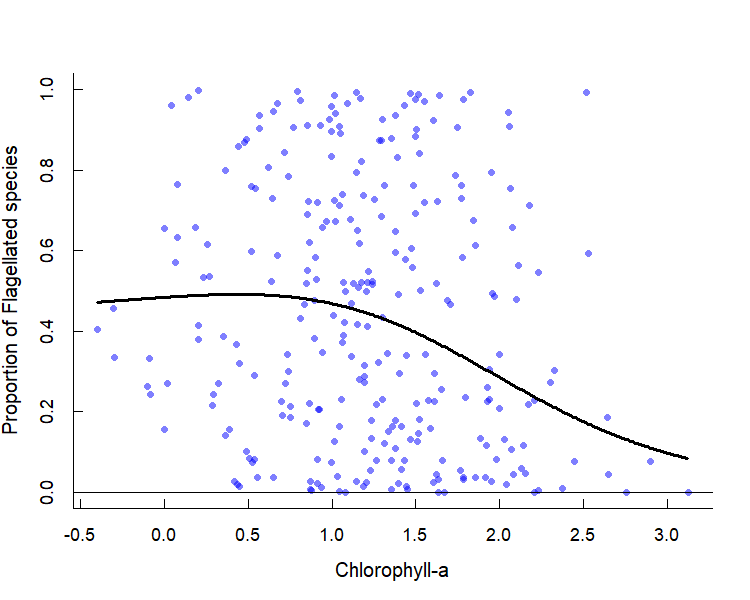 |
| Size (larger >40 µm) | 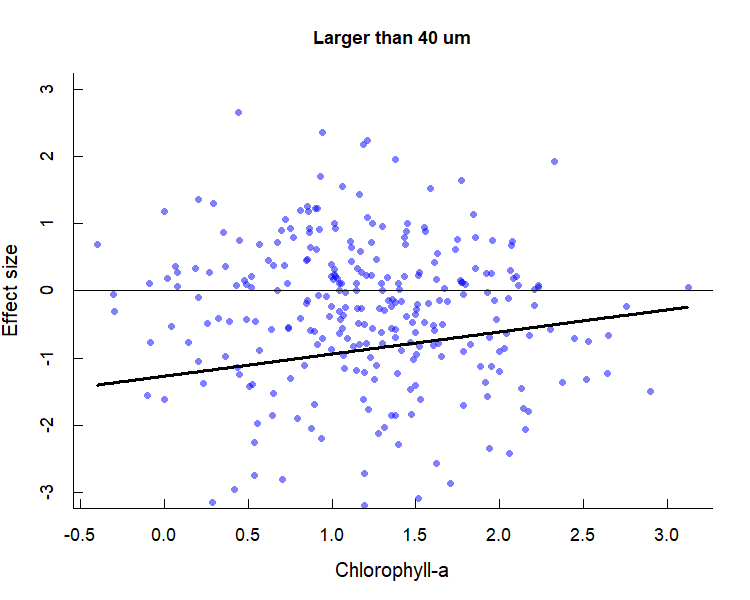 | 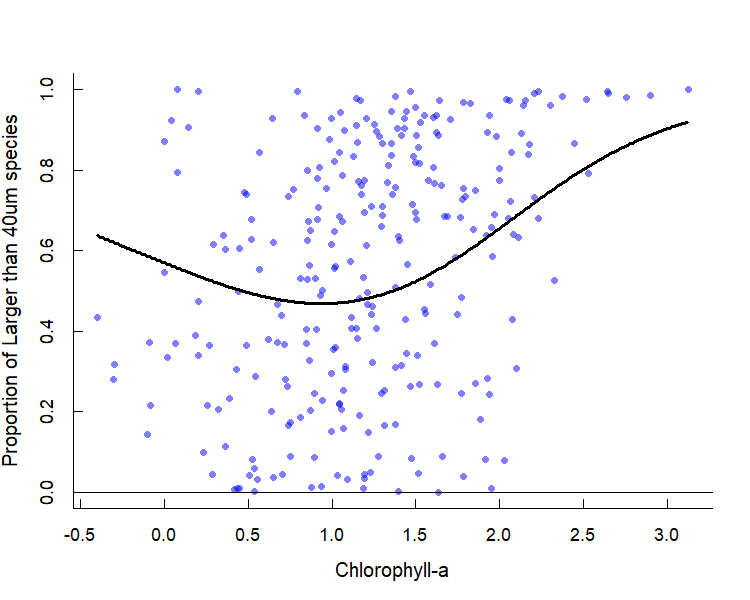 |
| Colonial | 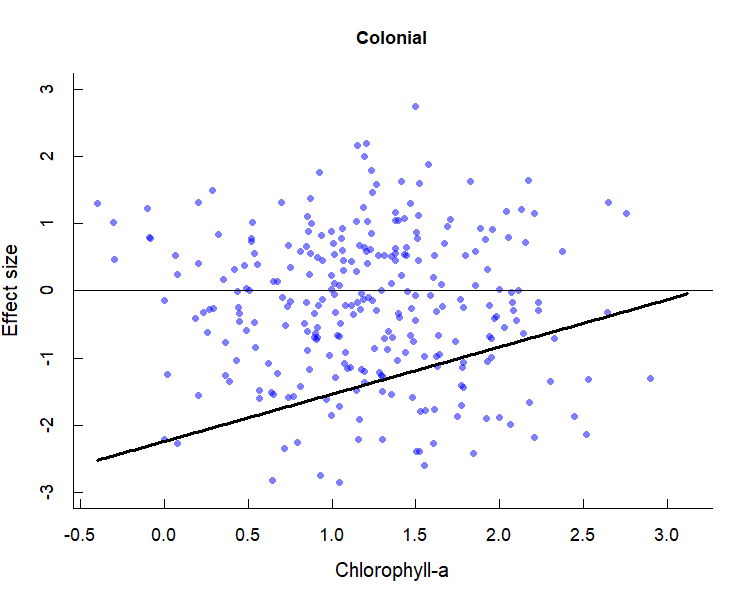 | 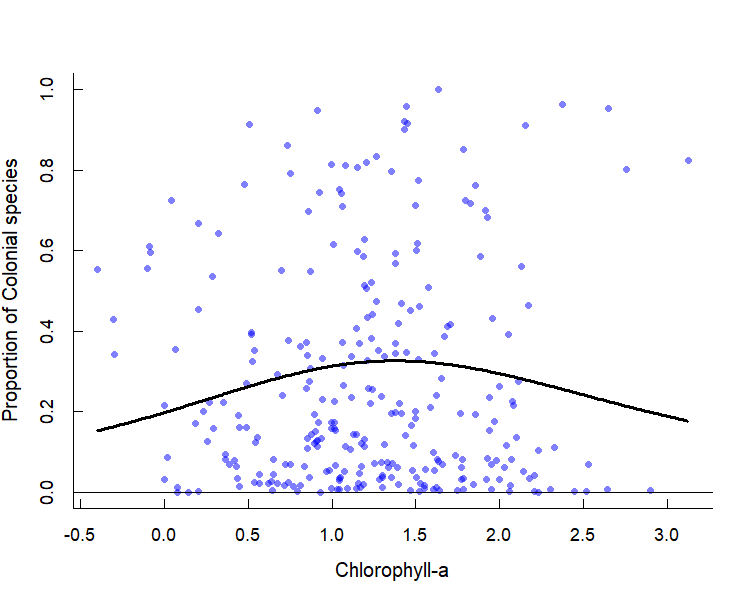 |
| Single celled | 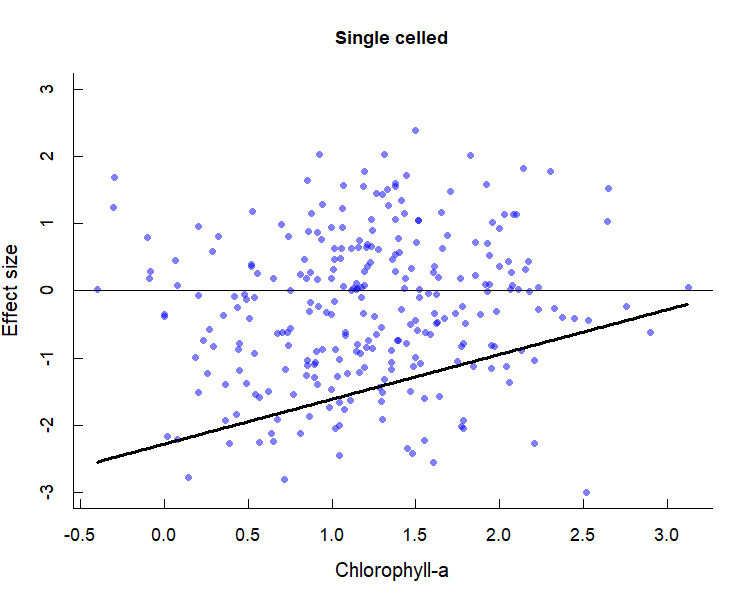 | 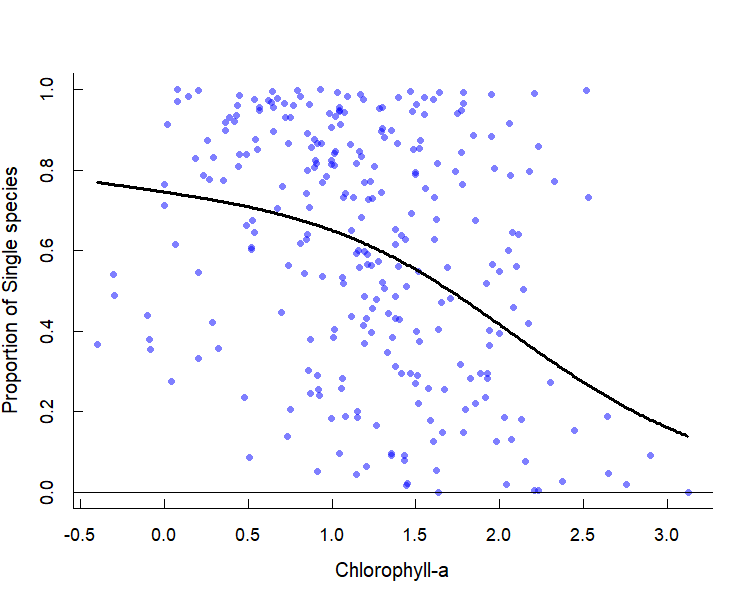 |
| Filamentous | 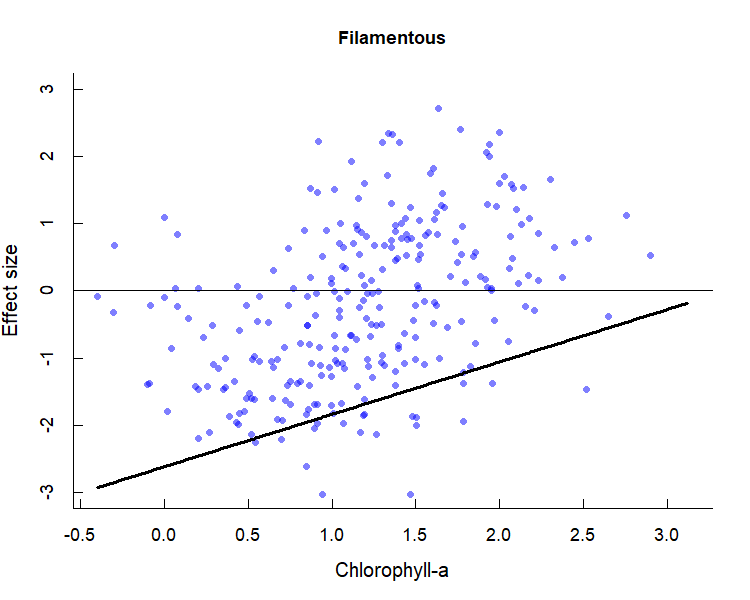 | 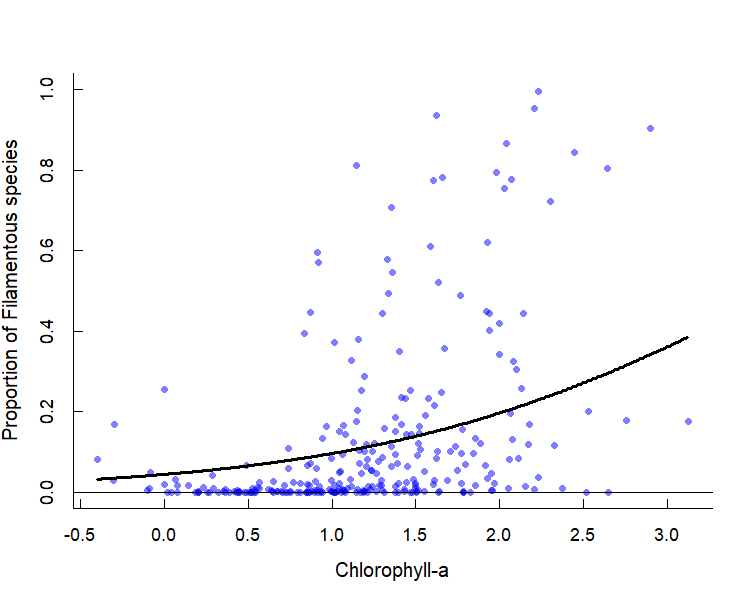 |
| Mixotrophic | 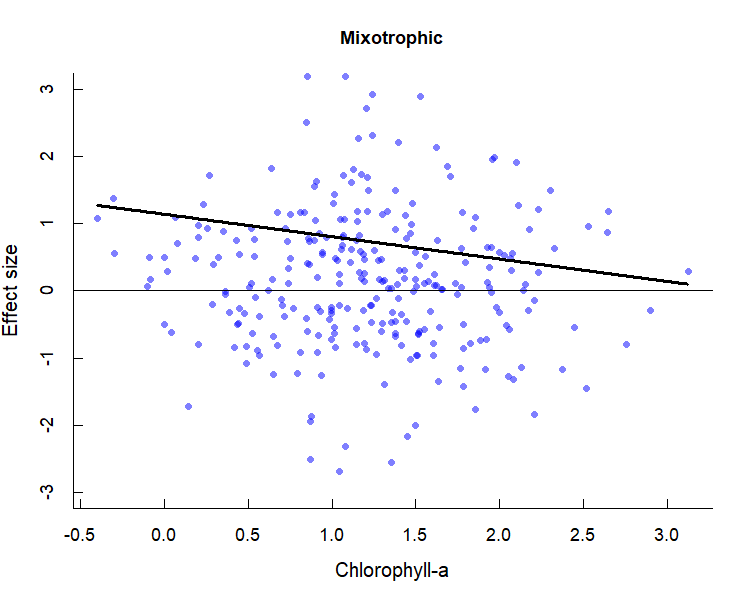 | 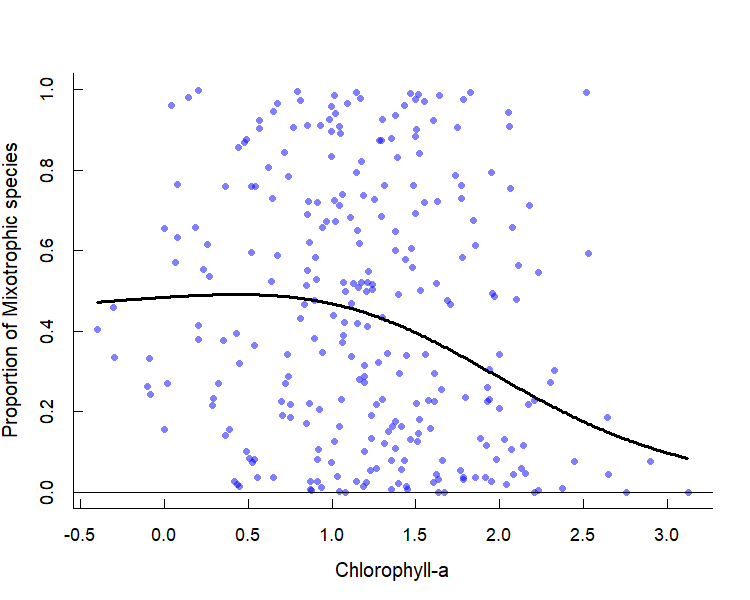 |
| Silicious | 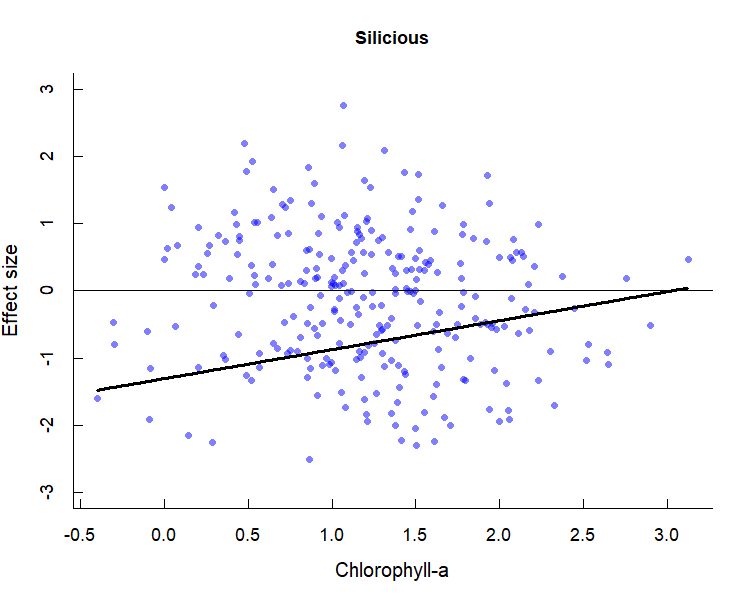 | 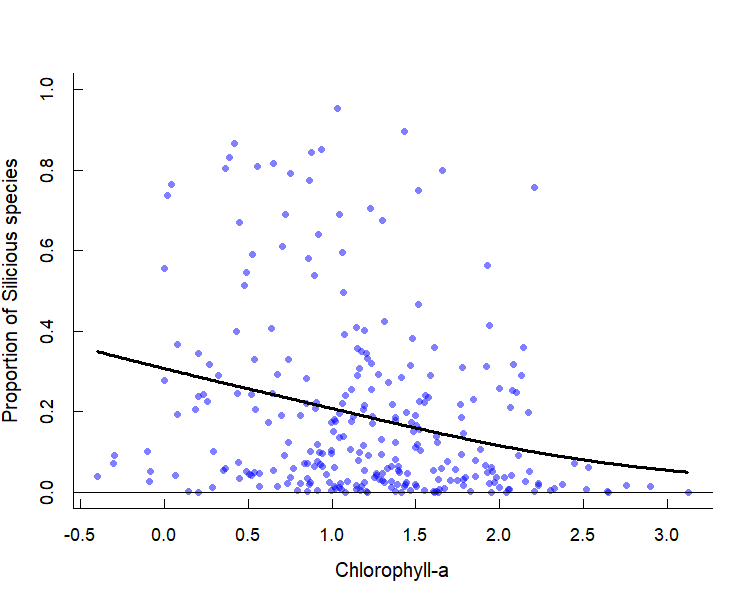 |
| Nitrogen-fixing | 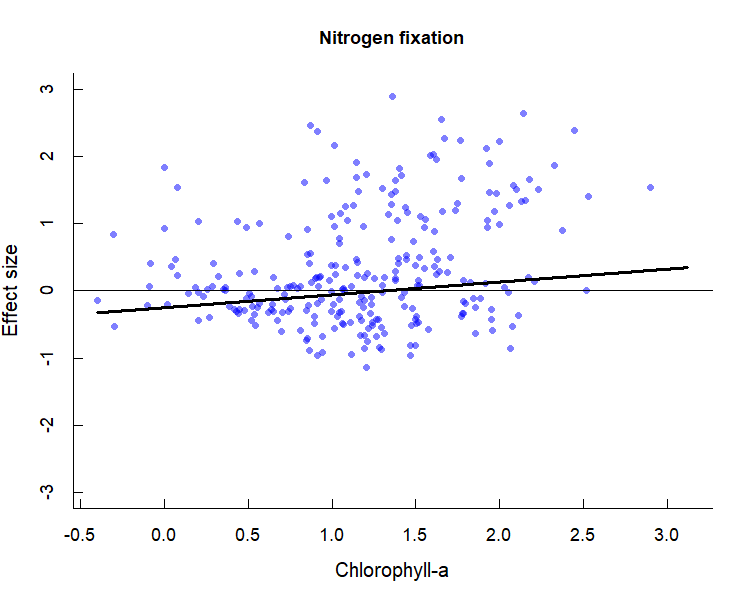 | 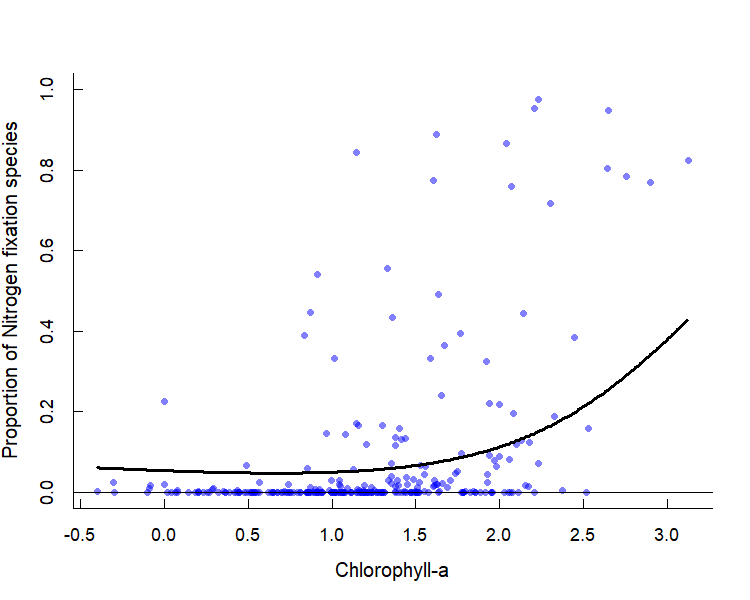 |
| Vacuolated | 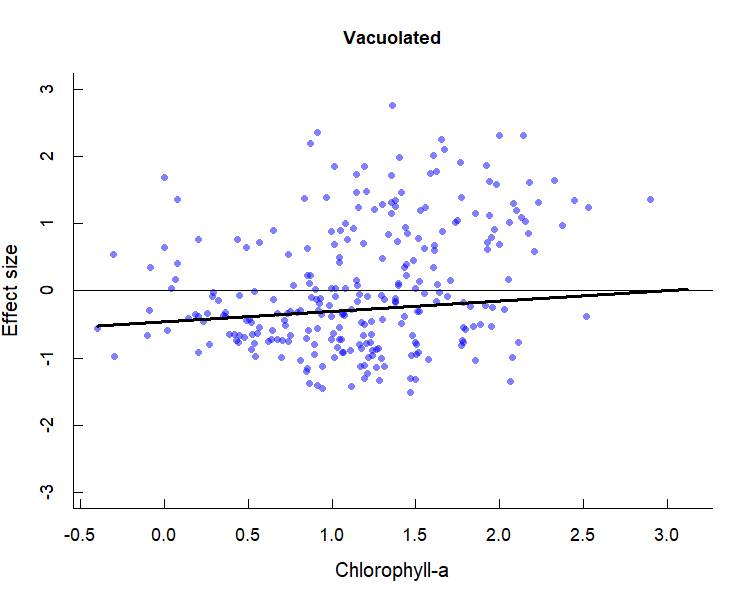 | 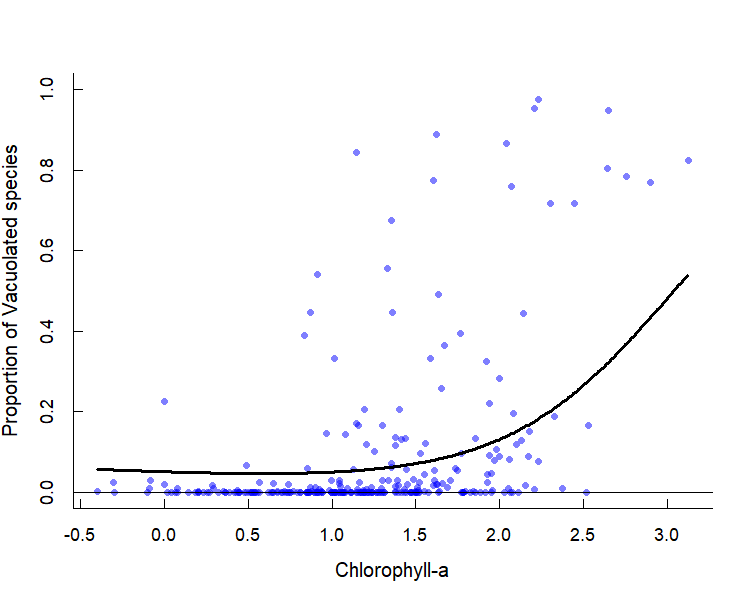 |
| Large Flagellated | 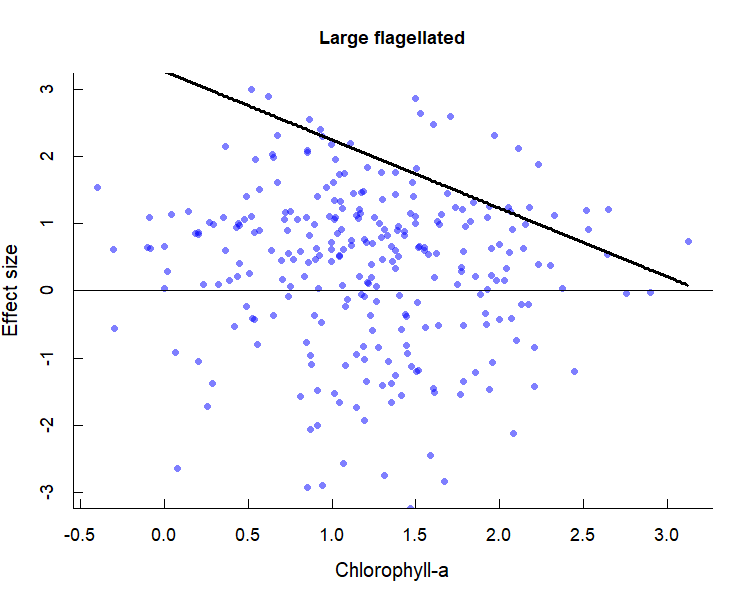 | 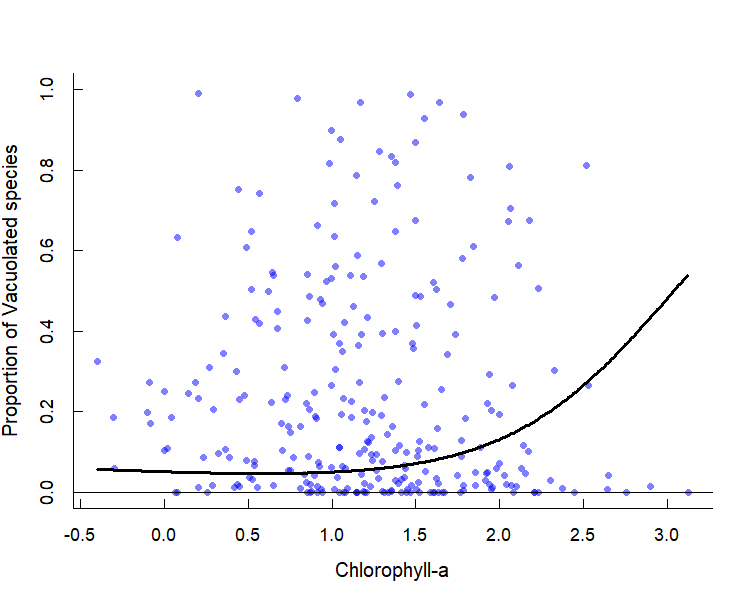 |

Figure S7. Distribution of the ES values and distribution of the CWM values against Chemical Oxigen Demand(COD) (log (mgL^-1^). Each dot represents a sample on the gradient. Line indicate the GAM model trendline.

| Traits | Distribution of the ES values against the variables | Distribution of the CWM values against the variables |
| --- | --- | --- |
|  |  |  |
| Flagellated |  |  |
| Size (larger >40 µm) |  |  |
| Colonial |  |  |
| Single celled |  |  |
| Filamentous |  |  |
| Mixotrophic |  |  |
| Silicious |  |  |
| Nitrogen-fixing |  |  |
| Vacuolated |  |  |
| Large Flagellated |  |  |

Figure S8. Distribution of the ES values and distribution of the CWM values against Secchi depth(log(m)). Each dot represents a sample on the gradient. Curves indicate the GAM models’ trendlines.

| Traits | Distribution of the ES values against the variables | Distribution of the CWM values against the variables |
| --- | --- | --- |
|  |  |  |
| Flagellated |  |  |
| Size (larger >40 µm) |  |  |
| Colonial |  |  |
| Single celled |  |  |
| Filamentous |  |  |
| Mixotrophic |  |  |
| Silicious |  |  |
| Nitrogen-fixing |  |  |
| Vacuolated |  |  |
| Large Flagellated |  |  |

Figure S9. Distribution of the ES values and distribution of the CWM values against pH. Each dot represents a sample on the gradient. Curves indicate the GAM models’ trendlines.

| Traits | Distribution of the ES values against the variables | Distribution of the CWM values against the variables |
| --- | --- | --- |
|  |  |  |
| Flagellated |  |  |
| Size (larger >40 µm) |  |  |
| Colonial |  |  |
| Single celled |  |  |
| Filamentous |  |  |
| Mixotrophic |  |  |
| Silicious |  |  |
| Nitrogen-fixing |  |  |
| Vacuolated |  |  |
| Large Flagellated |  |  |

Figure S10. Distribution of the ES values and distribution of the CWM values against Conductivity(log(Cond). Each dot represents a sample on the gradient. Curves indicate the GAM models’ trend lines.

| Traits | Distribution of the ES values against the variables | Distribution of the CWM values against the variables |
| --- | --- | --- |
|  |  |  |
| Flagellated |  |  |
| Size (larger >40 µm) |  |  |
| Colonial |  |  |
| Single celled |  |  |
| Filamentous |  |  |
| Mixotrophic |  |  |
| Silicious |  |  |
| Nitrogen-fixing |  |  |
| Vacuolated |  |  |
| Large Flagellated |  |  |
